# Naturally occurring plant-based anticancerous candidates as prospective ABCG2 inhibitors: an in silico drug discovery study

**DOI:** 10.1007/s11030-022-10389-6

**Published:** 2022-02-28

**Authors:** Mahmoud A. A. Ibrahim, Alaa H. M. Abdelrahman, Esraa A. A. Badr, Nahlah Makki Almansour, Othman R. Alzahrani, Muhammad Naeem Ahmed, Mahmoud E. S. Soliman, Mohamed Ahmed Naeem, Ahmed M. Shawky, Peter A. Sidhom, Gamal A. H. Mekhemer, Mohamed A. M. Atia

**Affiliations:** 1 Computational Chemistry Laboratory, Chemistry Department, Faculty of Science, Minia University, Minia, 61519 Egypt; 2 Department of Biology, College of Science, University of Hafr Al Batin, Hafr Al Batin, 1803 Saudi Arabia; 3 Department of Biology, Faculty of Sciences, University of Tabuk, Tabuk, 71491 Saudi Arabia; 4 Department of Chemistry, The University of Azad Jammu and Kashmir, Muzaffarabad, 13100 Pakistan; 5 Molecular Modelling and Drug Design Research Group, School of Health Sciences, University of KwaZulu-Natal, Westville, 4000 Durban South Africa; 6 Ain Shams University Specialized Hospital, Ain Shams University, Cairo, Egypt; 7 Science and Technology Unit (STU), Umm Al-Qura University, Makkah, 21955 Saudi Arabia; 8 Department of Pharmaceutical Chemistry, Faculty of Pharmacy, Tanta University, Tanta, 31527 Egypt; 9 Molecular Genetics and Genome Mapping Laboratory, Genome Mapping Department, Agricultural Genetic Engineering Research Institute (AGERI), Agricultural Research Center (ARC), Giza, 12619 Egypt

**Keywords:** ABCG2, Multidrug resistance, NPACT database, Molecular docking, Molecular dynamics simulations

## Abstract

**Abstract:**

ATP-binding cassette transporter G2 (ABCG2) is an efflux transporter related to the clinical multidrug resistance (MDR) phenomenon. Identifying ABCG2 inhibitors could help discover extraordinary curative strategies for carcinoma remediation. Hitherto, there is no medication drug inhibiting ABCG2 transporter, notwithstanding that a considerable number of drugs have been submitted to clinical-trial and investigational phases. In the search for unprecedented chemical compounds that could inhibit the ABCG2 transporter, an in silico screening was conducted on the Naturally Occurring Plant-based Anticancer Compound-Activity-Target (NPACT) database containing 1574 compounds. Inhibitor-ABCG2 binding affinities were estimated based on molecular docking and molecular minimization (MM) calculations and compared to a co-crystallized inhibitor (BWQ) acting as a reference inhibitor. Molecular dynamics (MD) simulations pursued by molecular mechanics-generalized Born surface area (MM-GBSA) binding energy estimations were further executed for compounds with MM-GBSA//MM binding energies lower than BWQ (calc. − 60.5 kcal/mol). NPACT00968 and NPACT01545 demonstrated auspicious inhibitory activities according to binding affinities (Δ*G*_binding_) over the 100 ns MD simulations that were nearly one and a half folds compared to BWQ (− 100.4, − 94.7, and − 62.9 kcal/mol, respectively). Throughout the 100 ns MD simulations, structural and energetical analyses unveiled outstanding stability of the ABCG2 transporter when bound with NPACT00968 and NPACT01545. In silico calculations hold a promise for those two inhibitors as drug candidates of ABCG2 transporter and emphasize that further in vitro and in vivo experiments are guaranteed.

**Graphical abstract:**

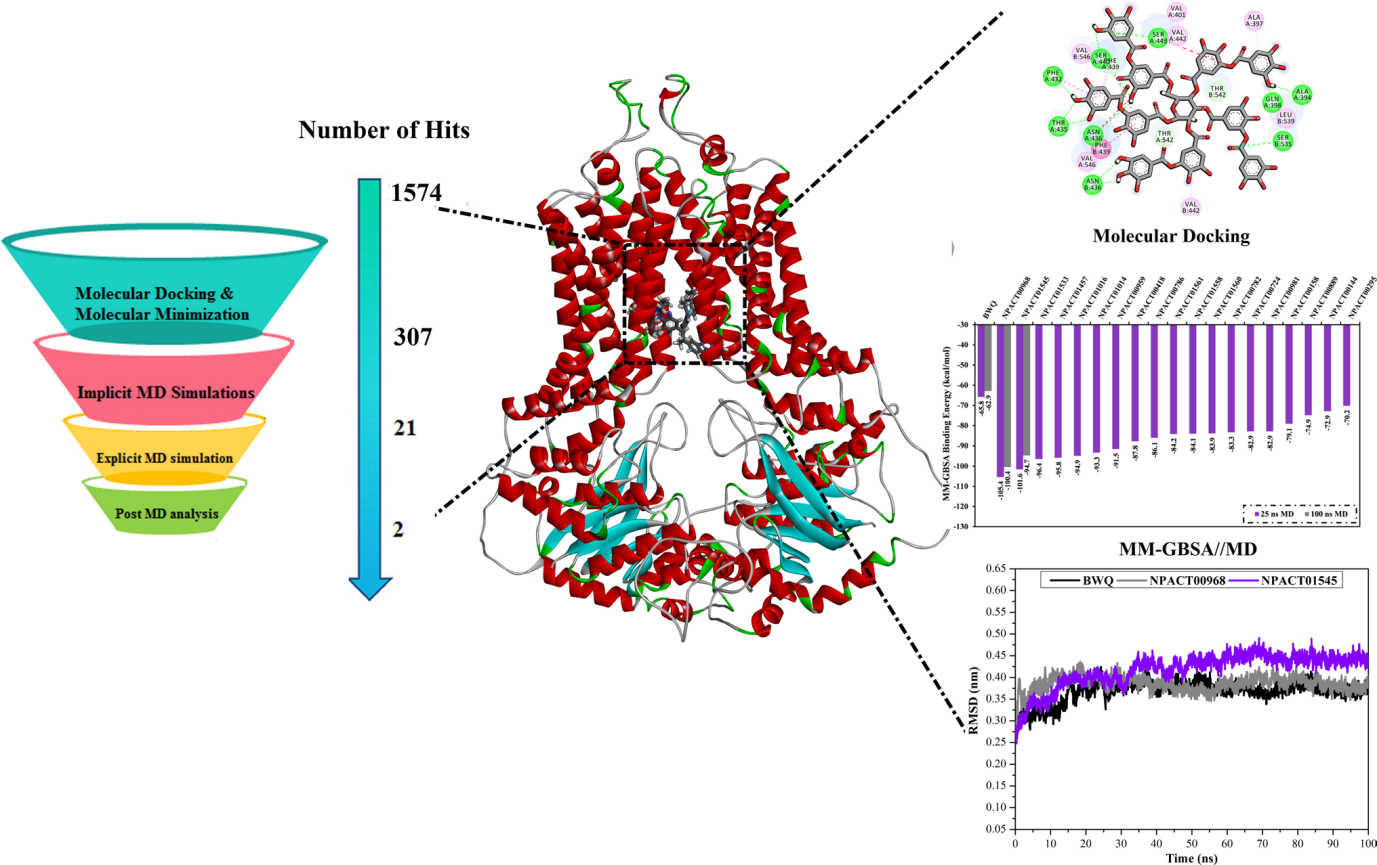

**Supplementary Information:**

The online version contains supplementary material available at 10.1007/s11030-022-10389-6.

## Introduction

In the last decades, multidrug resistance (MDR) has been one of the leading troubles that confront clinical remediation, particularly chemotherapy for different types of cancer [1]. One of the most pivotal drug resistance factors is linked to the ATP-binding cassette (ABC) transporters present on plasma membranes [2]. Up to now, forty-eight human ABC transporters have been pinpointed and categorized into seven subfamilies (ABCA through ABCG) according to sequence resemblance [3]. Additionally, ABC transporters are the causative agents for the drug absorption, distribution, metabolism, excretion, and toxicity (ADMET) properties [4]. ABCG2 (the second member of the ABCG subfamily) plays an intrinsic role in inducing MDR inside the cancer cells [5, 6]. In normal human tissues, the ABCG2 transporter is presented in various tissues such as endothelial cells of the blood-brain barrier, small intestine, and the epithelial cells’ apical membrane [7, 8]. Indeed, the ABCG2 transporter acts as a homodimer on cellular plasma membranes [9]. Therefore, the ABCG2 transporter has a critical role in detoxification and safeguarding against cytotoxic factors via effluxing xenobiotics from the cells [10]. In cancer cells, ABCG2 is overexpressed, leading to cancer cells being resistant to chemotherapy agents. No drug has been approved in countering the ABCG2-mediated MDR yet. Consequently, exploring novel ABCG2 inhibitors could enhance the bioavailability of the anticancer drugs, lessen MDR, and result in more efficient treatment [11].

A great deal of previous research has been conducted on the structure and impacts of inhibitors on ABCG2 transporter [12, 13]. A recent study has shown that the flat ring structure of tariquidar and propafenone derivatives improves ABCG2’s inhibitory activity [14]. Data from several studies suggested that the BMS-599626, imatinib, sitravatinib, mitoxantrone, and PZ-39 are prospective ABCG2 drug candidates via different mechanisms [15–18]. While virtual screening of clinical and investigational drugs as ABCG2 inhibitors revealed that pibrentasvir, venetoclax, and ledipasvir would be promising inhibitors (calc. *k*_i_ = 1.14 nM, 16.4 nM, and 8.19 pM, respectively) toward ABCG2 transporter [19]. Chemical databases were filtrated toward the ABCG2 transporter to discover prospective ABCG2 inhibitors utilizing advanced computational approaches, revealing eight potential inhibitors with binding affinities less than − 55.8 kcal/mol [20]. The discovery of naturally occurring compounds as ABCG2 inhibitors has taken its share of studies, and it has been assumed that the hydrophobic properties of natural products enhanced ABCG2 inhibitory activity and can combat MDR [21–23].

In this study, a continued effort was dedicated to discovering potential naturally occurring plant-based ABCG2 inhibitors. Naturally Occurring Plant-based Anticancer Compound-Activity-Target (NPACT) database containing 1574 compounds were in silico screened against ABCG2 transporter. Binding energies of the most potent NPACT compounds complexed with ABCG2 transporter were estimated throughout the 100 ns molecular dynamics (MD) simulations utilizing molecular mechanics-generalized born surface area (MM-GBSA) approach. The structural and energetical constancies of the top potent NPACT compounds in complex with ABCG2 transporter were then inspected over the 100 ns MD course. The current study sheds light on the potentiality of NPACT compounds as prospective drug candidates to vanquish BCRP-mediated MDR and consequently represent an efficient agent for rational discovery of modulators of other proteins.

## Computational methodology

### ABCG2 preparation

The three dimensional cryo-electron microscopy (EM) of the ABCG2 transporter bound with tert-butyl3-((3S,6S,12aS)-9-(cyclopentyloxy)-6-isobutyl-1,4-dioxo-1,2,3,4,6,7,12,12a-octahydro-pyrazino[1',2':1,6]pyrido[3,4-b]indol-3-yl)propanoate (BWQ; also known as MZ29) (PDB code: 6FFC [12]) was obtained from RCSB Protein Data Bank (https://www.rcsb.org/) as a template for all in silico calculations. The crystal structure of the ABCG2 transporter was prepared by removing hetero-atoms, crystallographic waters, and ions. All missing residues were constructed using Modeller software [24]. Additionally, the protonation states of the titratable amino acid residues were assigned using the H ++ web server [25].

### Validation of in silico protocol

The performance of the employed in silico protocol in anticipating the ligand-ABCG2 binding mode was assessed based on two experimentally resolved structures of ABCG2 transporter bound with a ligand. The two ligands were 4-(4-methyl-piperazin-1-YLmethyl)-N-[4-methyl-3-(4-pyridin-3-YL-pyrimidin-2-YLamino)-phenyl]-benzamide (imatinib/STI) and MZ29/BWQ (PDB ID: 6VXH [15] and 6FFC [12], respectively).

### Database preparation

Naturally Occurring Plant-based Anticancer Compound-Activity-Target (NPACT) database containing 1574 compounds was downloaded and prepared [26]. All compounds were obtained in 2D structural data format (SDF), and their 3D chemical structures were generated with the assistance of Omega2 software [27, 28]. The geometrical structures of the NPACT compounds were then minimized using an MMFF94S force field with the help of SZYBKI software [29, 30]. The partial atomic charges of NPACT compounds were evaluated using the Gasteiger-Marsili method [31]. Duplicated compounds with identical InChIKey were stripped out [32]. The number of duplicates was 63 compounds. The prepared files of the NPACT database are available at www.compchem.net/ccdb.

### Molecular docking

**Fig. 1:**
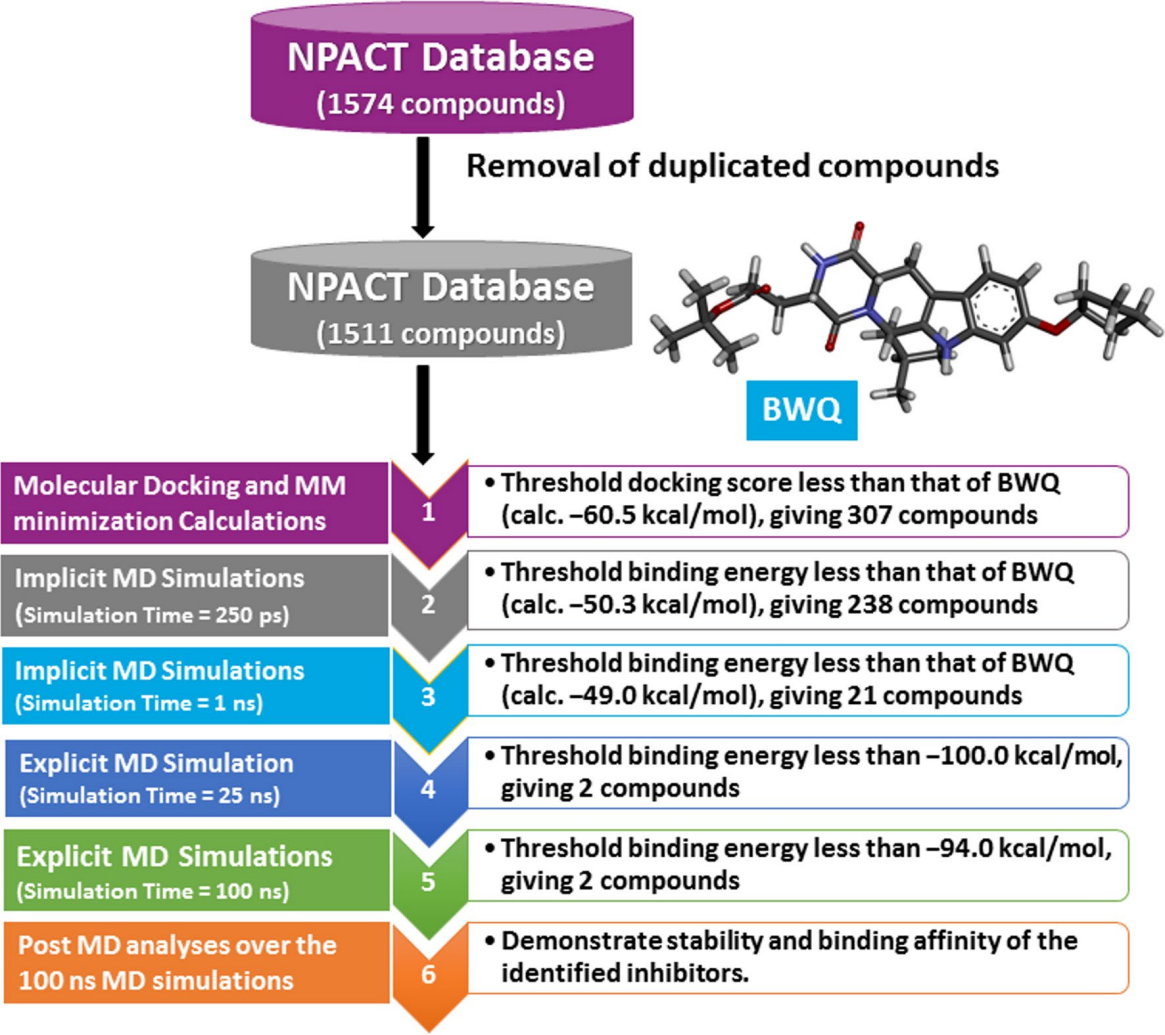
A workflow diagram of the employed computational approaches in addition to the filtration process

All molecular docking calculations were conducted using AutoDock Vina software [33]. MGTools1.5.6 was used to convert the ABCG2 transporter structure into pdbqt format on the basis of AutoDock protocol [34, 35]. THR435 and ASN436, substantial amino acid residues in the active sites of chains A and B, were presented as conformationally flexible residues; however, all other residues were treated as rigid parts. The search exhaustiveness number was adjusted to 200. Other AutoDock Vina parameters were preserved to their default values. The docking grid box dimension was set to 30 Å × 30 Å × 30 Å along *x*-, *y*- and *z*-axes, respectively. The grid spacing of 1.0 Å was utilized. The grid center coordinates were 130.869, 126.675, 145.206 (XYZ assignments, respectively). A workflow diagram of the employed computational approaches and the filtration process of the NPACT database is illustrated in Fig. 1.

### Molecular minimization calculations

All docked inhibitor-ABCG2 complexes were then minimized to an RMSD of 10^−9^ Å with the assistance of Sander code implemented inside AMBER16 software [36]. In molecular mechanical (MM) minimization, the Truncated Newton linear Conjugate Gradient method with optional LBFGS pre-conditioning (LBFGS-TNCG) algorithm [37] was applied in an implicit-solvent utilizing a generalized Born solvent model (igb = 1 [38]). General AMBER force field (GAFF2) [39] was employed for the parameter determination of the investigated inhibitors. At the same time, AMBER force field 14SB [40] was utilized to describe the ABCG2 transporter. The atomic partial charges for the studied inhibitors were assigned using the Austin model with bond and charge correction (AM1-BCC) method [41]. No periodic boundary conditions (PBC) were adopted. The nonbonded cutoff was set to 999 Å.

### Molecular dynamics simulations

Structural and energetical stabilities of potent NPACT compounds in complex with the ABCG2 transporter were inspected by conducting molecular dynamics (MD) simulations using AMBER16 software [36]. In this study, implicit-solvent and explicit-solvent MD simulations were carried out.

In the implicit-solvent MD simulations, the AM1-BCC charge model [41] was utilized to determine the atomic partial charges of the investigated inhibitors. No cutoff and no periodic boundary conditions were executed for nonbonded interactions. The implicit-solvent model with igb = 1 was used to mimic the aqueous solvent effect [38]. The NPACT-ABCG2 complexes underwent energy minimization up to 500 steps using combined steepest and conjugate gradient methods. After that, each minimized system was progressively heated to 300 K throughout 10 ps. The studied complexes were constrained with a force constant of 10 kcal mol^−1^ Å^−2^. Equilibration was carried out over 50 ps in the canonical ensemble (NVT) with the help of the Langevin thermostat. Ultimately, 250, 1000, and 5000 ps production runs were executed. Additionally, snapshots were recorded each 1 ps.

In explicit-solvent MD simulations, the charges of the identified NPACT compounds were assigned utilizing the restrained electrostatic potential (RESP) approach at the HF/6-31G* level with the assistance of Gaussian09 software [42, 43]. All NPACT-ABCG2 systems were solvated in a periodic octagonal box involving a TIP3P water model with a minimal distance to the box edge of 12 Å from any solute atom [44]. The systems were then neutralized by adding the appropriate number of Na^+^ or Cl^−^ counterions to reach 0.15 M NaCl salt concentration. Following the system preparation, energy minimizations of 5000 steps were executed through combined steepest and conjugate gradient methods. Thermalization of the minimized complexes from 0 to 300 K was conducted in six stages throughout 50 ps. MD simulations were performed for 1 ns to equilibrate the investigated complexes under the isothermal-isobaric (NPT) conditions. Finally, the production stage was executed for each complex for 25 ns and 100 ns. The long-range electrostatic interactions were processed utilizing the Particle Mesh Ewald (PME) method [45]. The nonbonded cutoff was adjusted to 12 Å [45]. Langevin dynamics methods with a collision frequency of 1.0 ps^−1^ were applied (i.e., gamma_ln = 1.0 and ntt = 3) to preserve the temperature at 298 K [46]. Barostat pressure was controlled at an average 1 atm via isotropic position scaling using the Berendsen barostat [47]. The SHAKE algorithm with a time step of 2 fs was applied to constrain all the bond lengths involving hydrogen atoms [48]. For the post-dynamics analyses and binding energy calculations, trajectory snapshots were extracted each 10 ps interval over the production stage.

All implicit-solvent and explicit-solvent MD simulations were carried out using the GPU version of pmemd (pmemd.cuda) within AMBER16 software.

### MM-GBSA binding energy calculations

The binding free energy calculations of the ABCG2 transporter bound with the most potent NPACT candidates were calculated using the molecular mechanical-generalized Born surface area (MM-GBSA) approach [49]. In this study, the modified generalized Born (GB) model developed by Onufriev and collaborators (igb = 2) was applied to appoint the polar solvation energy [50]. Based on the collected snapshots over the MD course, the MM-GBSA binding energy (Δ*G*_binding_) were evaluated as follows:

$$ \Delta {\text{G}}_{{{\text{binding}}}}  = {\text{G}}_{{{\text{Complex}}}}  - \left( {{\text{G}}_{{{\text{NPACT}}}}  + {\text{G}}_{{{\text{ABCG2}}}} } \right)$$

where

$$G={E}_{\mathrm{vdw}}+{E}_{\mathrm{ele}}+{G}_{\mathrm{GB}}+{G}_{\mathrm{SA}}$$

*E*_vdw_ is the van der Waals energy. *E*_ele_ stands for electrostatic energy. Besides, *G*_GB_, and *G*_SA_ refer to the general Born solvation and surface area energies, respectively. The configurational entropy (S) is typically neglected due to the higher computational costs [51, 52].

All in silico calculations, including molecular docking, molecular mechanics (MM) minimization, molecular dynamics (MD) simulations, quantum mechanics (QM) calculations, were performed using a hybrid GPU/CPU cluster (hpc.compchem.net). The 2D and 3D structures of ABCG2-NPACT interactions are generated utilizing BIOVIA DS Visualize 2020 [53].

## Results and discussion

### Validation of in silico protocol

**Fig. 2:**
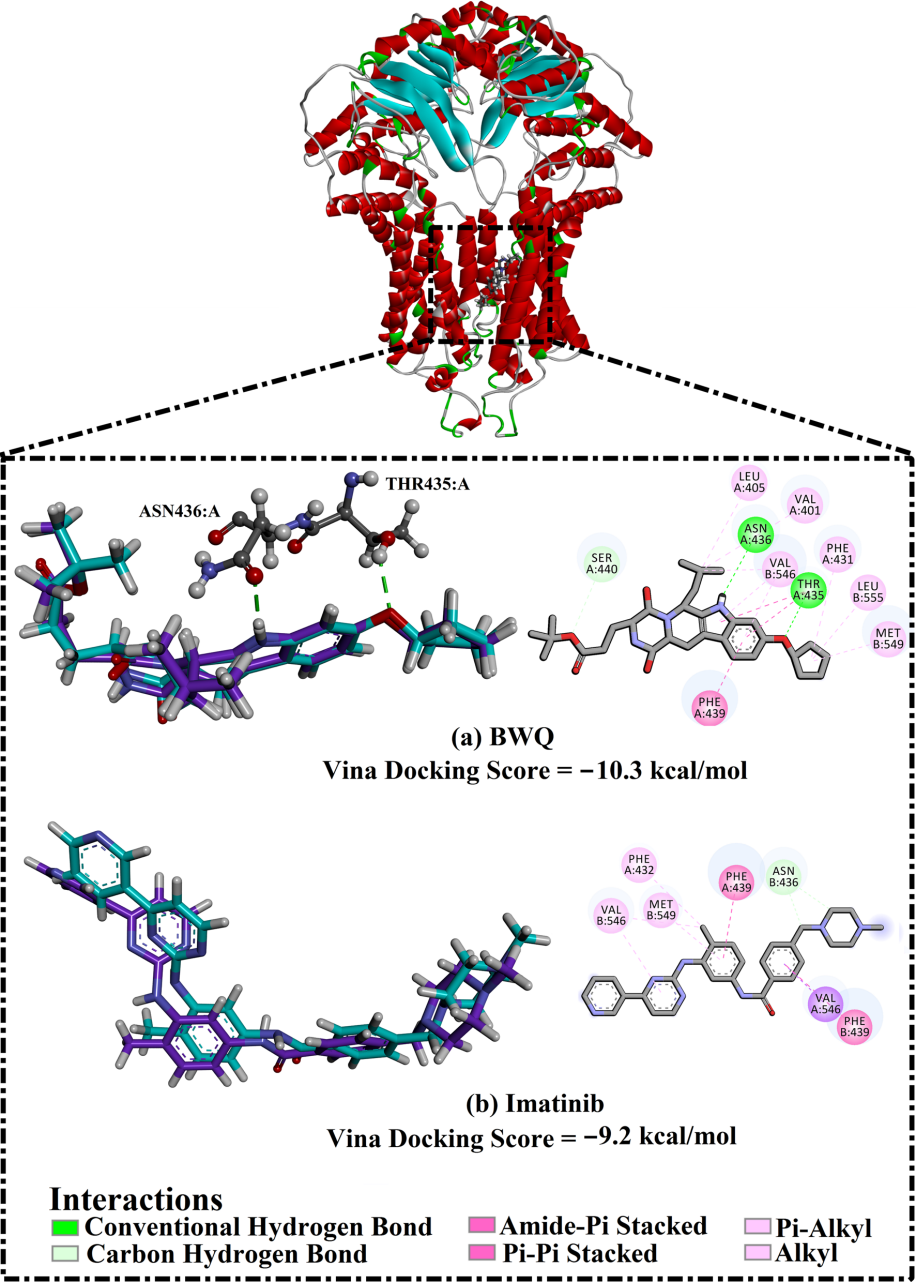
3D and 2D representations of the anticipated docking poses (in mauve) and experimental structures (in cyan) of **a** BWQ and **b** imatinib complexed with ABCG2 transporter

AutoDock Vina parameters were initially validated based on the accessible experimental data. The co-crystallized BWQ and imatinib ligands were re-docked toward the ABCG2 transporter, and the predicted docking poses were compared to the experimentally resolved structures (PDB ID: 6FFC [12] and 6VXH [15], respectively) (Fig. 2). From the data presented in Fig. 2, it is apparent that the anticipated docking poses were similar to the binding modes of the crystal structures. Additionally, the predicted binding modes of BWQ and imatinib manifested 0.22 and 0.38 Å RMSD with respect to their co-crystallized conformation (Fig. 2). Comparing the re-docked structures with their co-crystallized conformations revealed that AutoDock Vina software minutely foretold the correct binding poses of BWQ and imatinib within the binding pocket of the ABCG2 transporter.

According to the predicted docking scores, BWQ and imatinib showed good binding affinities with docking scores of − 10.3 and − 9.2 kcal/mol, respectively. The good potency of BWQ against the ABCG2 transporter may be returned to the ability of NH of indoline ring to exhibit a fundamental hydrogen bond with the carbonyl group of ASN436:A with a bond length of 2.16 Å (Fig. 2). Additionally, the oxygen atom of cyclopentyloxy benzene forms a substantial hydrogen bond with the hydroxyl group of THR435:A with a bond length of 2.14 Å (Fig. 2).

In contrast, imatinib was not capable of exhibiting any hydrogen bonds with the proximal amino acids within the binding site of the ABCG2 transporter (Fig. 2). However, the good docking score of imatinib may be attributed to other interactions such as van der Waals and hydrophobic interactions with the binding pocket’s amino acid residues (Fig. 2). Together these results provide important insights into the use of BWQ over imatinib as a reference inhibitor in the subsequent calculations.

### Virtual screening of NPACT database

Historically, natural products (NPs) are a source of potential drugs, particularly for cancer and infectious diseases [54, 55]. Naturally Occurring Plant-based Anticancer Compound-Activity-Target (NPACT) database focuses on anticancerous natural molecules derived from plants [26]. NPACT is unparalleled in supplying bioactivities of these natural molecules toward various cancer cell lines. The NPACT database includes 19 different classes as illustrated in Fig. S1; terpenoids represents the majority of NPACT compounds (33.0%), followed by flavonoids (21.0%), alkaloids (7.0%), lignans (6.0%), polyketides (6.0%), and simple aromatic natural products (5.0%) (Fig. S1).

To identify potent molecules from a natural source to combat multidrug resistance (MDR), the validated AutoDock Vina protocol was applied to virtually screen the NPACT database against the ABCG2 transporter. The calculated docking scores for all NPACT compounds toward the ABCG2 transporter are summarized in Table S1. As shown in Table S1, there is a wide range of predicted binding affinities of NPACT compounds with docking scores ranging from −3.6 to −12.0 kcal/mol.

Since the reliability of inhibitor-receptor binding affinities using molecular docking technique has been questioned, molecular mechanics (MM) minimizations of ligand-protein in an implicit-solvent, pursued by MM-GBSA binding energy calculations, can anticipate more reliable binding affinities. Therefore, all NPACT compounds in complex with ABCG2 transporter were energetically minimized with the help of AMBER force field. Based on the minimized complexes, the corresponding MM-GBSA//MM binding energies were computed (Table S1). According to the estimated MM-GBSA//MM binding energies, a total of 307 NPACT compounds demonstrated MM-GBSA binding energies lower than BWQ (Δ*G*_binding_ = –60.5 kcal/mol). Among the identified potent NPACT compounds, the most potent compounds were polyketides (27.7%). Besides, potent compounds were also observed to belong to terpenoids, saponin, and flavonoids with respective percentage compound counts of 25.7, 17.6, and 11.7%, respectively (Table S1).

**Fig. 3:**
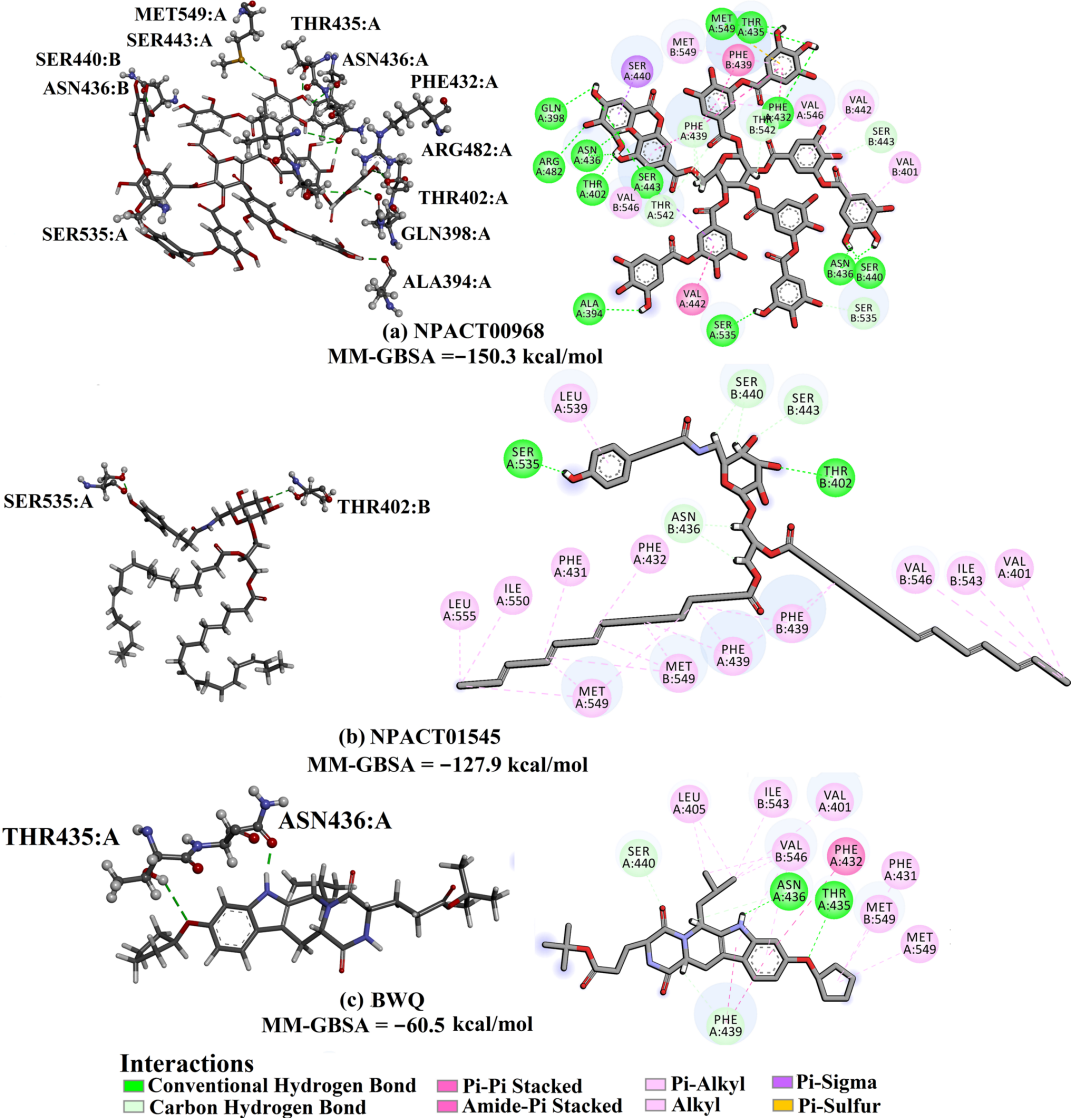
3D and 2D representations of AMBER-based minimized structures, in addition to the evaluated MM-GBSA//MM binding affinities, of the top two potent compounds and BWQ bound with the ABCG2 transporter

The 2D representations for the molecular mechanical-minimized complexes of the top potent twenty-one NPACT compounds are presented in Fig. S2. It is worth noting that those twenty-one potent NPACT compounds were selected according to further energetic calculations in the latter sections. According to the data presented in Fig. S2, all top potent NPACT compounds demonstrated similar binding modes, exhibiting hydrogen bonds with the proximal amino acids, namely THR435, ASN436, and PHE439. Further pi-based, van der Waals and hydrophobic interactions were also noticed between the identified inhibitors and the ABCG2 transporter. Additionally, the 3D and 2D representations for the top two potent inhibitors and BWQ in complex with ABCG2 transporter are depicted in Fig. 3. Besides, the superimposition of the docked structures of the two inhibitors with BWQ is shown in Fig. S3.

Compound NPACT00968 revealed an outstanding binding affinity against the ABCG2 transporter with MM-GBSA//MM binding energy of −150.3 kcal/mol (Table 1). The surpass potentiality of NPACT00968 as an ABCG2 inhibitor may be returned to its capability of forming various hydrogen bonds, hydrophobic, van der Waals interactions, in addition to pi-based interactions with the proximal amino acids within the binding site of ABCG2 transporter (Fig. 3). More precisely, structural insights into the binding mode of the NPACT00968 within the ABCG2 transporter unveiled that the hydroxyl groups of pyrocatechol rings exhibit fifteen hydrogen bonds with the backbone of the ALA394:A, GLN398:A, THR402:A, PHE432:A, THR435:A, ASN436:A, ASN436:B, SER440:B, SER443:A, ARG482:A, SER535:A, and MET549:A with bond lengths ranging from 2.28 to 2.98 Å (Fig. 3).

**Table 1:**
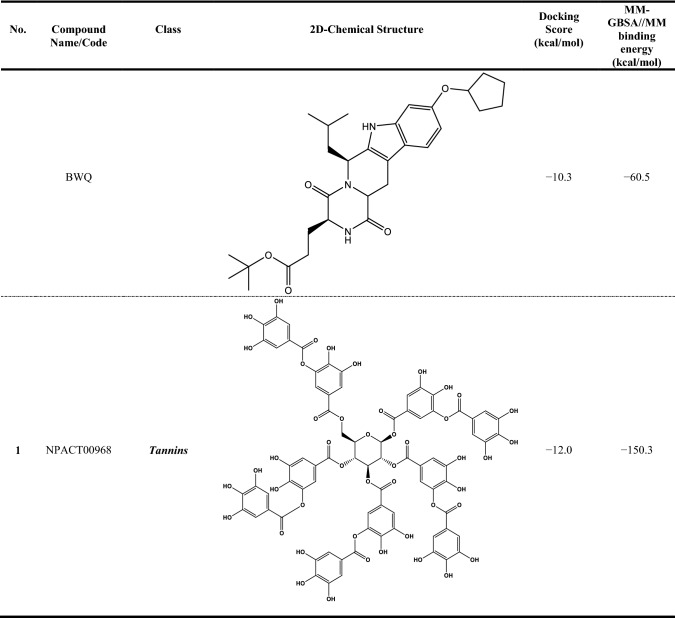

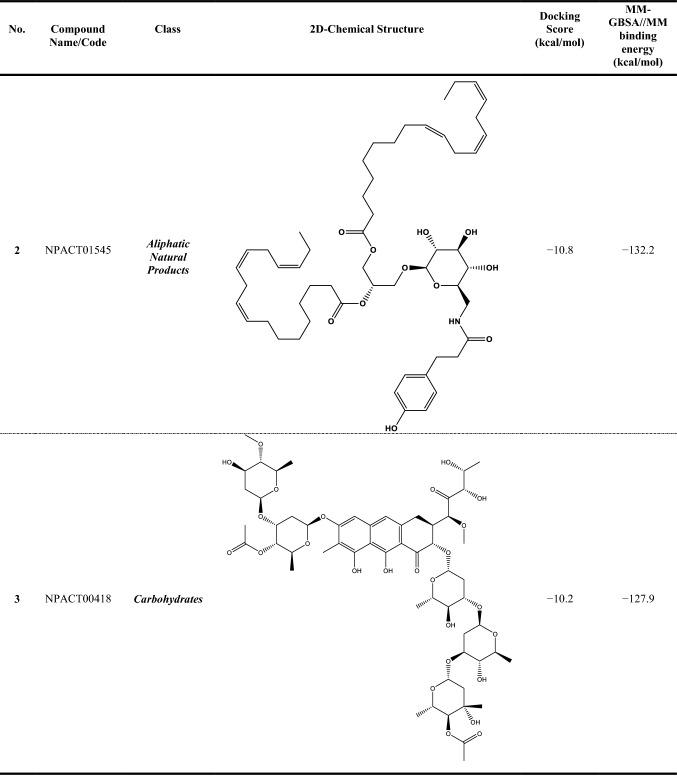

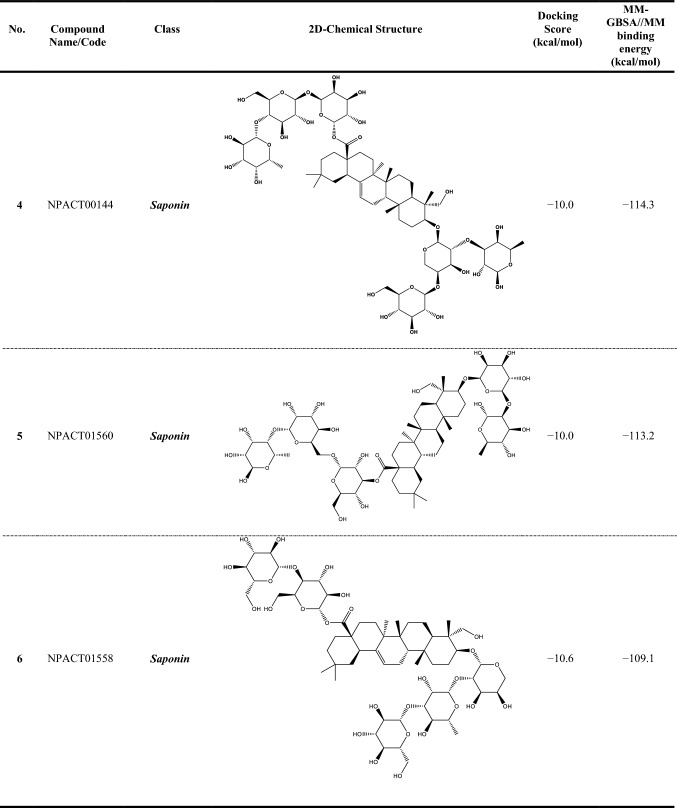

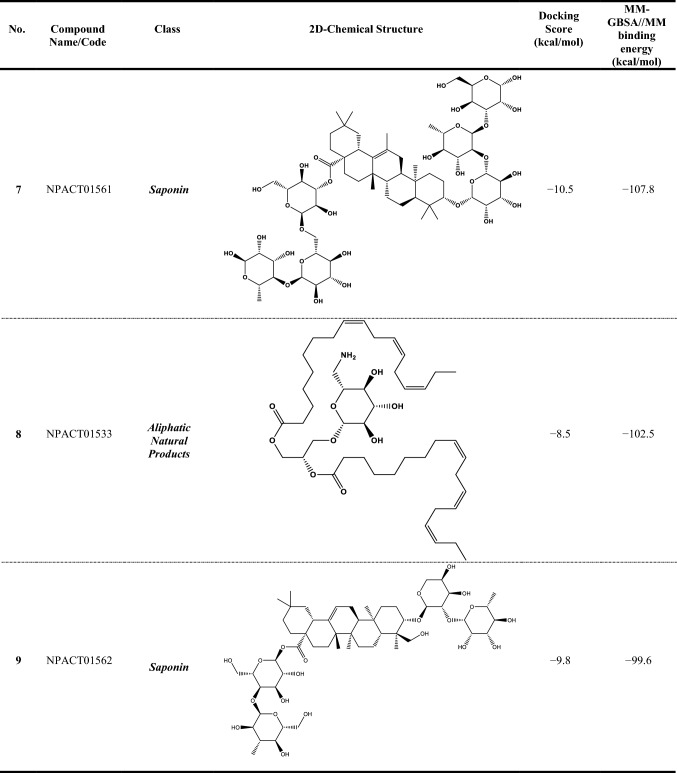

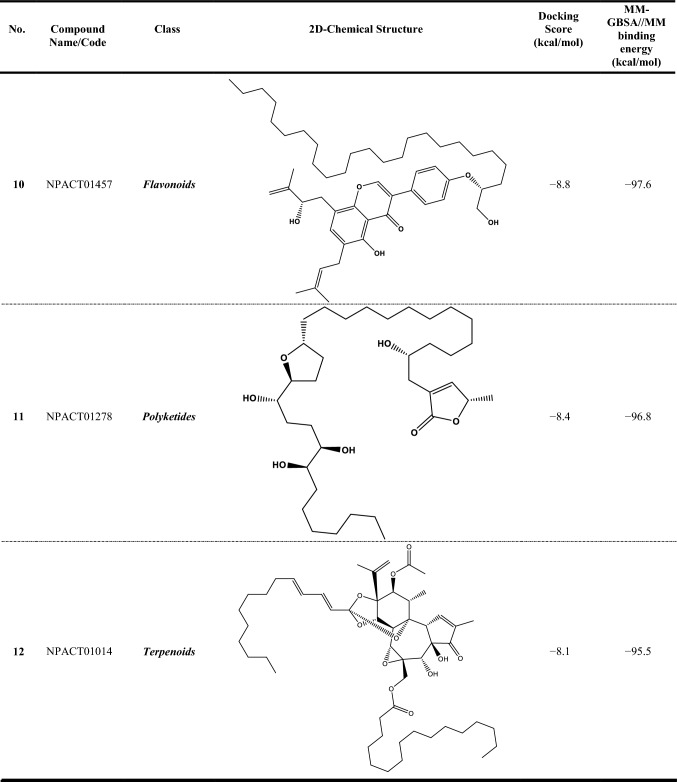

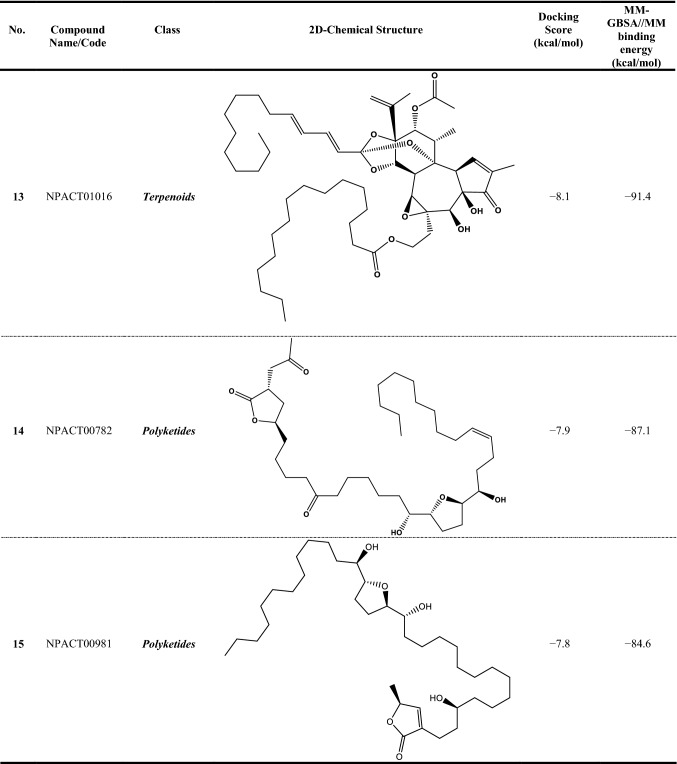

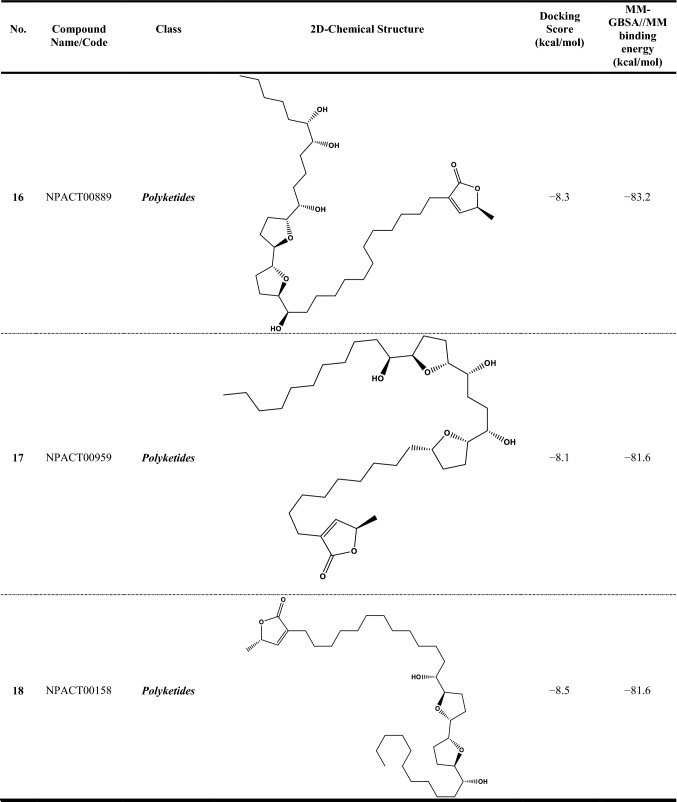

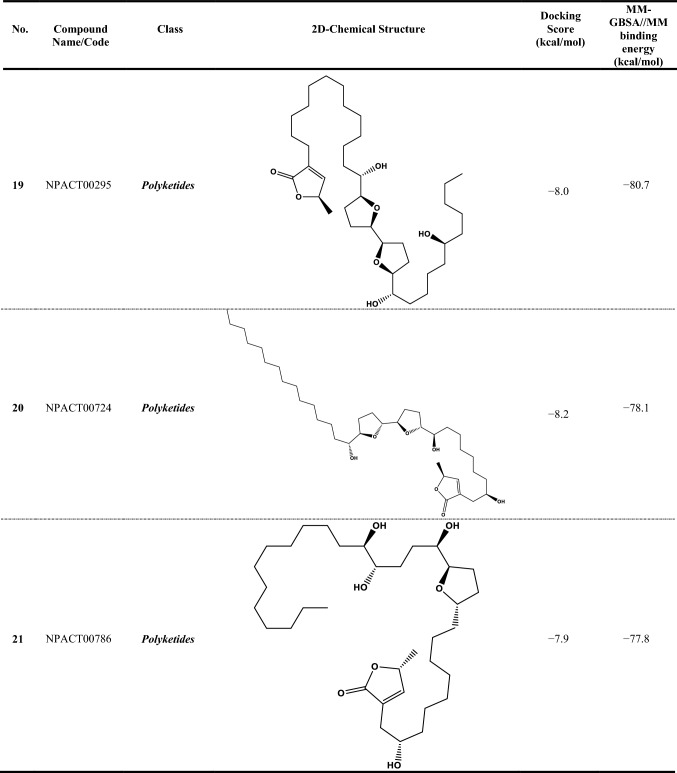
Evaluated docking score, classification category, 2D chemical structure, and MM-GBSA//MM binding energy for BWQ and the top potent NPACT compounds against ABCG2 homodimer

^a^Data ranked based on the estimated MM-GBSA//MM binding energy

Compound NPACT01545 demonstrated the second-highest binding affinity against the ABCG2 transporter with MM-GBSA//MM binding energy of −127.9 kcal/mol (Table 1). Similarly, hydroxyl groups of cyclohexan-1,2-diol and phenol ring form two hydrogen bonds with the backbone hydroxyl groups of THR402:B and SER535:A with bond lengths of 2.50 and 2.37 Å, respectively (Fig. 3).

Compared to the two novel pinpointed inhibitors, BWQ manifested a satisfactory MM-GBSA//MM binding energy of −60.5 kcal/mol toward the ABCG2 transporter (Table 1).

### Molecular dynamics simulations

**Fig. 4:**
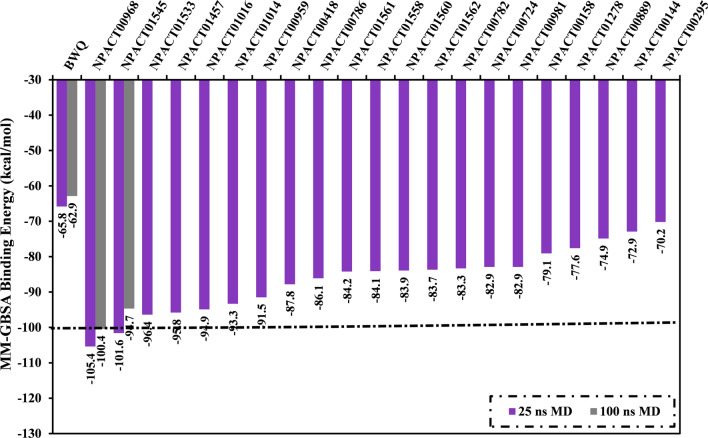
Calculated MM-GBSA binding energies for BWQ inhibitor and the top potent NPACT compounds complexed with ABCG2 transporter throughout 25 ns and 100 ns MD simulations

Molecular dynamics (MD) simulations examine the steadiness of receptor-inhibitor complexes, structural specifics conformational elasticities, as well as further confidence of receptor-inhibitor affinities [56, 57]. As a result, the most potent identified NPACT compounds (with MM-GBSA//MM <  −60.5 kcal/mol) complexed with ABCG2 transporter were submitted to MD simulations. To diminish the in silico cost and time, the MD simulations were conducted in an implicit water solvent for 250 ps. Besides, the MM-GBSA approach was applied to estimate the corresponding binding energies. The corresponding MM-GBSA binding energies for the opted NPACT compounds are summarized in Table S2. What is interesting about the data in Table S2 is that 238 compounds (i.e., approximately three-fourths of the filtered NPACT compounds) demonstrated lower binding energies (Δ*G*_binding_) than that of BWQ (calc. –50.3 kcal/mol). To realize a higher degree of thoroughness, MD simulations of 238 NPACT compounds bound with ABCG2 transporter were then subjected to 1 ns MD simulations in an implicit water solvent. The corresponding MM-GBSA binding affinities were estimated (Table S3). As shown in Table S3, twenty-one compounds showed lower binding energies (Δ*G*_binding_) than that of BWQ (calc. –49.0 kcal/mol). These twenty-one compounds were further subjected to the 25 ns MD simulations in an explicit water solvent to gain more reliable binding energies against the ABCG2 transporter. The corresponding MM-GBSA binding energies were evaluated (Fig. 4). It can be seen from the data in Fig. 4 that two compounds, namely NPACT00968 and NPACT01545, revealed promising binding energies for ABCG2 transporter with Δ*G*_binding_ <  −100.0 kcal/mol (Fig. 4). To boost the trustworthiness of the noticed finding, MD simulations were protracted to 100 ns, and the corresponding binding energies were estimated (Fig. 4).

From the data in Fig. 4, it is apparent that there was no cognizable difference between the evaluated MM-GBSA binding affinity for NPACT00968 and NPACT01545 bound with ABCG2 transporter over 25 ns and 100 ns MD simulations. Compared to the MM-GBSA binding energy of BWQ (Δ*G*_binding_ =  −62.9 kcal/mol), NPACT00968 and NPACT01545 demonstrated better binding affinities over the 100 ns MD simulations toward ABCG2 transporter with Δ*G*_binding_ of −100.4 and −94.7 kcal/mol, respectively.

**Fig. 5:**
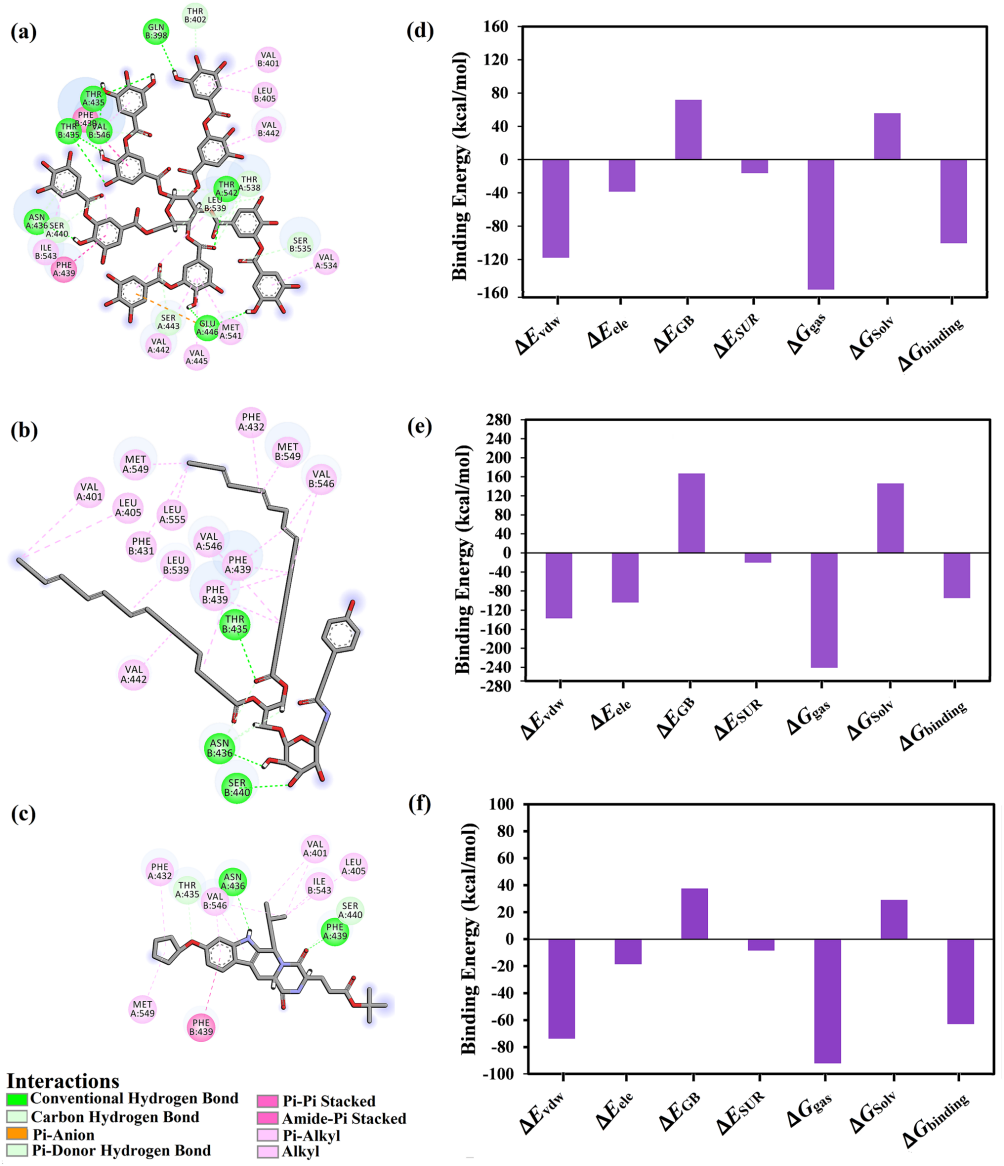
2D representations of binding modes of **a** NPACT00968, **b** NPACT01545, and **c** BWQ bound with ABCG2 transporter according to the average structure throughout the 100 ns MD simulations, as well as components of the MM-GBSA binding energies for **d** NPACT00968, **e** NPACT01545 and **f** BWQ in complex with ABCG2's active site over the MD course of 100 ns

The average structures for NPACT00968, NPACT01545, and BWQ within the binding site over the 100 ns MD simulations are presented in Fig. 5. The most interesting finding was that NPACT00968 and NPACT01545 conserved nine and three hydrogen bonds, respectively, with the key amino acids of ABCG2 transporter over the 100 ns MD simulations (Fig. 5). BWQ ditto displayed an adequate binding affinity throughout 100 ns MD simulations against ABCG2 transporter with an average Δ*G*_binding_ of −62.9 kcal/mol, forming only two hydrogen bonds with the proximal amino acid residues of ABCG2 transporter (Fig. 5). In an epilogue, MD simulations combined with MM-GBSA binding energy calculations demonstrated outstanding binding affinities of NPACT00968 and NPACT01545 against the ABCG2 transporter. By means of molecular dynamics simulations, a far-reaching characterization of the ABCG2 homodimer and prediction of novel stable inhibitors were obtained.

Decomposition of average MM-GBSA binding energy during 100 ns MD simulations was also executed to reveal the nature of prime interactions in the NPACT00968-, NPACT01545-, and BWQ-ABCG2 complexes (Fig. 5). *E*_vdw_ was a considerable contributor to the NPACT00968-, NPACT01545-, and BWQ-ABCG2 binding affinities with average values of –117.8, –137.2, and –73.6 kcal/mol, respectively. Besides, electrostatic interactions (*E*_ele_) demonstrated a favorable contribution with average values of –103.5, –38.4, and –18.4 kcal/mol for NPACT00968, NPACT01545, and BWQ, respectively. It is also worth noting that the *E*_ele_ energies of NPACT00968 and NPACT01545 were approximately five- and two-folds stronger than that of BWQ, respectively. Together these findings provide quantitative data of the binding affinities of NPACT00968 and NPACT01545 as prospective ABCG2 drug candidates.

**Fig. 6:**
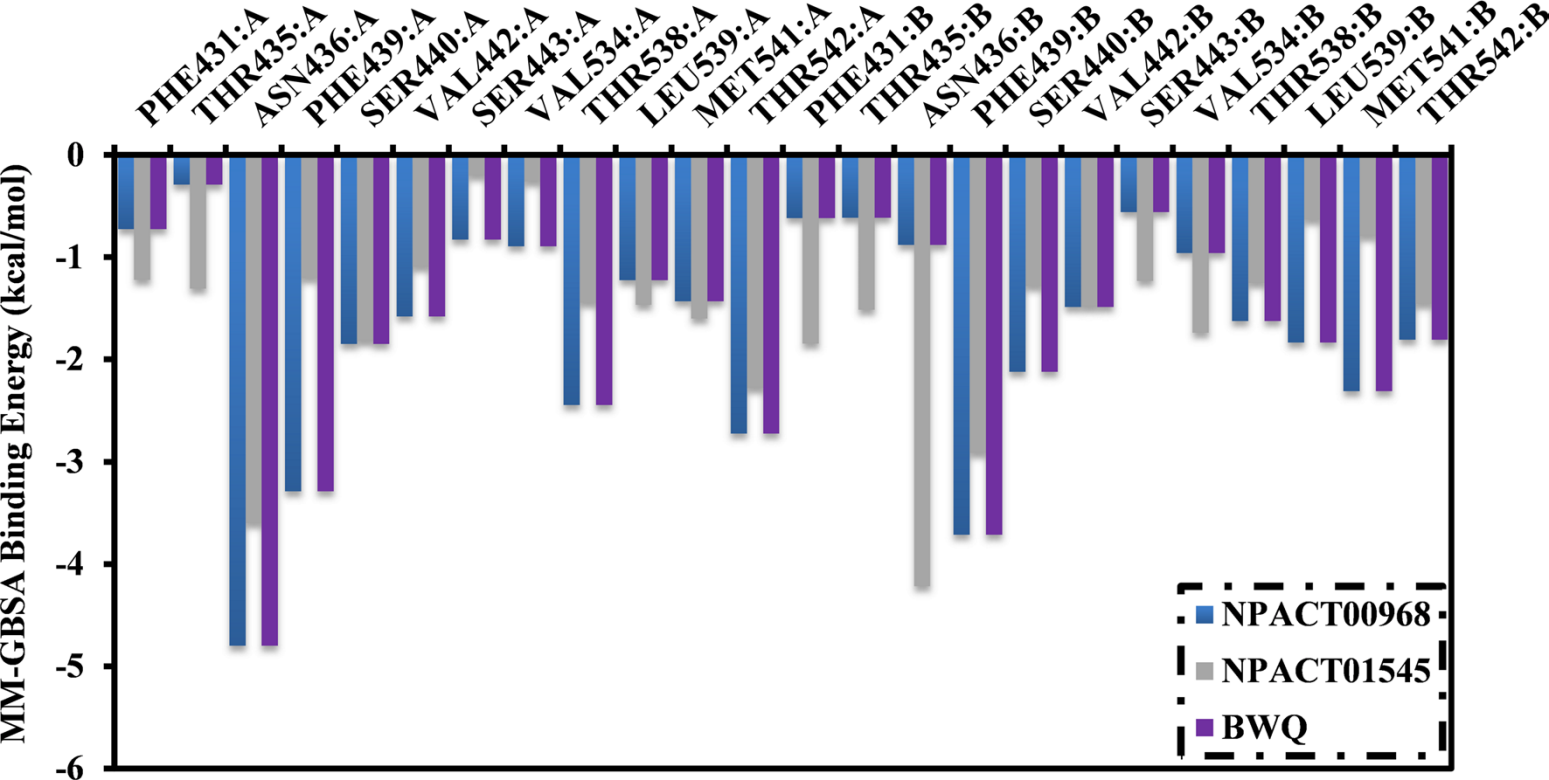
Per-residue decomposition of the total binding energy (kcal/mol) of NPACT00968, NPACT01545 and BWQ bound with ABCG2 transporter

To further pinpoint the key amino acids in charge of stabilizing NPACT00968, NPACT01545, and BWQ inside the binding pocket of the ABCG2 transporter, the estimated ∆*G*_binding_ values were dissociated into the individual residue contributions. The amino acid residues with binding energy participation less than −0.50 kcal/mol were considered and are depicted in Fig. 6. All investigated inhibitors demonstrated tangible contact with most of the ABCG2 active site residues, including THR435, ASN436, PHE439, and SER440 (Fig. 6). Furthermore, there was massive participation by ASN436 toward the total binding free energy with values of −4.8, −3.6, and −2.3 kcal/mol for NPACT00968-, NPACT01545-, and BWQ-ABCG2 complexes, respectively.

### Post-dynamics analyses

To further confirm scrutinizing the constancy and behavior of the NPACT00968 and NPACT01545 bound with the ABCG2 transporter, both structural and energetical analyses were accomplished over the MD simulation time of 100 ns and compared to those of BWQ. Four characteristics were estimated, including center-of-mass (CoM), root-mean-square deviation (RMSD), hydrogen bond length, and binding energy per frame.

#### Hydrogen bond analysis

It has been documented that the hydrogen bonds (HB) exhibited at the binding site of the protein between key amino acid residues and ligand could play a pivotal role in the high binding affinity of the studied ligand with a protein [58]. Consequently, HB analysis was executed for NPACT00968, NPACT01545, and BWQ complexed with ABCG2 transporter during 100 ns MD simulations (Table 2). As shown in Table 2, the three investigated inhibitors exhibited a stationary hydrogen bond with ASN436:A with HB total occupancy values of 93.2, 91.1 and 87.9% for NPACT00968, NPACT01545, and BWQ complexes, respectively. The high HB occupancy emphasizes the prominent role of ASN436:A inside the binding pocket of the ABCG2 transporter. Comparing the results listed in Table 2 revealed greater stability of NPACT00968 compared to NPACT01545 and BWQ. Specifically, NPACT00968 formed three stable hydrogen bonds with THR435:A, ASN436:A, and PHE439:A with an average HB distance of 2.8, 2.6, and 2.7 Å, respectively. Similarly, NPACT01545 and BWQ hydrogen bond was noticed with ASN436:A with average HB distances of 2.8 and 2.9 Å, respectively. Additionally, both NPACT01545 and BWQ executed a moderate stable hydrogen bond with SER440:A, and PHE439:A with an average value of 2.9 and 2.7 Å with HB occupancy of 55.6 and 51.8%, respectively.

**Table 2:** Hydrogen bonds exhibited between the investigated inhibitors and the proximal amino acids within the binding pocket of the ABCG2 transporter

Compound name/code	Acceptor	Donor	Distance (Å)^a^	Angle (degree)^a^	Occupied (%)^b^
BWQ	BWQ@O	PHE439:A@O–H	2.7	153	51.8
	ASN436:A@O	BWQ @N–H	2.9	163	87.9
NPACT00968	THR435:A@O	NPACT00968@O–H	2.8	149	61.7
	ASN436:A@O	NPACT00968@O–H	2.6	158	93.2
	PHE439:A@O	NPACT00968@O–H	2.7	165	78.5
NPACT01545	NPACT01545@O	SER440:A@O–H	2.9	162	55.6
	ASN436:A@O	NPACT01545@O–H	2.8	154	91.1

^a^The hydrogen bonds are scrutinized by the donor–acceptor atom length less than 3.5 Å. Additionally, acceptor-H-donor angle is higher than 120°

^b^Only hydrogen bonds with occupancy greater than 50% were observed

#### Binding energy per frame

**Fig. 7:**
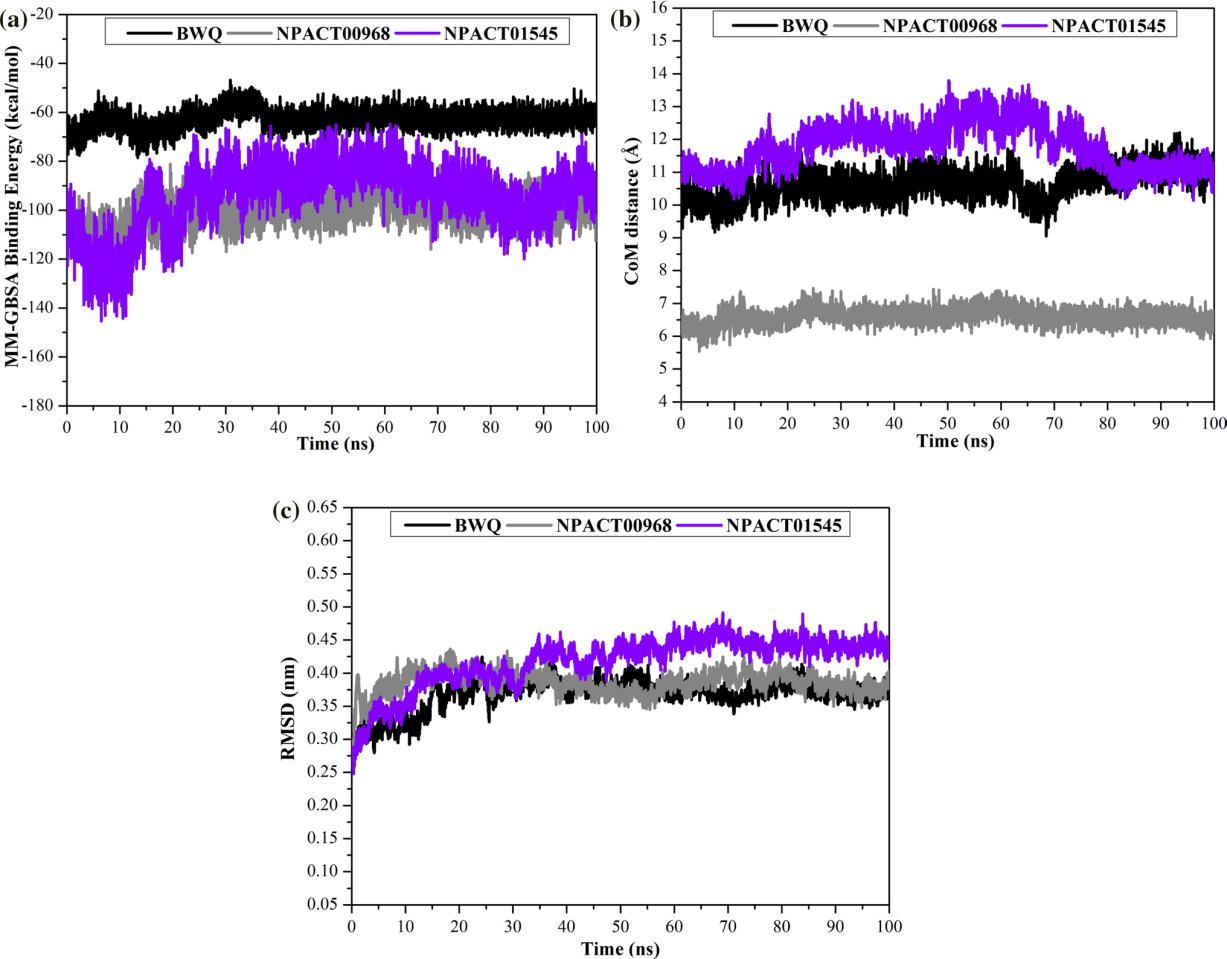
**a** Evaluated MM-GBSA binding energy per frame, **b** center-of-mass (CoM) distances and **c** root-mean-square deviation (RMSD) of the backbone atoms from the starting structure of NPACT00968 (in gray), NPACT01545 (in violet), and BWQ (in black) against the ABCG2 transporter during 100 ns MD simulations

The correlation between binding energy and time was utilized to scrutinize the comprehensive energetic stability of NPACT00968-, NPACT01545-, and BWQ-ABCG2 complexes over the 100 ns MD simulations (Fig. 7a). An exciting portion of this graph is the overall constancy of NPACT00968-, NPACT01545-, and BWQ-ABCG2 with average binding energies (Δ*G*_binding_) of −100.4, −94.7, and −62.9 kcal/mol, respectively. The most interesting finding was that all inspected complexes conserve their stability throughout 100 ns MD simulations.

#### Center-of-mass distance

To obtain a more in-depth insight into the steadiness of inhibitor-ABCG2 complexes over the 100 ns MD simulations, center-of-mass (CoM) distances were investigated between NPACT00968, NPACT01545, and BWQ and ASN436:A (Fig. 7b). The most interesting aspect of this graph is that CoM distances were more narrow-fluctuated for NPACT00968 in complex with ABCG2 transporter than for NPACT01545 and BWQ with average values of 6.6, 11.8, and 10.6 Å, respectively.

#### Root-mean-square deviation

To monitor the influence of the investigated inhibitors on the conformational stability of the ABCG2 transporter throughout 100 ns MD simulations, the root-mean-square deviation (RMSD) values of the backbone atoms were evaluated for the inspected complexes with respect to the starting structures (Fig. 7c). As shown in the plots, RMSD analyses elucidated that the investigated complexes began steadiness after 30 ns and preserved their stabilities till the end of 100 ns MD simulations. The estimated RMSD values for these systems remained below 0.45 nm during the 100 ns MD simulations. Generally, these findings indicated that NPACT00968 and NPACT01545 are tightly bonded and do not impact the comprehensive topology of the ABCG2 homodimer.

### Tannic acid vs. pibrentasvir

In the current study, tannic acid (NPACT00968) showed promising binding affinity against the ABCG2 transporter. NPACT00968 has formerly been reported to conquer neoplasm growth in various kinds of cancer based on in vitro activity, preclinical studies, and observational studies [59]. In an attempt to shine new light on NPACT00968 as a potent ABCG2 inhibitor, the binding affinity of NPACT00968 was compared to pibrentasvir. Pibrentasvir is one of the prospective drug candidates in clinical-trial or investigational stages as ABCG2 inhibitors. Pibrentasvir was proposed as a therapeutic option for multidrug-resistant cancers via targeting ABCG2 transporter based on an in silico drug discovery study [19]. Therefore, the binding affinity of pibrentasvir with ABCG2 transporter was estimated over 100 ns MD simulations and compared to NPACT00968 (Fig. S4). As shown in Fig. S4, the average MM-GBSA binding energies (∆*G*_binding_) for pibrentasvir with ABCG2 transporter was −96.9 kcal/mol, compared to −100.4 kcal/mol for NPACT00968, and were dominated by *E*_vdw_ interactions with average values of −117.8 and −115.5 kcal/mol, respectively (Fig. S4). Notably, the total MM-GBSA binding energies of the NPACT00968 and pibrentasvir were approximately identical. Structural and energetic analyses for NPACT00968- and pibrentasvir-ABCG2 complexes during the 100 ns MD simulations demonstrated that i) there was general stability for NPACT00968- and pibrentasvir-ABCG2 complexes over the MD simulations and ii) both NPACT00968 and pibrentasvir do not affect the comprehensive topology of the ABCG2 transporter (Fig. S4).

## Conclusion

ATP-Binding Cassette (ABC) transporters are included in the efflux of xenobiotic molecules and are in charge of diminishing cumulation of drugs in multidrug-resistant (MDR) cells. ABCG2 is a polyspecific efflux transporter that is a member of the ATP-binding cassette superfamily. ABCG2 has a critical role in tissue protection toward several xenobiotics. Herein, the Naturally Occurring Plant-based Anticancer Compound-Activity-Target (NPACT) database was screened as potential ABCG2 inhibitors utilizing molecular docking, molecular mechanics (MM) minimizations, and molecular dynamics (MD) techniques. Compounds NPACT00968 and NPACT01545 were identified as potential ABCG2 inhibitors according to molecular docking and molecular minimization and MM-GBSA binding energy calculations. The MM-GBSA binding energies throughout 100 ns MD simulations demonstrated up-and-coming binding affinities of NPACT00968 and NPACT01545 against ABCG2 transporter with Δ*G*_binding_ of −100.4 and −94.7 kcal/mol, respectively. The energetic and structural analyses over 100 ns MD simulations confirmed the high stability of identified inhibitors. In vitro and in vivo assays are anticipated to further identify the role of NPACT00968 and NPACT01545 as prospective inhibitors curative for MDR cancer treatment.

## Supplementary Information

Below is the link to the electronic supplementary material.

Supplementary file1 (DOCX 3362 kb)

## Declarations

### Data availability

The data that support the findings of this study are available in the Supporting Information Material of this paper.
