# Supplementary material for: Naturally occurring plant-based anticancerous candidates as prospective ABCG2 inhibitors: an in silico drug discovery study
Source: Mol Divers. 2022 Feb 28;26(6):3255–77. doi: 10.1007/s11030-022-10389-6 (PMC9636125; doi:10.1007/s11030-022-10389-6)
Supplement: Supplementary file 1 — Supplementary file1 (DOCX 3362 kb) [file 11030_2022_10389_MOESM1_ESM.docx]

# Naturally occurring plant-based anticancerous candidates as prospective ABCG2 inhibitors: An *in silico* drug discovery study

Mahmoud A. A. Ibrahim,^1*^ Alaa H. M. Abdelrahman,^1^ Esraa A. A. Badr,^1^ Nahlah Makki Almansour,^2^ Othman R. Alzahrani,^3^ Muhammad Naeem Ahmed,^4^ Mahmoud E. S. Soliman,^5^ Mohamed Ahmed Naeem,^6^ Ahmed M. Shawky,^7^ Peter A. Sidhom,^8^ Gamal A. H. Mekhemer,^1^ and Mohamed A. M. Atia^9^*

*^1^Computational Chemistry Laboratory, Chemistry Department, Faculty of Science, Minia University, Minia 61519, Egypt; m.ibrahim@compchem.net, a.abdelrahman@compchem.net, e.badr@compchem.net, gmekhemer@mu.edu.eg*

*^2^Department of Biology, College of Science, University of Hafr Al Batin, Hafr Al Batin 1803, Saudi Arabia;* *nahlama@uhb.edu.sa*

*^3^Department of Biology, Faculty of Sciences, University of Tabuk, Tabuk 71491, Saudi Arabia; o-alzahrani@ut.edu.sa*

*^4^Department of Chemistry, The University of Azad Jammu and Kashmir, Muzaffarabad 13100, Pakistan; drnaeem@ajku.edu.pk*

*^5^Molecular Modelling and Drug Design Research Group, School of Health Sciences, University of KwaZulu-Natal, Westville, Durban 4000, South Africa; soliman@ukzn.ac.za*

*^6^ Ain Shams University Specialized Hospital, Ain Shams University, Cairo, Egypt;* *naeem70.ash@gmail.com*

*^7^Science and Technology Unit (STU), Umm Al-Qura University, Makkah 21955, Saudi Arabia;* *ahmed_shawkius@hotmail.com*

*^8^Department of Pharmaceutical Chemistry, Faculty of Pharmacy, Tanta University, Tanta 31527, Egypt; peter.ayoub@pharm.tanta.edu.eg*

*^9^Molecular Genetics and Genome Mapping Laboratory, Genome Mapping Department, Agricultural Genetic Engineering Research Institute (AGERI), ARC, Giza, 12619, Egypt;* *matia@ageri.sci.eg*

**
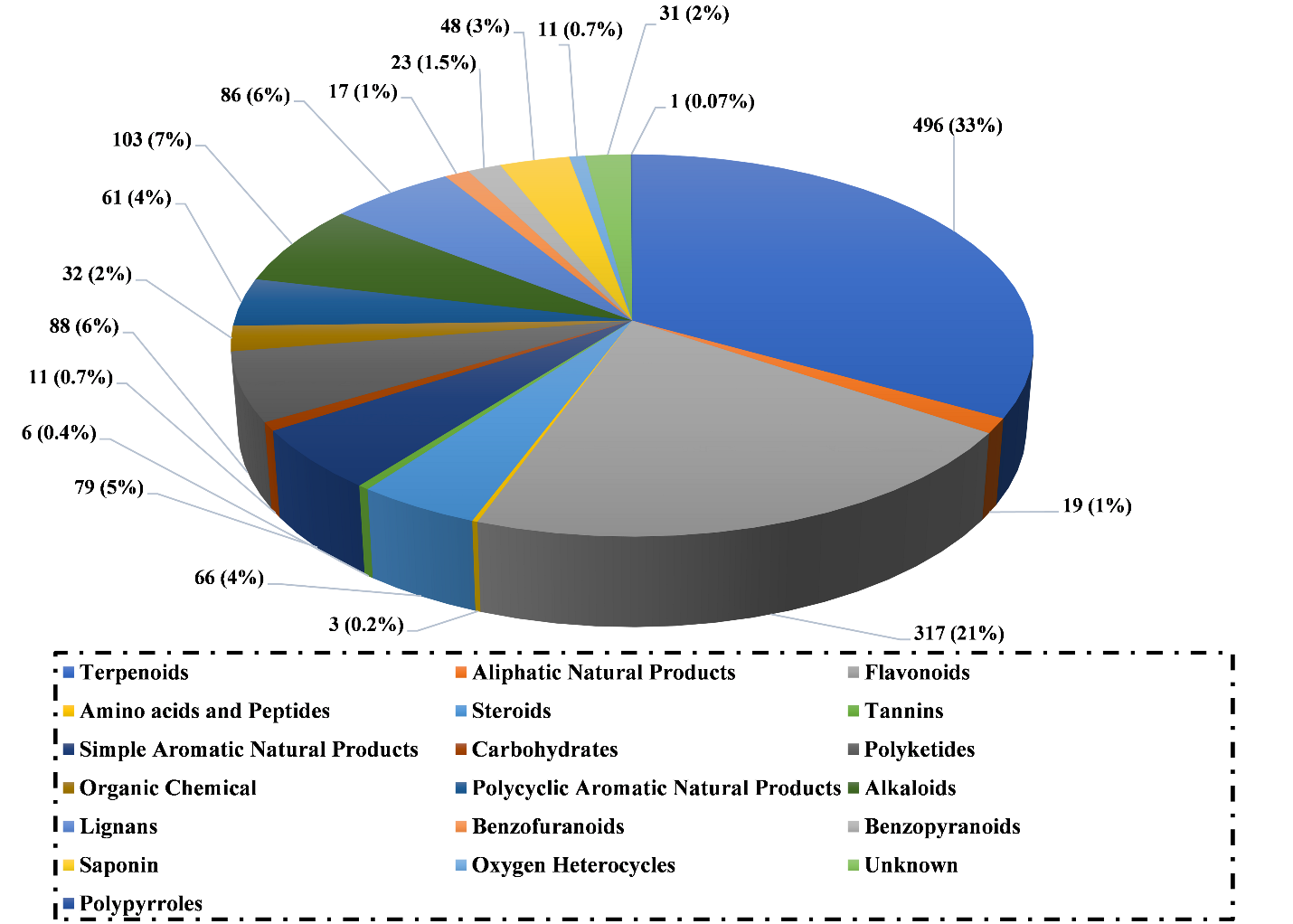
**

**Figure S1**. Pie chart demonstrating the classification of the 1511 natural product compounds.

**
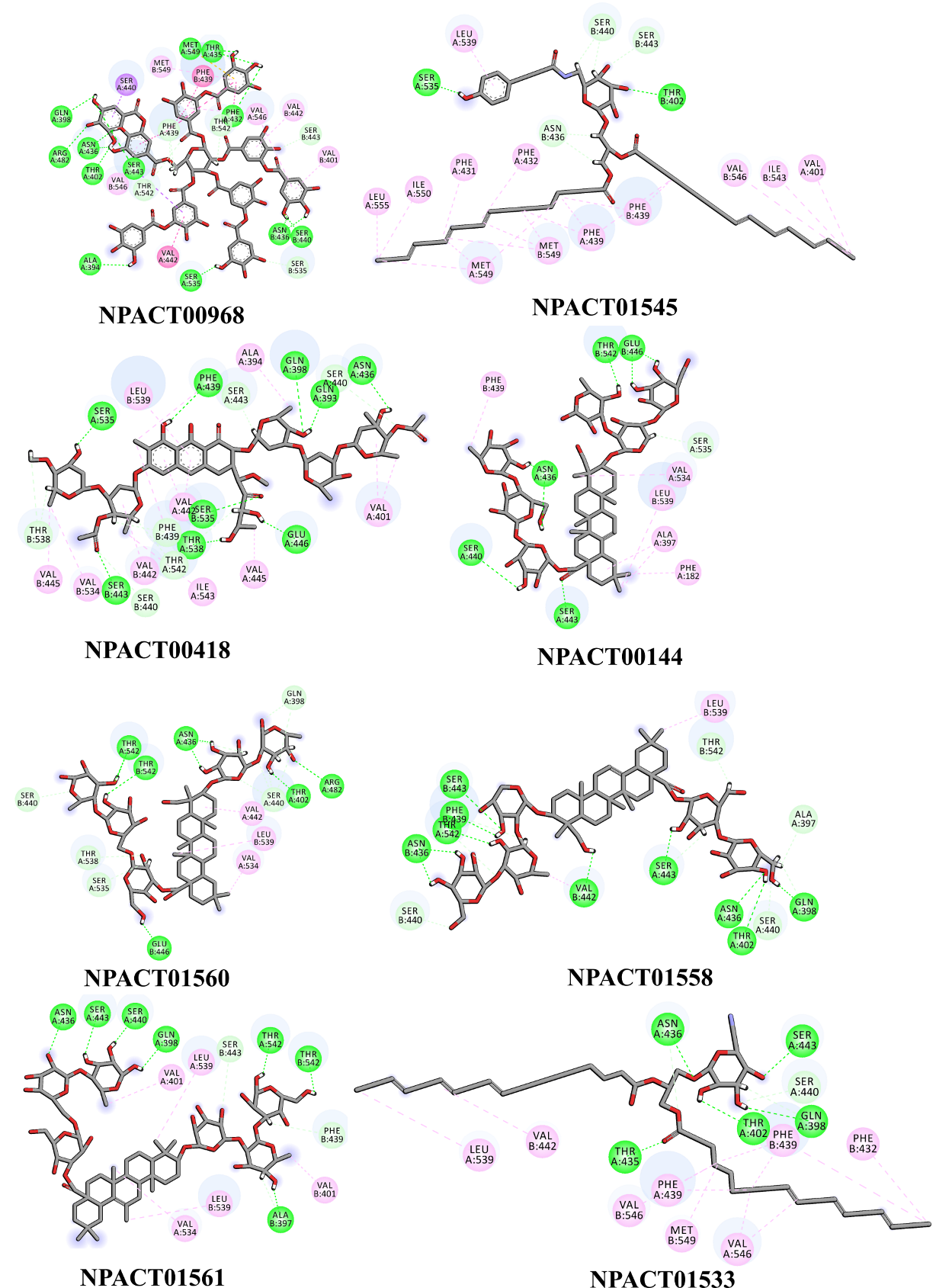
**

**Figure S2.** 2D representations of AMBER-based minimized structures of the twenty-one potent molecules complexed with the ABCG2 transporter.

**
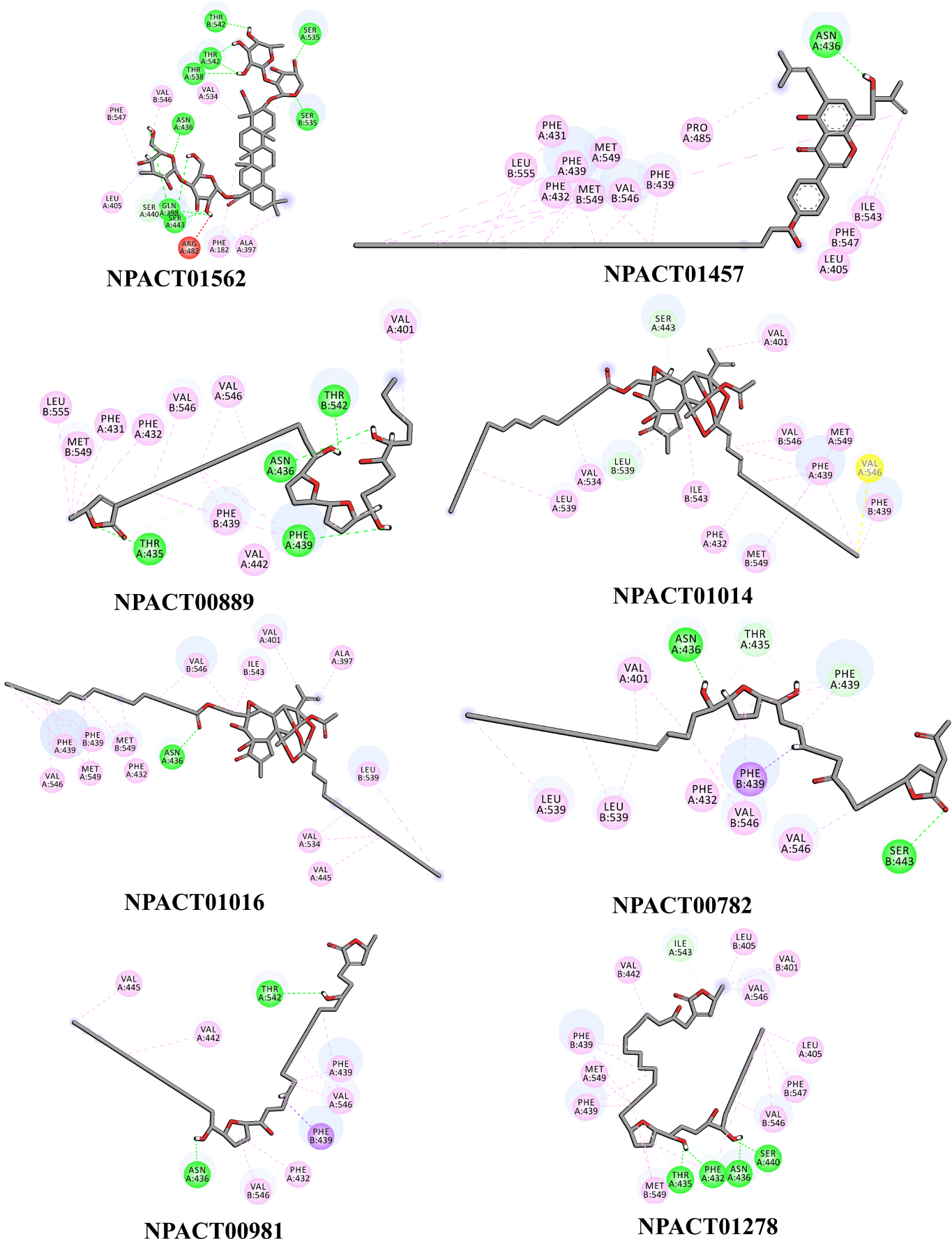
**

**Figure S2.** *Continued.*

**
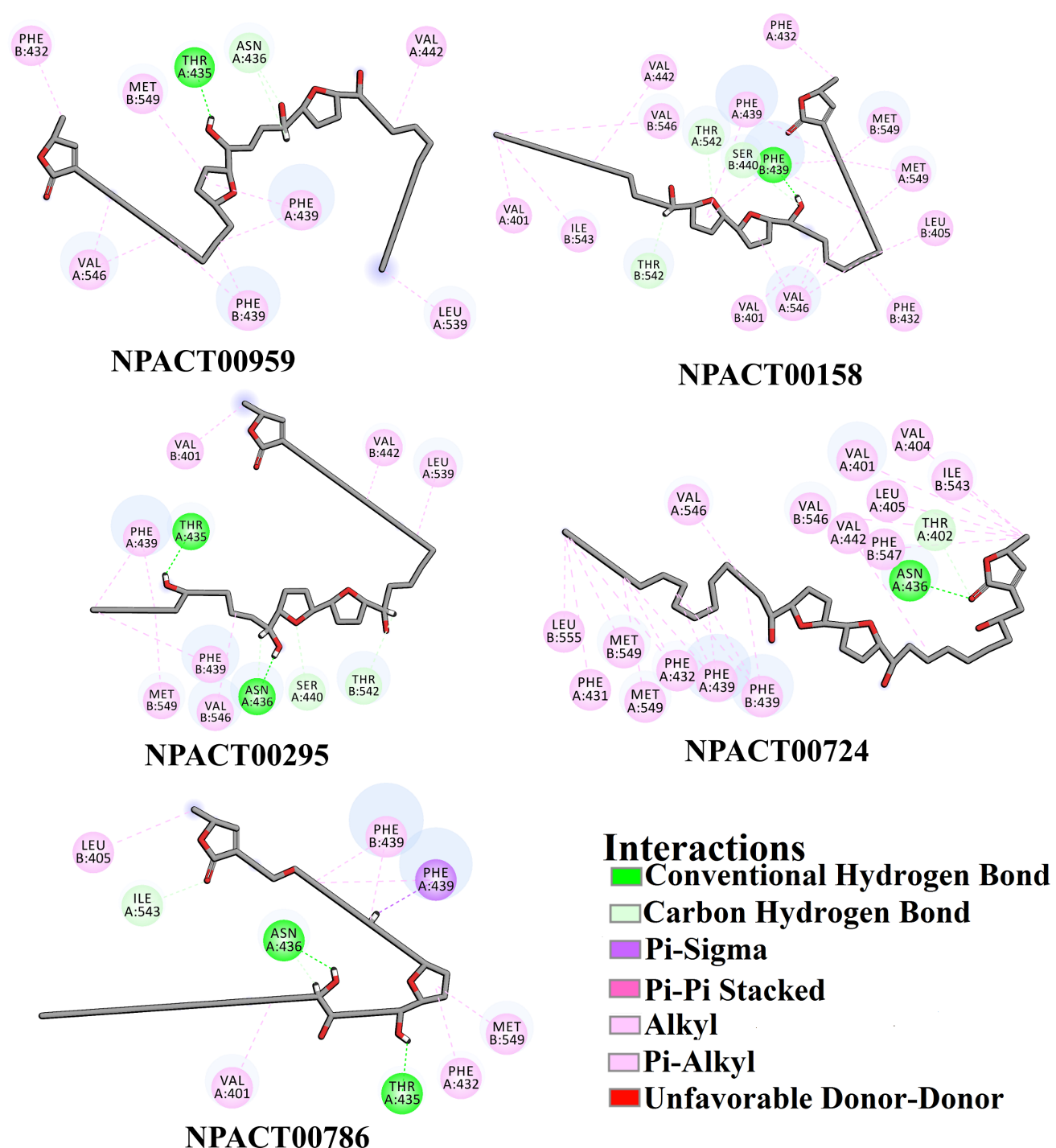
**

**Figure S2.** *Continued.*


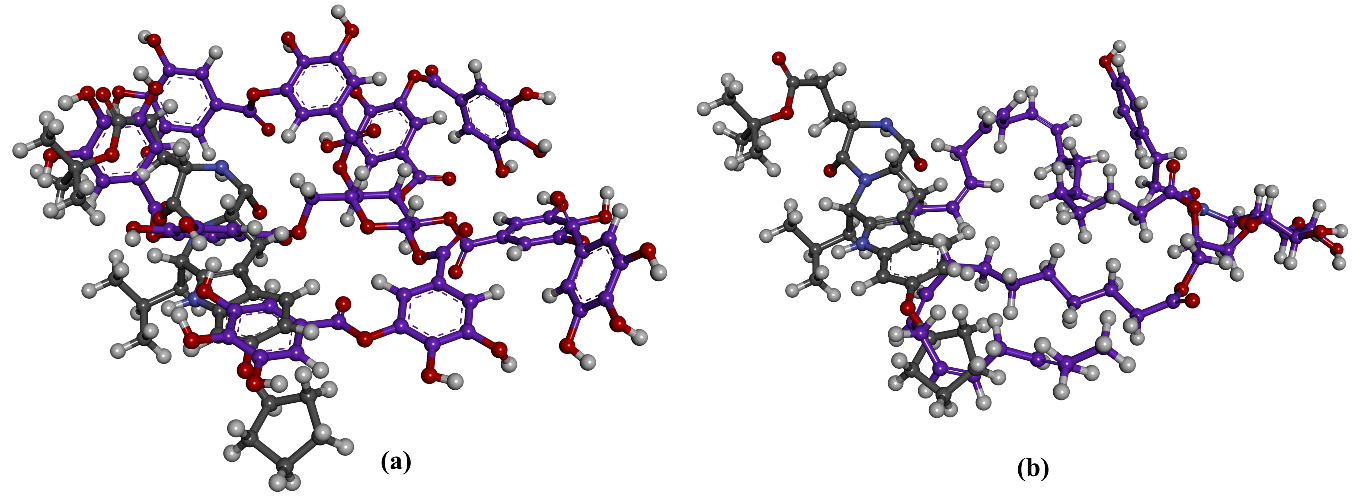


**Figure S3**: Superimposition of the docked structures of (a) NPACT00968 (in mauve) and BWQ (in gray), and (b) NPACT01545 (in mauve) and BWQ (in gray) complexed with the ABCG2 transporter.

###
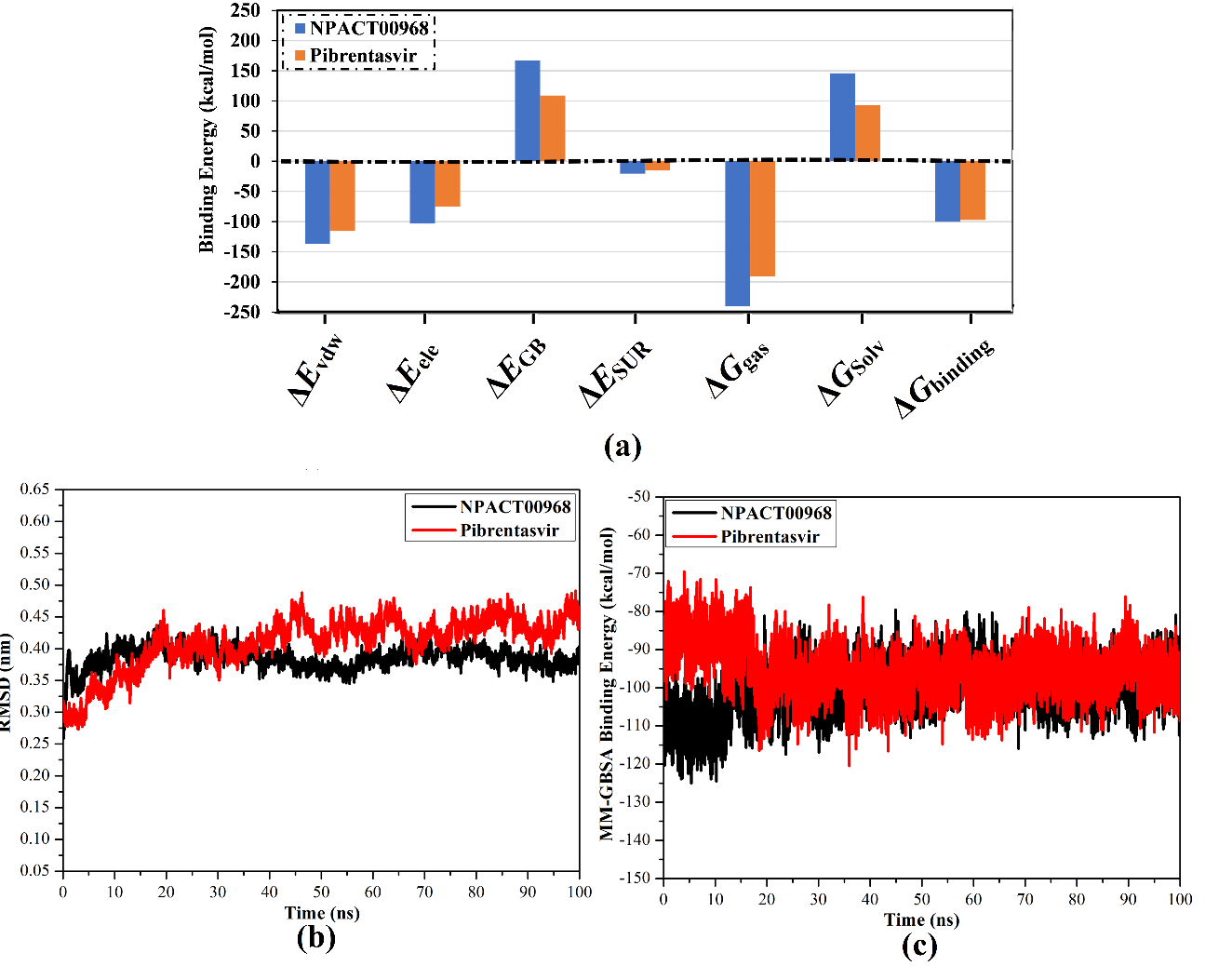


### Figure S4. (a) Components of the MM-GBSA binding energies, (b) root-mean-square deviation (RMSD) of the backbone atoms from the initial structure, and (c) estimated MM-GBSA binding energy per frame of NPACT00968 and pibrentasvir in complex with ABCG2 transporter over the MD course of 100 ns.

**Table S1.** Calculated vina docking scores (in kcal/mol) and MM**-**GBSA//MM binding energies for the BWQ and NPACT compounds towards ABCG2 transporter ^a^.

| No. | CompoundName/ Code | **Docking Score**  **(kcal/mol)** | **MM-GBSA//MM**  **binding energy (kcal/mol)** | No. | Compound Name/ Code | **Docking Score**  **(kcal/mol)** | **MM-GBSA//MM**  **binding energy (kcal/mol)** |
| --- | --- | --- | --- | --- | --- | --- | --- |
|  | BWQ | −10.3 | −60.5 |  |  |  |  |
| ***Terpenoids*** | | | | | | | |
| 1 | NPACT01552 | −10.8 | −127.2 | 49 | NPACT01449 | −9.7 | −67.9 |
| 2 | NPACT00444 | −10.0 | −114.5 | 50 | NPACT00470 | −10.5 | −67.6 |
| 3 | NPACT01557 | −10.0 | −104.0 | 51 | NPACT00592 | −8.5 | −66.9 |
| 4 | NPACT01014 | −8.1 | −95.5 | 52 | NPACT00115 | −8.4 | −66.5 |
| 5 | NPACT00406 | −10.0 | −94.5 | 53 | NPACT00991 | −9.3 | −65.9 |
| 6 | NPACT01016 | −8.1 | −91.4 | 54 | NPACT01444 | −7.7 | −65.8 |
| 7 | NPACT00936 | −9.3 | −88.8 | 55 | NPACT00998 | −9.4 | −65.2 |
| 8 | NPACT00992 | −9.3 | −85.5 | 56 | NPACT01034 | −9.9 | −65.1 |
| 9 | NPACT00650 | −9.0 | −83.3 | 57 | NPACT01450 | −10.0 | −65.1 |
| 10 | NPACT01495 | −9.9 | −83.2 | 58 | NPACT00403 | −8.4 | −64.9 |
| 11 | NPACT00833 | −10.0 | −81.1 | 59 | NPACT01151 | −9.2 | −64.8 |
| 12 | NPACT00834 | −10.4 | −79.8 | 60 | NPACT01459 | −9.3 | −64.8 |
| 13 | NPACT00461 | −9.1 | −79.6 | 61 | NPACT01083 | −10.7 | −64.6 |
| 14 | NPACT00631 | −8.9 | −79.3 | 62 | NPACT00630 | −9.0 | −63.5 |
| 15 | NPACT00141 | −8.7 | −78.6 | 63 | NPACT00898 | −9.4 | −63.4 |
| 16 | NPACT01494 | −10.9 | −78.4 | 64 | NPACT00135 | −9.3 | −63.2 |
| 17 | NPACT00140 | −9.2 | −77.4 | 65 | NPACT00479 | −9.5 | −63.1 |
| 18 | NPACT00469 | −10.7 | −77.1 | 66 | NPACT01207 | −9.5 | −62.9 |
| 19 | NPACT01134 | −8.2 | −76.3 | 67 | NPACT00457 | −9.1 | −62.6 |
| 20 | NPACT00931 | −10.0 | −75.7 | 68 | NPACT00839 | −8.9 | −62.6 |
| 21 | NPACT00763 | −10.3 | −75.6 | 69 | NPACT01488 | −8.8 | −62.6 |
| 22 | NPACT01120 | −9.9 | −75.0 | 70 | NPACT00458 | −9.4 | −62.5 |
| 23 | NPACT00137 | −9.1 | −74.7 | 71 | NPACT01307 | −9.6 | −62.2 |
| 24 | NPACT00374 | −9.8 | −74.1 | 72 | NPACT00651 | −8.8 | −62.1 |
| 25 | NPACT00138 | −8.9 | −74.0 | 73 | NPACT00996 | −10.1 | −61.8 |
| 26 | NPACT01308 | −9.6 | −73.6 | 74 | NPACT01046 | −10.5 | −61.7 |
| 27 | NPACT00125 | −9.4 | −73.5 | 75 | NPACT01522 | −8.2 | −61.7 |
| 28 | NPACT01284 | −10.7 | −73.3 | 76 | NPACT01172 | −10.4 | −61.5 |
| 29 | NPACT01139 | −7.9 | −72.8 | 77 | NPACT00498 | −9.1 | −61.5 |
| 30 | NPACT00375 | −9.8 | −72.7 | 78 | NPACT01031 | −8.5 | −61.4 |
| 31 | NPACT00632 | −9.5 | −72.7 | 79 | NPACT01123 | −9.2 | −61.3 |
| 32 | NPACT01138 | −8.9 | −72.6 | 80 | NPACT01203 | −8.1 | −61.1 |
| 33 | NPACT01521 | −9.9 | −72.6 | 81 | NPACT01532 | −9.8 | −61.1 |
| 34 | NPACT00997 | −10.4 | −71.5 | 82 | NPACT00095 | −10.7 | −61.1 |
| 35 | NPACT01132 | −7.8 | −70.9 | 83 | NPACT01367 | −9.1 | −61.0 |
| 36 | NPACT00728 | −8.7 | −70.8 | 84 | NPACT01133 | −8.3 | −60.5 |
| 37 | NPACT00731 | −8.7 | −70.7 | 85 | NPACT00123 | −9.8 | −60.5 |
| 38 | NPACT00122 | −10.1 | −70.3 | 86 | NPACT00764 | −10.1 | −60.3 |
| 39 | NPACT01017 | −7.3 | −70.0 | 87 | NPACT01515 | −10.2 | −60.2 |
| 40 | NPACT01135 | −8.6 | −69.6 | 88 | NPACT01465 | −7.3 | −60.1 |
| 41 | NPACT00128 | −10.4 | −69.6 | 89 | NPACT00946 | −7.9 | −59.8 |
| 42 | NPACT01173 | −10.7 | −69.4 | 90 | NPACT00127 | −9.7 | −59.8 |
| 43 | NPACT01171 | −11.3 | −68.8 | 91 | NPACT00459 | −7.8 | −59.7 |
| 44 | NPACT00116 | −10.5 | −68.4 | 92 | NPACT00743 | −7.8 | −59.7 |
| 45 | NPACT00762 | −9.0 | −68.3 | 93 | NPACT01072 | −7.4 | −59.6 |
| 46 | NPACT00136 | −9.2 | −68.3 | 94 | NPACT00234 | −9.2 | −59.5 |
| 47 | NPACT01137 | −8.1 | −68.0 | 95 | NPACT01114 | −9.4 | − 59.4 |
| 48 | NPACT01082 | −9.9 | −67.9 | 96 | NPACT00954 | −8.9 | −59.2 |

**Table S1**. *Continued.*

| No. | CompoundName/ Code | Docking Score(kcal/mol) | MM-GBSA//MMbinding energy (kcal/mol) | No. | Compound Name/ Code | Docking Score(kcal/mol) | MM-GBSA//MMbinding energy (kcal/mol) |
| --- | --- | --- | --- | --- | --- | --- | --- |
| 97 | NPACT00305 | −8.4 | −59.1 | 148 | NPACT00259 | −10.6 | −53.1 |
| 98 | NPACT00448 | −8.8 | −59.0 | 149 | NPACT00026 | −8.9 | −53.0 |
| 99 | NPACT00667 | −8.3 | −58.7 | 150 | NPACT00333 | −9.0 | −52.8 |
| 100 | NPACT01239 | −9.0 | −58.7 | 151 | NPACT00663 | −9.4 | −52.8 |
| 101 | NPACT00041 | −9.5 | −58.7 | 152 | NPACT00452 | − 9.1 | − 52.6 |
| 102 | NPACT00832 | −9.9 | −58.5 | 153 | NPACT00692 | − 8.3 | − 52.5 |
| 103 | NPACT00765 | −8.6 | −58.5 | 154 | NPACT00114 | − 9.2 | − 52.4 |
| 104 | NPACT00648 | −8.7 | −58.2 | 155 | NPACT00139 | − 9.1 | − 52.3 |
| 105 | NPACT00694 | −7.8 | −58.2 | 156 | NPACT00363 | − 10.1 | − 52.3 |
| 106 | NPACT01013 | −7.1 | −58.1 | 157 | NPACT01475 | − 9.2 | −52.3 |
| 107 | NPACT00690 | −8.9 | −58.1 | 158 | NPACT00964 | −8.2 | −52.1 |
| 108 | NPACT00702 | −9.2 | −58.0 | 159 | NPACT00146 | −9.9 | −52.0 |
| 109 | NPACT01000 | −10.2 | −57.8 | 160 | NPACT00113 | −9.3 | −52.0 |
| 110 | NPACT00055 | −8.5 | −57.8 | 161 | NPACT00075 | −8.5 | −52.0 |
| 111 | NPACT00219 | −8.8 | −57.5 | 162 | NPACT00993 | −7.8 | −51.9 |
| 112 | NPACT01136 | −8.3 | −57.4 | 163 | NPACT01473 | −7.2 | −51.9 |
| 113 | NPACT00094 | −10.2 | −57.2 | 164 | NPACT01174 | −9.7 | −51.8 |
| 114 | NPACT00054 | −8.5 | −57.1 | 165 | NPACT00306 | −8.8 | −51.7 |
| 115 | NPACT00514 | −7.9 | −56.9 | 166 | NPACT00484 | −9.2 | −51.6 |
| 116 | NPACT00649 | −9.4 | −56.9 | 167 | NPACT00345 | −8.7 | −51.6 |
| 117 | NPACT00126 | −10.8 | −56.7 | 168 | NPACT01504 | −9.5 | −51.6 |
| 118 | NPACT00036 | −9.0 | −56.6 | 169 | NPACT00076 | −8.4 | −51.5 |
| 119 | NPACT00309 | −9.5 | −56.6 | 170 | NPACT00564 | −8.8 | −51.4 |
| 120 | NPACT01058 | −7.0 | −56.3 | 171 | NPACT00400 | −8.2 | −51.4 |
| 121 | NPACT01513 | −9.3 | −56.1 | 172 | NPACT01370 | −8.7 | −51.3 |
| 122 | NPACT01206 | −9.0 | −56.1 | 173 | NPACT00307 | −9.1 | −51.2 |
| 123 | NPACT00446 | −8.9 | −56.0 | 174 | NPACT00462 | −8.8 | −51.2 |
| 124 | NPACT00460 | −8.6 | −55.9 | 175 | NPACT00401 | −8.3 | −51.0 |
| 125 | NPACT00414 | −7.8 | −55.8 | 176 | NPACT00043 | −9.0 | −51.0 |
| 126 | NPACT00693 | −8.1 | −55.8 | 177 | NPACT00074 | −8.3 | −51.0 |
| 127 | NPACT00344 | −8.6 | −55.7 | 178 | NPACT00093 | −9.6 | −50.9 |
| 128 | NPACT00758 | −8.6 | −55.5 | 179 | NPACT00086 | −8.9 | −50.9 |
| 129 | NPACT01452 | −9.4 | −55.5 | 180 | NPACT00308 | −7.9 | −50.8 |
| 130 | NPACT00405 | −8.4 | −55.3 | 181 | NPACT00069 | −10.2 | −50.7 |
| 131 | NPACT00880 | −9.2 | −55.2 | 182 | NPACT01001 | −9.4 | −50.7 |
| 132 | NPACT01366 | −9.2 | −55.0 | 183 | NPACT00058 | −8.1 | −50.6 |
| 133 | NPACT01440 | −8.6 | −54.9 | 184 | NPACT00481 | −9.4 | −50.5 |
| 134 | NPACT00147 | −7.8 | −54.9 | 185 | NPACT00038 | −10.1 | −50.5 |
| 135 | NPACT01113 | −9.5 | −54.8 | 186 | NPACT00766 | −9.0 | −50.4 |
| 136 | NPACT00761 | −10.4 | −54.7 | 187 | NPACT01105 | −7.1 | −50.4 |
| 137 | NPACT00098 | −9.4 | −54.7 | 188 | NPACT01260 | −8.6 | −50.4 |
| 138 | NPACT00072 | −9.1 | −54.2 | 189 | NPACT00351 | −8.4 | −50.3 |
| 139 | NPACT01531 | −8.3 | −54.2 | 190 | NPACT00451 | −8.7 | −50.3 |
| 140 | NPACT00303 | −8.0 | −53.9 | 191 | NPACT00455 | −8.9 | −50.1 |
| 141 | NPACT00310 | −9.2 | −53.8 | 192 | NPACT00077 | −8.8 | −50.0 |
| 142 | NPACT00449 | −8.6 | −53.6 | 193 | NPACT00647 | −8.5 | −49.9 |
| 143 | NPACT00278 | −9.9 | −53.5 | 194 | NPACT00348 | −7.8 | −49.8 |
| 144 | NPACT00056 | −8.5 | −53.4 | 195 | NPACT00332 | −9.1 | −49.7 |
| 145 | NPACT00988 | −9.6 | −53.3 | 196 | NPACT01460 | −7.7 | −49.6 |
| 146 | NPACT01369 | −8.8 | −53.2 | 197 | NPACT01129 | −7.6 | −49.6 |
| 147 | NPACT00695 | −8.0 | −53.1 | 198 | NPACT00230 | −9.8 | −49.6 |

**Table S1**. *Continued.*

| No. | CompoundName/ Code | Docking Score(kcal/mol) | MM-GBSA//MMbinding energy (kcal/mol) | No. | CompoundName/ Code | Docking Score(kcal/mol) | MM-GBSA//MMbinding energy (kcal/mol) |
| --- | --- | --- | --- | --- | --- | --- | --- |
| 199 | NPACT01116 | −7.9 | −49.5 | 250 | NPACT00232 | −10.5 | −45.4 |
| 200 | NPACT00691 | −7.9 | −49.4 | 251 | NPACT01431 | −8.8 | −45.3 |
| 201 | NPACT01408 | −7.1 | −49.4 | 252 | NPACT00454 | −8.5 | −45.3 |
| 202 | NPACT00887 | −8.7 | −49.3 | 253 | NPACT01512 | −7.7 | −45.2 |
| 203 | NPACT01518 | −8.2 | −49.1 | 254 | NPACT00411 | − 9.2 | − 45.1 |
| 204 | NPACT01368 | −9.3 | −49.1 | 255 | NPACT01458 | − 6.6 | − 44.9 |
| 205 | NPACT00705 | −10.1 | −49.0 | 256 | NPACT01225 | − 8.5 | −44.9 |
| 206 | NPACT00652 | −8.5 | −48.9 | 257 | NPACT00466 | −8.8 | −44.8 |
| 207 | NPACT00447 | −9.1 | −48.8 | 258 | NPACT00037 | −9.2 | −44.6 |
| 208 | NPACT01290 | −9.0 | −48.7 | 259 | NPACT01253 | −8.2 | −44.3 |
| 209 | NPACT00450 | −9.3 | −48.7 | 260 | NPACT01402 | −9.3 | −44.3 |
| 210 | NPACT00901 | −10.0 | −48.6 | 261 | NPACT01467 | −7.6 | −44.1 |
| 211 | NPACT01363 | −9.3 | −48.5 | 262 | NPACT00097 | − 9.1 | −44.0 |
| 212 | NPACT00071 | −9.9 | −48.5 | 263 | NPACT00815 | −9.2 | −43.8 |
| 213 | NPACT00091 | −10.0 | −48.3 | 264 | NPACT00688 | −8.9 | −43.7 |
| 214 | NPACT00365 | −9.7 | −48.2 | 265 | NPACT00042 | −8.5 | −43.7 |
| 215 | NPACT00164 | −8.3 | −48.2 | 266 | NPACT01380 | −8.3 | −43.6 |
| 216 | NPACT00897 | −8.6 | −48.1 | 267 | NPACT00566 | −8.1 | −43.6 |
| 217 | NPACT00983 | −7.9 | −48.0 | 268 | NPACT00004 | −8.1 | −43.5 |
| 218 | NPACT00443 | −8.3 | −48.0 | 269 | NPACT00784 | −7.6 | −43.4 |
| 219 | NPACT00902 | −9.4 | −48.0 | 270 | NPACT00407 | −8.5 | −43.4 |
| 220 | NPACT00349 | −7.9 | −47.7 | 271 | NPACT01386 | −9.0 | −43.1 |
| 221 | NPACT00539 | −8.8 | −47.7 | 272 | NPACT00604 | −7.1 | −43.0 |
| 222 | NPACT01176 | −8.9 | −47.6 | 273 | NPACT00947 | −8.4 | −42.8 |
| 223 | NPACT01197 | −8.6 | −47.6 | 274 | NPACT00982 | −8.0 | −42.8 |
| 224 | NPACT01525 | −8.3 | −47.6 | 275 | NPACT01041 | −7.9 | −42.7 |
| 225 | NPACT01033 | −10 | −47.5 | 276 | NPACT00223 | −8.2 | −42.7 |
| 226 | NPACT00519 | −8.6 | −47.4 | 277 | NPACT00089 | −8.7 | −42.7 |
| 227 | NPACT00487 | −9.1 | −47.4 | 278 | NPACT01025 | −7.5 | −42.6 |
| 228 | NPACT00585 | −10.2 | −47.2 | 279 | NPACT00402 | −8.1 | −42.6 |
| 229 | NPACT00453 | −9.1 | −47.1 | 280 | NPACT01286 | −8.9 | −42.6 |
| 230 | NPACT00900 | −7.4 | −46.9 | 281 | NPACT01057 | −9.1 | −42.5 |
| 231 | NPACT00304 | −7.9 | −46.9 | 282 | NPACT00895 | −7.6 | −42.3 |
| 232 | NPACT00243 | −7.7 | −46.8 | 283 | NPACT00628 | −8.5 | −42.3 |
| 233 | NPACT00689 | −7.9 | −46.7 | 284 | NPACT00532 | −8.3 | −42.2 |
| 234 | NPACT00245 | −7.7 | −46.6 | 285 | NPACT00392 | −7.9 | −42.0 |
| 235 | NPACT01443 | −9.5 | −46.6 | 286 | NPACT01486 | −8.1 | −42.0 |
| 236 | NPACT00350 | −8.6 | −46.6 | 287 | NPACT00533 | −8.7 | −42.0 |
| 237 | NPACT00899 | −8.0 | −46.6 | 288 | NPACT01372 | −7.6 | −42.0 |
| 238 | NPACT01469 | −10.5 | −46.5 | 289 | NPACT00410 | −8.3 | −41.9 |
| 239 | NPACT00774 | −9.7 | −46.4 | 290 | NPACT01340 | −8.4 | −41.9 |
| 240 | NPACT01517 | −7.8 | −46.4 | 291 | NPACT00099 | −8.8 | −41.8 |
| 241 | NPACT00726 | −9.5 | −46.3 | 292 | NPACT00409 | −8.4 | −41.6 |
| 242 | NPACT00827 | −9.1 | −46.2 | 293 | NPACT00636 | −9.4 | −41.6 |
| 243 | NPACT00828 | −9.4 | −46.1 | 294 | NPACT00242 | −7.9 | −41.4 |
| 244 | NPACT00364 | −9.7 | −46.1 | 295 | NPACT00440 | −8.5 | −41.3 |
| 245 | NPACT00826 | −9.5 | −45.8 | 296 | NPACT01188 | −8.7 | −41.2 |
| 246 | NPACT00244 | −7.7 | −45.7 | 297 | NPACT01338 | −9.1 | −41.1 |
| 247 | NPACT01539 | −7.5 | −45.6 | 298 | NPACT00627 | −8.1 | −40.9 |
| 248 | NPACT00831 | −8.7 | −45.6 | 299 | NPACT01148 | −9.2 | −40.9 |
| 249 | NPACT00399 | −8.2 | −45.6 | 300 | NPACT00465 | −8.3 | −40.8 |

**Table S1**. *Continued.*

| No. | CompoundName/ Code | Docking Score(kcal/mol) | MM-GBSA//MMbinding energy (kcal/mol) | No. | CompoundName/ Code | Docking Score(kcal/mol) | MM-GBSA//MMbinding energy (kcal/mol) |
| --- | --- | --- | --- | --- | --- | --- | --- |
| 301 | NPACT00199 | −7.3 | −40.8 | 352 | NPACT01481 | −7.9 | −37.1 |
| 302 | NPACT00160 | −8.8 | −40.6 | 353 | NPACT00415 | −7.9 | −36.8 |
| 303 | NPACT00391 | −7.5 | −40.6 | 354 | NPACT01528 | −7.6 | −36.8 |
| 304 | NPACT00534 | −8.3 | −40.6 | 355 | NPACT01121 | −8.0 | −36.7 |
| 305 | NPACT00565 | −8.2 | −40.5 | 356 | NPACT00291 | −8.0 | −36.7 |
| 306 | NPACT00535 | −9.1 | −40.4 | 357 | NPACT01106 | −7.5 | −36.6 |
| 307 | NPACT00433 | −6.5 | −40.3 | 358 | NPACT00390 | −7.2 | −36.6 |
| 308 | NPACT00096 | −8.2 | −40.3 | 359 | NPACT00110 | −7.7 | −36.4 |
| 309 | NPACT01339 | −7.7 | −40.3 | 360 | NPACT01479 | −7.5 | −36.4 |
| 310 | NPACT01523 | −8.5 | −40.2 | 361 | NPACT01078 | −8.9 | −36.4 |
| 311 | NPACT01487 | −9.0 | −40.2 | 362 | NPACT00927 | −7.3 | −36.3 |
| 312 | NPACT01140 | −7.6 | −40.1 | 363 | NPACT01422 | −8.4 | −36.2 |
| 313 | NPACT01409 | −8.0 | −40.0 | 364 | NPACT01569 | −8.0 | −36.1 |
| 314 | NPACT01256 | −7.7 | −39.8 | 365 | NPACT00338 | −7.3 | −36.1 |
| 315 | NPACT00040 | −7.3 | −39.8 | 366 | NPACT01184 | −8.5 | −36.1 |
| 316 | NPACT01529 | −8.8 | −39.8 | 367 | NPACT00225 | −7.9 | −36.0 |
| 317 | NPACT01344 | −5.9 | −39.8 | 368 | NPACT00052 | −8.3 | −36.0 |
| 318 | NPACT01233 | −7.9 | −39.8 | 369 | NPACT00312 | −7.9 | −36.0 |
| 319 | NPACT00051 | −9.0 | −39.6 | 370 | NPACT01445 | −7.6 | −36.0 |
| 320 | NPACT01538 | −8.3 | −39.6 | 371 | NPACT00336 | −7.6 | −35.8 |
| 321 | NPACT00121 | −8.1 | −39.5 | 372 | NPACT01425 | −8.0 | −35.5 |
| 322 | NPACT00673 | −7.4 | −39.5 | 373 | NPACT00112 | −7.3 | −35.5 |
| 323 | NPACT00978 | −7.9 | −39.4 | 374 | NPACT00874 | −7.9 | −35.3 |
| 324 | NPACT00467 | −8.7 | −39.2 | 375 | NPACT01566 | −8.5 | −35.1 |
| 325 | NPACT00196 | −8.2 | −39.1 | 376 | NPACT00092 | −9.5 | −35.1 |
| 326 | NPACT01353 | −7.5 | −39.0 | 377 | NPACT00953 | −8.6 | −35.1 |
| 327 | NPACT00150 | −8.2 | −38.8 | 378 | NPACT00435 | −7.5 | −35.0 |
| 328 | NPACT01261 | −8.1 | −38.8 | 379 | NPACT01107 | −4.6 | −35.0 |
| 329 | NPACT01541 | −8.1 | −38.7 | 380 | NPACT01022 | −7.7 | −34.9 |
| 330 | NPACT00070 | −9.0 | −38.7 | 381 | NPACT01259 | −7.7 | −34.7 |
| 331 | NPACT00528 | −8.5 | −38.6 | 382 | NPACT01441 | −8.1 | −34.6 |
| 332 | NPACT00738 | −7.6 | −38.5 | 383 | NPACT00100 | −8.9 | −34.6 |
| 333 | NPACT00434 | −8.4 | −38.5 | 384 | NPACT00767 | −5.9 | −34.6 |
| 334 | NPACT01526 | −8.4 | −38.4 | 385 | NPACT00529 | −8.6 | −34.4 |
| 335 | NPACT00608 | −6.8 | −38.1 | 386 | NPACT01354 | −8.2 | −34.3 |
| 336 | NPACT01371 | −7.2 | −38.0 | 387 | NPACT00603 | −6.4 | −34.2 |
| 337 | NPACT00393 | −8.2 | −38.0 | 388 | NPACT01117 | −8.0 | −34.2 |
| 338 | NPACT01183 | −6.9 | −37.8 | 389 | NPACT00323 | −6.8 | −34.1 |
| 339 | NPACT00577 | −6.7 | −37.7 | 390 | NPACT01537 | −8.9 | −34.0 |
| 340 | NPACT01026 | −8.3 | −37.6 | 391 | NPACT00464 | −8.4 | −34.0 |
| 341 | NPACT00610 | −6.8 | −37.6 | 392 | NPACT00609 | −6.7 | −34.0 |
| 342 | NPACT01352 | −7.9 | −37.4 | 393 | NPACT01453 | −7.5 | −34.0 |
| 343 | NPACT00468 | −9.2 | −37.4 | 394 | NPACT01490 | −8.1 | −34.0 |
| 344 | NPACT01267 | −8.7 | −37.4 | 395 | NPACT00681 | −7.7 | −33.9 |
| 345 | NPACT00661 | −8.5 | −37.3 | 396 | NPACT00552 | −7.5 | −33.8 |
| 346 | NPACT00813 | −7.0 | −37.3 | 397 | NPACT00408 | −7.9 | −33.8 |
| 347 | NPACT00200 | −8.1 | −37.2 | 398 | NPACT00299 | −7.0 | −33.6 |
| 348 | NPACT00483 | −8.0 | −37.1 | 399 | NPACT00934 | −8.5 | −33.6 |
| 349 | NPACT01043 | −8.6 | −37.1 | 400 | NPACT00530 | −8.2 | −33.5 |
| 350 | NPACT01127 | −8.6 | −37.1 | 401 | NPACT01390 | −7.9 | −33.4 |
| 351 | NPACT01509 | −8.6 | −37.1 | 402 | NPACT00233 | −8.1 | −33.3 |

**Table S1**. *Continued.*

| No. | CompoundName/ Code | Docking Score(kcal/mol) | MM-GBSA//MMbinding energy (kcal/mol) | No. | CompoundName/ Code | Docking Score(kcal/mol) | MM-GBSA//MMbinding energy (kcal/mol) |  |
| --- | --- | --- | --- | --- | --- | --- | --- | --- |
| 403 | NPACT01462 | −6.6 | −33.3 | 450 | NPACT00851 | −6.1 | −28.0 |  |
| 404 | NPACT00966 | −7.5 | −33.1 | 451 | NPACT01306 | −7.4 | −27.9 |  |
| 405 | NPACT00337 | −7.5 | −33.0 | 452 | NPACT00354 | −6.9 | −27.7 |  |
| 406 | NPACT01159 | −7.5 | −33.0 | 453 | NPACT00193 | −8.1 | −27.6 |  |
| 407 | NPACT00626 | −7.7 | −32.9 | 454 | NPACT00871 | −5.9 | −27.5 |  |
| 408 | NPACT01265 | −7.8 | −32.9 | 455 | NPACT01182 | −7.3 | −27.4 |  |
| 409 | NPACT01221 | −7.8 | −32.8 | 456 | NPACT00508 | −7.1 | −27.1 |  |
| 410 | NPACT00053 | −6.6 | −32.6 | 457 | NPACT01004 | −6.7 | −27.1 |  |
| 411 | NPACT00432 | −6.1 | −32.6 | 458 | NPACT01237 | −7.4 | −27.1 |  |
| 412 | NPACT01266 | −7.4 | −32.5 | 459 | NPACT00607 | −5.8 | −27.1 |  |
| 413 | NPACT00352 | −7.4 | −32.5 | 460 | NPACT01202 | −7.6 | −26.9 |  |
| 414 | NPACT01040 | −8.5 | −32.4 | 461 | NPACT00578 | −5.7 | −26.9 |  |
| 415 | NPACT01385 | −7.4 | −32.2 | 462 | NPACT01236 | −7.0 | −26.5 |  |
| 416 | NPACT01382 | −7.0 | −32.2 | 463 | NPACT01125 | −7.6 | −26.4 |  |
| 417 | NPACT01145 | −6.5 | −32.1 | 464 | NPACT00206 | −7.6 | −26.0 |  |
| 418 | NPACT00537 | −8.2 | −31.9 | 465 | NPACT00185 | −7.4 | −26.0 |  |
| 419 | NPACT01281 | −6.5 | −31.9 | 466 | NPACT00714 | −5.9 | −25.9 |  |
| 420 | NPACT01196 | −7.8 | −31.8 | 467 | NPACT00353 | −6.9 | −25.9 |  |
| 421 | NPACT01150 | −6.4 | −31.4 | 468 | NPACT00240 | −6.3 | −25.7 |  |
| 422 | NPACT01446 | −7.4 | −31.3 | 469 | NPACT01149 | −5.5 | −25.7 |  |
| 423 | NPACT00044 | −8.6 | −31.2 | 470 | NPACT01349 | −6.2 | −25.4 |  |
| 424 | NPACT00151 | −7.4 | −31.1 | 471 | NPACT01175 | −6.0 | −25.1 |  |
| 425 | NPACT00039 | −6.6 | −30.8 | 472 | NPACT00515 | −6.6 | −25.0 |  |
| 426 | NPACT00324 | −6.9 | −30.8 | 473 | NPACT01323 | −6.8 | −24.8 |  |
| 427 | NPACT00431 | −6.1 | −30.7 | 474 | NPACT00321 | −6.9 | −24.5 |  |
| 428 | NPACT00606 | −6.4 | −30.6 | 475 | NPACT00397 | −6.8 | −24.4 |  |
| 429 | NPACT00844 | −7.1 | −30.5 | 476 | NPACT00209 | −7.3 | −24.2 |  |
| 430 | NPACT01220 | −7.9 | −30.5 | 477 | NPACT00789 | −5.9 | −24.2 |  |
| 431 | NPACT00236 | −7.2 | −30.4 | 478 | NPACT00568 | −7.0 | −24.0 |  |
| 432 | NPACT01346 | −7.3 | −30.3 | 479 | NPACT00823 | −5.9 | −23.6 |  |
| 433 | NPACT01254 | −7.8 | −30.2 | 480 | NPACT00421 | −5.6 | −23.6 |  |
| 434 | NPACT01350 | −7.0 | −29.8 | 481 | NPACT00238 | −5.6 | −23.6 |  |
| 435 | NPACT01156 | −5.8 | −29.7 | 482 | NPACT01038 | −6.0 | −22.8 |  |
| 436 | NPACT00843 | −7.2 | −29.6 | 483 | NPACT00975 | −6.3 | −22.6 |  |
| 437 | NPACT01379 | −7.7 | −29.6 | 484 | NPACT01146 | −6.2 | −21.0 |  |
| 438 | NPACT01213 | −6.6 | −29.5 | 485 | NPACT00850 | −6.0 | −21.0 |  |
| 439 | NPACT00334 | −7.7 | −29.5 | 486 | NPACT00793 | −5.5 | −21.0 |  |
| 440 | NPACT00203 | −7.6 | −29.4 | 487 | NPACT01147 | −5.7 | −20.3 |  |
| 441 | NPACT00531 | −7.7 | −29.2 | 488 | NPACT00442 | −5.3 | −19.6 |  |
| 442 | NPACT01255 | −8.0 | −29.1 | 489 | NPACT00388 | −5.6 | −19.0 |  |
| 443 | NPACT00768 | −6.1 | −28.6 | 490 | NPACT00579 | −5.8 | −18.9 |  |
| 444 | NPACT00207 | −7.8 | −28.5 | 491 | NPACT01355 | −5.5 | −17.9 |  |
| 445 | NPACT01303 | −6.4 | −28.5 | 492 | NPACT00904 | −5.1 | −17.9 |  |
| 446 | NPACT00441 | −7.5 | −28.4 | 493 | NPACT00586 | −6.5 | −17.8 |  |
| 447 | NPACT00235 | −6.4 | −28.4 | 494 | NPACT00527 | −6.4 | −17.6 |  |
| 448 | NPACT00971 | −6.1 | −28.0 | 495 | NPACT01015 | −7.4 | −17.5 |  |
| 449 | NPACT00398 | −7.0 | −28.0 | 496 | NPACT00429 | −7.8 | −17.1 |  |
| ***Flavonoids*** | | | | | | | | |
| 500 | NPACT00746 | −8.1 | −97.7 | 503 | NPACT00339 | −10.0 | −84.9 |  |
| 501 | NPACT01457 | −8.8 | −97.6 | 504 | NPACT00343 | −9.9 | −78.4 |  |
| 502 | NPACT01312 | −10.4 | −85.2 | 505 | NPACT00340 | −9.8 | −75.3 |  |

**Table S1**. *Continued.*

| No. | CompoundName/ Code | Docking Score(kcal/mol) | MM-GBSA//MMbinding energy (kcal/mol) | No. | CompoundName/ Code | Docking Score(kcal/mol) | MM-GBSA//MMbinding energy (kcal/mol) |
| --- | --- | --- | --- | --- | --- | --- | --- |
| 506 | NPACT00680 | −10.1 | −75.0 | 557 | NPACT01115 | −11.0 | −55.6 |
| 507 | NPACT00977 | −9.5 | −73.6 | 558 | NPACT01047 | −8.4 | −55.2 |
| 508 | NPACT00807 | −9.6 | −72.5 | 559 | NPACT01314 | −8.9 | −55.1 |
| 509 | NPACT00545 | −10.3 | −71.3 | 560 | NPACT00359 | −10.2 | −55.0 |
| 510 | NPACT00542 | −10.4 | −70.7 | 561 | NPACT00662 | −9.1 | −54.8 |
| 511 | NPACT00381 | −11.0 | −70.6 | 562 | NPACT00286 | −9.2 | −54.7 |
| 512 | NPACT00541 | −9.8 | −70.2 | 563 | NPACT01365 | −8.6 | −54.5 |
| 513 | NPACT00511 | −9.8 | −70.2 | 564 | NPACT00801 | −7.8 | −54.3 |
| 514 | NPACT01554 | −9.5 | −70.1 | 565 | NPACT01389 | −10.0 | −54.1 |
| 515 | NPACT00660 | −9.9 | −69.7 | 566 | NPACT01347 | − 9.1 | −54.0 |
| 516 | NPACT00878 | −10.1 | −69.7 | 567 | NPACT01088 | −8.3 | −53.7 |
| 517 | NPACT01553 | −9.4 | −69.4 | 568 | NPACT00679 | −8.5 | −53.7 |
| 518 | NPACT00553 | −9.7 | −68.7 | 569 | NPACT00548 | −8.9 | −53.7 |
| 519 | NPACT00256 | −11.7 | −68.3 | 570 | NPACT00383 | −10.0 | −53.6 |
| 520 | NPACT00918 | −10.2 | −67.6 | 571 | NPACT00929 | −9.5 | −53.5 |
| 521 | NPACT00809 | −9.8 | −67.5 | 572 | NPACT01311 | −9.8 | −53.5 |
| 522 | NPACT00283 | −11.1 | −66.4 | 573 | NPACT00387 | −10.4 | −53.2 |
| 523 | NPACT00195 | −11.1 | −66.4 | 574 | NPACT00285 | −9.7 | −53.0 |
| 524 | NPACT00342 | −11.5 | −65.8 | 575 | NPACT00560 | −10.1 | −52.9 |
| 525 | NPACT00540 | −10.1 | −65.8 | 576 | NPACT00699 | −8.5 | −52.6 |
| 526 | NPACT00288 | −10.5 | −65.6 | 577 | NPACT00380 | −10.6 | −52.6 |
| 527 | NPACT00386 | −10.9 | −65.4 | 578 | NPACT00837 | −9.2 | −52.5 |
| 528 | NPACT00808 | −10.0 | −65.2 | 579 | NPACT00928 | −8.8 | −52.3 |
| 529 | NPACT00800 | −9.7 | −64.9 | 580 | NPACT01118 | −7.1 | −52.0 |
| 530 | NPACT00189 | −10.9 | −64.0 | 581 | NPACT00281 | −9.9 | −51.8 |
| 531 | NPACT01309 | −10.2 | −63.8 | 582 | NPACT00562 | −9.3 | −51.7 |
| 532 | NPACT00384 | −10.6 | −63.7 | 583 | NPACT00790 | −6.2 | −51.6 |
| 533 | NPACT00543 | −10.3 | −62.7 | 584 | NPACT01322 | −8.9 | −51.4 |
| 534 | NPACT00494 | −10.3 | −62.7 | 585 | NPACT01119 | −7.1 | −51.4 |
| 535 | NPACT01318 | −10.7 | −61.4 | 586 | NPACT00271 | −9.2 | −51.4 |
| 536 | NPACT00176 | −10.4 | −61.0 | 587 | NPACT00377 | −9.7 | −51.1 |
| 537 | NPACT01315 | −9.6 | −60.6 | 588 | NPACT00376 | −10.0 | −51.0 |
| 538 | NPACT01403 | −10.8 | −60.4 | 589 | NPACT01243 | −8.2 | −50.9 |
| 539 | NPACT00799 | −10.1 | −59.9 | 590 | NPACT01378 | −9.6 | −50.8 |
| 540 | NPACT00617 | −11.7 | −59.9 | 591 | NPACT00930 | −9.2 | −50.7 |
| 541 | NPACT00284 | −10.0 | −59.6 | 592 | NPACT00792 | −8.4 | −50.4 |
| 542 | NPACT00197 | −9.8 | −59.2 | 593 | NPACT01419 | −9.1 | −50.4 |
| 543 | NPACT01474 | −9.5 | −59.2 | 594 | NPACT00727 | −9.8 | −49.9 |
| 544 | NPACT00559 | −9.4 | −58.6 | 595 | NPACT01244 | −9.1 | −49.9 |
| 545 | NPACT00279 | −10.1 | −58.6 | 582 | NPACT00948 | −8.5 | −49.9 |
| 546 | NPACT00544 | −9.4 | −58.3 | 583 | NPACT00280 | −9.6 | −49.8 |
| 547 | NPACT00341 | −10.3 | −58.2 | 584 | NPACT00841 | −9.0 | −49.6 |
| 548 | NPACT00287 | −10.2 | −57.8 | 585 | NPACT00613 | −8.9 | −49.5 |
| 549 | NPACT00769 | −7.0 | −57.8 | 586 | NPACT00184 | −9.0 | −49.5 |
| 550 | NPACT00107 | −9.1 | −57.7 | 587 | NPACT00108 | −9.0 | −49.3 |
| 551 | NPACT00382 | −10.2 | −57.3 | 588 | NPACT00611 | −9.6 | −49.2 |
| 552 | NPACT01321 | −9.3 | −57.1 | 589 | NPACT01193 | −9.2 | −49.0 |
| 553 | NPACT01337 | −9.4 | −56.8 | 590 | NPACT01216 | −9.2 | −49.0 |
| 554 | NPACT00700 | −10.0 | −56.4 | 591 | NPACT00172 | −7.6 | −48.9 |
| 555 | NPACT00730 | −10.7 | −55.9 | 592 | NPACT00105 | −8.2 | −48.7 |
| 556 | NPACT01313 | −9.3 | −55.8 | 593 | NPACT00817 | −7.9 | −48.4 |

**Table S1**. *Continued.*

| No. | CompoundName/ Code | Docking Score(kcal/mol) | **MM-GBSA//MM** binding energy (kcal/mol) | No. | CompoundName/ Code | Docking Score(kcal/mol) | **MM-GBSA//MM** binding energy (kcal/mol) |
| --- | --- | --- | --- | --- | --- | --- | --- |
| 594 | NPACT00413 | −8.3 | −48.3 | 645 | NPACT01248 | −10.6 | −43.0 |
| 595 | NPACT00967 | −7.7 | −48.3 | 646 | NPACT01049 | −9.3 | −42.8 |
| 596 | NPACT00385 | −9.5 | −48.2 | 647 | NPACT00169 | −7.7 | −42.8 |
| 597 | NPACT00183 | −9.0 | −48.0 | 648 | NPACT01199 | −8.7 | −42.7 |
| 598 | NPACT01141 | −7.5 | −47.9 | 649 | NPACT01447 | −7.3 | −42.6 |
| 599 | NPACT01567 | −9.9 | −47.7 | 650 | NPACT00517 | −7.5 | −42.6 |
| 600 | NPACT01087 | −7.6 | −47.5 | 651 | NPACT01414 | −8.2 | −42.5 |
| 601 | NPACT01037 | −8.5 | −47.4 | 652 | NPACT01508 | −8.9 | −42.3 |
| 602 | NPACT01287 | −9.8 | −47.3 | 653 | NPACT00710 | −8.6 | −42.2 |
| 603 | NPACT00942 | −7.2 | −47.1 | 654 | NPACT00282 | −9.0 | −42.1 |
| 604 | NPACT00378 | −8.1 | −47.0 | 655 | NPACT01224 | −7.7 | −42.1 |
| 605 | NPACT00701 | −8.6 | −47.0 | 656 | NPACT00791 | −8.3 | −42.1 |
| 606 | NPACT00104 | −7.7 | −47.0 | 657 | NPACT00162 | −8.6 | −41.9 |
| 607 | NPACT01155 | −8.5 | −46.8 | 658 | NPACT01024 | −8.0 | −41.8 |
| 608 | NPACT00892 | −8.0 | −46.7 | 659 | NPACT00111 | −8.4 | −41.7 |
| 609 | NPACT00222 | −9.7 | −46.6 | 660 | NPACT01158 | −8.9 | −41.6 |
| 610 | NPACT00973 | −8.8 | −46.5 | 661 | NPACT01075 | −7.7 | −41.6 |
| 611 | NPACT01283 | −8.9 | −46.2 | 662 | NPACT01092 | −8.2 | −41.5 |
| 612 | NPACT01102 | −9.9 | −46.2 | 663 | NPACT01009 | −7.2 | −41.4 |
| 613 | NPACT00558 | −10.3 | −46.1 | 664 | NPACT01080 | −9.0 | −41.4 |
| 614 | NPACT00974 | −8.4 | −45.9 | 665 | NPACT00106 | −8.6 | −41.2 |
| 615 | NPACT00103 | −9.0 | −45.9 | 666 | NPACT01200 | −9.0 | −41.2 |
| 616 | NPACT01320 | −8.2 | −45.8 | 667 | NPACT00182 | −8.2 | −40.9 |
| 617 | NPACT01468 | −8.6 | −45.8 | 668 | NPACT01299 | −8.3 | −40.9 |
| 618 | NPACT00302 | −9.3 | −45.7 | 669 | NPACT00018 | −6.9 | −40.8 |
| 619 | NPACT00612 | −9.1 | −45.6 | 670 | NPACT00463 | −8.2 | −40.8 |
| 620 | NPACT01126 | −8.6 | −45.6 | 671 | NPACT00170 | −8.6 | −40.8 |
| 621 | NPACT01198 | −9.4 | −45.6 | 672 | NPACT01466 | −8.4 | −40.6 |
| 622 | NPACT01534 | −8.1 | −45.6 | 673 | NPACT00478 | −7.3 | −40.6 |
| 623 | NPACT00778 | −8.7 | −45.4 | 674 | NPACT01010 | −7.5 | −40.6 |
| 624 | NPACT00712 | −7.6 | −45.0 | 675 | NPACT00886 | −9.2 | −40.5 |
| 625 | NPACT01429 | −9.2 | −45.0 | 676 | NPACT01030 | −8.0 | −40.5 |
| 626 | NPACT00524 | −9.3 | −44.9 | 677 | NPACT01400 | −7.9 | −40.5 |
| 627 | NPACT01170 | −8.3 | −44.9 | 678 | NPACT00698 | −8.4 | −40.4 |
| 628 | NPACT01456 | −8.5 | −44.6 | 679 | NPACT00682 | −8.8 | −40.4 |
| 629 | NPACT00165 | −9.3 | −44.3 | 680 | NPACT00019 | −7.1 | −40.3 |
| 630 | NPACT01381 | −9.1 | −44.2 | 681 | NPACT00480 | −9.1 | −40.3 |
| 631 | NPACT01247 | −8.1 | −44.1 | 682 | NPACT01100 | −8.4 | −40.1 |
| 632 | NPACT00549 | −8.7 | −44.1 | 683 | NPACT01021 | −8.5 | −40.1 |
| 633 | NPACT01298 | −7.8 | −44.1 | 684 | NPACT01093 | −7.1 | −40.0 |
| 634 | NPACT01048 | −8.1 | −43.9 | 685 | NPACT00171 | −8.1 | −39.9 |
| 635 | NPACT00879 | −9.3 | −43.8 | 686 | NPACT01434 | −8.9 | −39.9 |
| 636 | NPACT01235 | −10.3 | −43.8 | 687 | NPACT00497 | −8.0 | −39.9 |
| 637 | NPACT00173 | −8.5 | −43.7 | 688 | NPACT01068 | −8.2 | −39.8 |
| 638 | NPACT00557 | −8.4 | −43.7 | 689 | NPACT01436 | −7.7 | −39.7 |
| 639 | NPACT00804 | −9.1 | −43.4 | 690 | NPACT00210 | −8.5 | −39.6 |
| 640 | NPACT00571 | −8.3 | −43.3 | 691 | NPACT00066 | −8.1 | −39.5 |
| 641 | NPACT01310 | −9.5 | −43.1 | 692 | NPACT01084 | −7.9 | −39.5 |
| 642 | NPACT01257 | −9.6 | −43.1 | 693 | NPACT01098 | −8.0 | −39.2 |
| 643 | NPACT01477 | −9.4 | −43.0 | 694 | NPACT01070 | −7.3 | −39.2 |
| 644 | NPACT00893 | −8.0 | −43.0 | 695 | NPACT00270 | −8.3 | −39.2 |

**Table S1**. *Continued.*

| No. | CompoundName/ Code | Docking Score(kcal/mol) | **MM-GBSA//MM** binding energy (kcal/mol) | No. | CompoundName/ Code | Docking Score(kcal/mol) | **MM-GBSA//MM** binding energy (kcal/mol) |
| --- | --- | --- | --- | --- | --- | --- | --- |
| 696 | NPACT01077 | −7.5 | −39.1 | 747 | NPACT00477 | −7.6 | −34.1 |
| 697 | NPACT00623 | −8.9 | −38.9 | 748 | NPACT00301 | −8.6 | −34.0 |
| 698 | NPACT01291 | −8.4 | −38.8 | 749 | NPACT01066 | −10.6 | −34.0 |
| 699 | NPACT01415 | −8.3 | −38.4 | 750 | NPACT00676 | −8.3 | −33.9 |
| 700 | NPACT00994 | −8.5 | −38.4 | 752 | NPACT00419 | −8.5 | −33.8 |
| 701 | NPACT01094 | −7.5 | −38.4 | 753 | NPACT01067 | −7.9 | −33.7 |
| 702 | NPACT00067 | −7.6 | −38.3 | 754 | NPACT01406 | −8.7 | −33.7 |
| 703 | NPACT01240 | −9.4 | −38.3 | 755 | NPACT00697 | −8.1 | −33.7 |
| 704 | NPACT01364 | −7.8 | −38.2 | 756 | NPACT00198 | −8.2 | −33.6 |
| 705 | NPACT00064 | −7.6 | −38.0 | 757 | NPACT00670 | −8.1 | −33.6 |
| 706 | NPACT01496 | −8.8 | −38.0 | 758 | NPACT00729 | −8.5 | −33.4 |
| 707 | NPACT01215 | −8.1 | −37.8 | 759 | NPACT01060 | −8.5 | −33.4 |
| 708 | NPACT01493 | −8.3 | −37.6 | 760 | NPACT01246 | −7.6 | −33.3 |
| 709 | NPACT01336 | −8.2 | −37.5 | 761 | NPACT00856 | −7.4 | −33.3 |
| 710 | NPACT00569 | −8.3 | −37.4 | 762 | NPACT01095 | −8.3 | −33.2 |
| 711 | NPACT01124 | −9.9 | −37.4 | 763 | NPACT00061 | −8.7 | −33.1 |
| 712 | NPACT00021 | −7.8 | −37.4 | 764 | NPACT00426 | −7.9 | −33.0 |
| 713 | NPACT00707 | −7.6 | −37.3 | 765 | NPACT01180 | −8.3 | −32.9 |
| 714 | NPACT00777 | −7.8 | −36.9 | 766 | NPACT01201 | −7.8 | −32.8 |
| 715 | NPACT01520 | −8.2 | −36.8 | 767 | NPACT00129 | −8.0 | −32.7 |
| 716 | NPACT01128 | −8.7 | −36.7 | 768 | NPACT00783 | −7.2 | −32.7 |
| 717 | NPACT00938 | −7.2 | −36.6 | 769 | NPACT00428 | −7.6 | −32.6 |
| 718 | NPACT00311 | −7.6 | −36.5 | 770 | NPACT01076 | −7.9 | −32.6 |
| 719 | NPACT01227 | −8.5 | −36.4 | 771 | NPACT01430 | −8.2 | −32.4 |
| 720 | NPACT00581 | −8.1 | −36.3 | 772 | NPACT01069 | −7.9 | −32.4 |
| 721 | NPACT01391 | −7.7 | −36.2 | 773 | NPACT01393 | −8.0 | −32.3 |
| 722 | NPACT00605 | −7.9 | −36.2 | 774 | NPACT01091 | −7.8 | −32.2 |
| 723 | NPACT00943 | −8.4 | −36.2 | 775 | NPACT00969 | −8.0 | −32.1 |
| 724 | NPACT00671 | −8.1 | −36.1 | 776 | NPACT01394 | −7.9 | −32.0 |
| 725 | NPACT01222 | −7.4 | −36.0 | 777 | NPACT00360 | −8.1 | −32.0 |
| 726 | NPACT00798 | −8.1 | −36.0 | 778 | NPACT00716 | −8.0 | −31.9 |
| 727 | NPACT00181 | −8.5 | −36.0 | 779 | NPACT01104 | −7.6 | −31.9 |
| 728 | NPACT01405 | −7.6 | −36.0 | 780 | NPACT00201 | −7.9 | −31.7 |
| 729 | NPACT00482 | −8.9 | −36.0 | 781 | NPACT00525 | −9.2 | −31.7 |
| 730 | NPACT01348 | −8.1 | −36.0 | 782 | NPACT00476 | −7.9 | −31.7 |
| 731 | NPACT01209 | −9.4 | −35.9 | 783 | NPACT00554 | −8.4 | −31.6 |
| 732 | NPACT00335 | −8.0 | −35.8 | 784 | NPACT00704 | −8.1 | −31.5 |
| 733 | NPACT00404 | −7.6 | −35.7 | 785 | NPACT01407 | −8.6 | −31.5 |
| 734 | NPACT00659 | −8.4 | −35.6 | 786 | NPACT01103 | −8.1 | −31.3 |
| 735 | NPACT01300 | −8.5 | −35.4 | 787 | NPACT01245 | −8.2 | −31.1 |
| 736 | NPACT01433 | −7.7 | −35.4 | 788 | NPACT01471 | −8.0 | −31.0 |
| 737 | NPACT00713 | −9.2 | −35.3 | 789 | NPACT01085 | −7.8 | −31.0 |
| 738 | NPACT01392 | −8.1 | −35.2 | 790 | NPACT01071 | −8.3 | −30.9 |
| 739 | NPACT00891 | −8.1 | −35.1 | 791 | NPACT01217 | −7.5 | −30.9 |
| 740 | NPACT01432 | −7.9 | −34.9 | 792 | NPACT00588 | −8.1 | −30.7 |
| 741 | NPACT01089 | −8.0 | −34.4 | 793 | NPACT00561 | −7.8 | −30.7 |
| 742 | NPACT01112 | −8.7 | −34.4 | 794 | NPACT00153 | −8.7 | −30.5 |
| 743 | NPACT00065 | −7.6 | −34.4 | 795 | NPACT00868 | −8.1 | −29.7 |
| 744 | NPACT01008 | −7.6 | −34.3 | 796 | NPACT01535 | −7.3 | −29.6 |
| 745 | NPACT00797 | −9.9 | −34.2 | 797 | NPACT00582 | −8.2 | −29.5 |
| 746 | NPACT01054 | −8.3 | −34.2 | 798 | NPACT01384 | −8.2 | −29.4 |

**Table S1**. *Continued.*

| No. | CompoundName/ Code | Docking Score(kcal/mol) | **MM-GBSA//MM** binding energy (kcal/mol) | No. | CompoundName/ Code | Docking Score(kcal/mol) | **MM-GBSA//MM** binding energy (kcal/mol) |  |
| --- | --- | --- | --- | --- | --- | --- | --- | --- |
| 799 | NPACT00002 | −7.6 | −29.1 | 807 | NPACT00847 | −7.7 | −22.4 |  |
| 800 | NPACT00006 | −7.3 | −28.8 | 808 | NPACT00849 | −7.8 | −22.4 |  |
| 801 | NPACT00857 | −8.0 | −27.2 | 809 | NPACT00848 | −8.4 | −22.3 |  |
| 802 | NPACT00805 | −7.1 | −23.3 | 810 | NPACT00269 | −9.7 | −22.3 |  |
| 803 | NPACT00877 | −8.7 | −22.7 | 811 | NPACT00268 | −11.2 | −22.3 |  |
| 804 | NPACT00745 | −7.6 | −22.5 | 812 | NPACT00379 | −10.1 | −22.2 |  |
| 805 | NPACT00696 | −7.9 | −22.5 | 813 | NPACT01464 | −8.2 | −22.2 |  |
| 806 | NPACT00489 | −7.9 | −22.5 | 814 | NPACT01214 | −9.0 | −22.1 |  |
| ***Steroids*** | | | | | | | | |
| 815 | NPACT00504 | −10.7 | −93.6 | 844 | NPACT00048 | −9.6 | −52.2 |  |
| 816 | NPACT00013 | −10.7 | −91.0 | 845 | NPACT00824 | −10.3 | −52.0 |  |
| 817 | NPACT00014 | −10.3 | −87.4 | 846 | NPACT00961 | −9.8 | −52.0 |  |
| 818 | NPACT00011 | −9.5 | −86.8 | 847 | NPACT01329 | −11.0 | −51.8 |  |
| 819 | NPACT00866 | −10.8 | −86.6 | 848 | NPACT00205 | −8.9 | −51.5 |  |
| 820 | NPACT01019 | −10.0 | −84.0 | 849 | NPACT00148 | −10.1 | −51.4 |  |
| 821 | NPACT00507 | −10.8 | −79.1 | 850 | NPACT00825 | −9.5 | −50.8 |  |
| 822 | NPACT00505 | −10.9 | −78.9 | 851 | NPACT01327 | −9.8 | −50.4 |  |
| 823 | NPACT00536 | −9.4 | −77.5 | 852 | NPACT01330 | −10.1 | −49.7 |  |
| 824 | NPACT00016 | −10.2 | −75.9 | 853 | NPACT01018 | −9.7 | −49.6 |  |
| 825 | NPACT00622 | −10.7 | −75.9 | 854 | NPACT00102 | −9.6 | −49.5 |  |
| 826 | NPACT00865 | −10.4 | −74.3 | 855 | NPACT01328 | −10.4 | −49.2 |  |
| 827 | NPACT00015 | −10.8 | −73.3 | 856 | NPACT01332 | −8.8 | −48.3 |  |
| 828 | NPACT00124 | −11.6 | −71.4 | 857 | NPACT00621 | −8.5 | −47.9 |  |
| 829 | NPACT00012 | −10.5 | −67.4 | 858 | NPACT01331 | −10.4 | −47.4 |  |
| 830 | NPACT00049 | −9.8 | −64.2 | 859 | NPACT00047 | −9.1 | −47.1 |  |
| 831 | NPACT00331 | −9.3 | −63.9 | 860 | NPACT00084 | −10.4 | −46.9 |  |
| 832 | NPACT00949 | −10.7 | −62.2 | 861 | NPACT01020 | −10.0 | −46.6 |  |
| 833 | NPACT00811 | −9.3 | −60.7 | 862 | NPACT01412 | −10.1 | −46.4 |  |
| 834 | NPACT01324 | −9.1 | −60.4 | 863 | NPACT00500 | −10.1 | −46.4 |  |
| 835 | NPACT00853 | −8.9 | −60.3 | 864 | NPACT01011 | −10.0 | −46.2 |  |
| 836 | NPACT00330 | −8.2 | −60.1 | 865 | NPACT01485 | −9.6 | −45.9 |  |
| 837 | NPACT01086 | −9.6 | −59.9 | 866 | NPACT00937 | −10.9 | −45.4 |  |
| 838 | NPACT00829 | −10.7 | −58.8 | 867 | NPACT00860 | −9.9 | −45.2 |  |
| 839 | NPACT00329 | −9.2 | −57.9 | 868 | NPACT00046 | −8.6 | −44.2 |  |
| 840 | NPACT00088 | −10.4 | −57.7 | 869 | NPACT00686 | −8.9 | −43.8 |  |
| 841 | NPACT00510 | −10.2 | −57.0 | 870 | NPACT01335 | −9.5 | −43.3 |  |
| 842 | NPACT00499 | −9.6 | −56.4 | 871 | NPACT01333 | −9.0 | −42.5 |  |
| 843 | NPACT00085 | −9.6 | −56.2 | 872 | NPACT00637 | −9.1 | −42.4 |  |
| 844 | NPACT01326 | −10.0 | −55.5 | 873 | NPACT00239 | −9.1 | −42.2 |  |
| 845 | NPACT00852 | −9.7 | −55.5 | 874 | NPACT00017 | −10.4 | −41.9 |  |
| 846 | NPACT01413 | −10.3 | −55.1 | 875 | NPACT00506 | −9.2 | −41.0 |  |
| 847 | NPACT00050 | −9.7 | −54.7 | 876 | NPACT00062 | −9.9 | −40.3 |  |
| 848 | NPACT01325 | −10.2 | −54.3 | 877 | NPACT00045 | −9.2 | −40.1 |  |
| 849 | NPACT00951 | −9.7 | −54.0 | 878 | NPACT00859 | −9.9 | −39.4 |  |
| 840 | NPACT00830 | −9.6 | −53.2 | 879 | NPACT00117 | −8.8 | −34.5 |  |
| 841 | NPACT01411 | −9.2 | −52.7 | 880 | NPACT01334 | −8.9 | −34.2 |  |
| 842 | NPACT00090 | −9.0 | −52.4 | 881 | NPACT00861 | −8.4 | −34.2 |  |
| 843 | NPACT00133 | −9.2 | −52.4 |  |  |  |  |  |
| ***Simple Aromatic Natural Products*** | | | | | | | | |
| 882 | NPACT00638 | −8.1 | −70.2 | 884 | NPACT00595 | −8.9 | −67.2 |  |
| 883 | NPACT00685 | −9.1 | −67.5 | 885 | NPACT00599 | −8.9 | −65.1 |  |

**Table S1**. *Continued.*

| No. | CompoundName/ Code | Docking Score(kcal/mol) | **MM-GBSA//MM** binding energy (kcal/mol) | No. | CompoundName/ Code | Docking Score(kcal/mol) | **MM-GBSA//MM** binding energy (kcal/mol) |  |
| --- | --- | --- | --- | --- | --- | --- | --- | --- |
| 886 | NPACT00593 | −8.6 | −63.6 | 924 | NPACT00547 | −9.5 | −47.4 |  |
| 887 | NPACT00940 | −10.6 | −62.4 | 925 | NPACT00747 | −10.0 | −47.0 |  |
| 888 | NPACT01212 | −9.8 | −60.5 | 926 | NPACT00750 | −9.9 | −46.9 |  |
| 889 | NPACT00598 | −8.1 | −60.1 | 927 | NPACT00748 | −9.6 | −46.4 |  |
| 890 | NPACT00001 | −9.0 | −59.3 | 928 | NPACT01570 | −10.0 | −46.2 |  |
| 891 | NPACT00007 | −8.3 | −59.2 | 929 | NPACT00987 | −8.7 | −44.6 |  |
| 892 | NPACT00003 | −9.6 | −59.1 | 930 | NPACT00261 | −9.4 | −44.4 |  |
| 893 | NPACT00639 | −8.6 | −58.2 | 931 | NPACT00490 | −10.6 | −43.4 |  |
| 894 | NPACT00473 | −9.0 | −57.1 | 932 | NPACT00896 | −7.2 | −41.6 |  |
| 895 | NPACT01395 | −9.2 | −56.8 | 933 | NPACT01451 | −8.3 | −40.0 |  |
| 896 | NPACT00601 | −10.0 | −56.2 | 934 | NPACT00187 | −7.0 | −39.1 |  |
| 897 | NPACT00202 | −9.2 | −56.0 | 935 | NPACT00629 | −6.9 | −39.0 |  |
| 898 | NPACT01023 | −9.2 | −55.4 | 936 | NPACT00025 | −7.5 | −38.5 |  |
| 899 | NPACT00596 | −9.0 | −55.0 | 937 | NPACT00152 | −8.3 | −38.2 |  |
| 900 | NPACT00594 | −9.6 | −54.8 | 938 | NPACT00161 | −6.8 | −37.3 |  |
| 901 | NPACT00939 | −10.4 | −53.7 | 939 | NPACT00289 | −9.6 | −36.1 |  |
| 902 | NPACT00597 | −8.9 | −53.7 | 940 | NPACT00905 | −7.6 | −35.8 |  |
| 903 | NPACT00752 | −9.5 | −52.7 | 941 | NPACT01090 | −7.7 | −34.6 |  |
| 904 | NPACT01343 | −8.3 | −52.7 | 942 | NPACT01294 | −7.6 | −34.1 |  |
| 905 | NPACT00986 | −10.0 | −52.5 | 943 | NPACT01439 | −7.6 | −33.5 |  |
| 906 | NPACT00602 | −9.1 | −52.2 | 944 | NPACT01396 | −7.8 | −32.7 |  |
| 907 | NPACT00456 | −9.0 | −51.8 | 945 | NPACT01218 | −6.2 | −32.7 |  |
| 908 | NPACT01542 | −8.1 | −51.5 | 946 | NPACT00869 | −7.7 | −32.6 |  |
| 909 | NPACT01480 | −8.8 | −51.4 | 947 | NPACT00572 | −7.5 | −28.2 |  |
| 910 | NPACT00471 | −11.1 | −51.4 | 948 | NPACT01079 | −5.9 | −28.0 |  |
| 911 | NPACT00751 | −8.9 | −51.3 | 949 | NPACT00976 | −6.5 | −26.3 |  |
| 912 | NPACT00027 | −9.5 | −50.8 | 950 | NPACT00687 | −6.2 | −26.2 |  |
| 913 | NPACT01305 | −9.2 | −50.6 | 951 | NPACT00591 | −6.1 | −26.0 |  |
| 914 | NPACT00034 | −8.9 | −49.9 | 952 | NPACT00881 | −6.0 | −24.9 |  |
| 915 | NPACT00020 | −7.4 | −49.7 | 953 | NPACT00132 | −6.2 | −23.4 |  |
| 916 | NPACT01507 | −9.3 | −49.4 | 954 | NPACT00862 | −5.9 | −19.0 |  |
| 917 | NPACT00749 | −9.4 | −49.3 | 955 | NPACT00315 | −5.8 | −18.8 |  |
| 918 | NPACT00633 | −9.6 | −49.1 | 956 | NPACT00423 | −6.4 | −16.6 |  |
| 919 | NPACT00237 | −9.2 | −49.0 | 957 | NPACT00314 | −5.2 | −14.8 |  |
| 920 | NPACT00068 | −7.6 | −49.0 | 958 | NPACT01293 | −5.0 | −14.1 |  |
| 921 | NPACT00326 | −8.7 | −48.1 | 959 | NPACT01501 | −5.7 | −14.1 |  |
| 922 | NPACT01398 | −10.1 | −47.8 | 960 | NPACT01042 | −6.5 | −14.1 |  |
| 923 | NPACT01375 | −8.7 | −47.5 | 961 | NPACT00985 | −5.4 | −14.1 |  |
| ***Polyketides*** | | | | | | | | |
| 962 | NPACT00262 | −8.3 | −96.8 | 974 | NPACT00357 | −8.4 | −88.2 |  |
| 963 | NPACT00889 | −8.3 | −96.8 | 975 | NPACT00672 | −8.3 | −88.2 |  |
| 964 | NPACT00292 | −8.0 | −92.4 | 976 | NPACT01279 | −8.0 | −87.8 |  |
| 965 | NPACT00496 | −8.3 | −91.6 | 977 | NPACT00782 | −7.9 | −87.1 |  |
| 966 | NPACT00624 | −8.1 | −90.6 | 978 | NPACT00926 | −7.7 | −87.0 |  |
| 967 | NPACT01506 | −8.3 | −90.3 | 979 | NPACT00294 | −8.1 | −86.8 |  |
| 968 | NPACT00960 | −8.3 | −89.9 | 980 | NPACT00033 | −8.1 | −86.8 |  |
| 969 | NPACT00297 | −7.5 | −89.7 | 981 | NPACT00437 | −7.7 | −86.7 |  |
| 970 | NPACT00625 | −8.3 | −89.6 | 982 | NPACT00263 | −8.2 | −86.5 |  |
| 971 | NPACT00010 | −7.7 | −89.4 | 983 | NPACT00369 | −8.3 | −86.3 |  |
| 972 | NPACT00721 | −8.4 | −88.5 | 984 | NPACT00925 | −7.6 | −85.8 |  |
| 973 | NPACT00788 | −8.1 | −88.3 | 985 | NPACT00355 | −8.3 | −85.7 |  |

**Table S1**. *Continued.*

| **No.** | **Compound**  **Name/ Code** | **Docking Score**  **(kcal/mol)** | **MM-GBSA//MM**  **binding energy (kcal/mol)** | **No.** | **Compound**  **Name/ Code** | **Docking Score**  **(kcal/mol)** | **MM-GBSA//MM**  **binding energy (kcal/mol)** |
| --- | --- | --- | --- | --- | --- | --- | --- |
| 986 | NPACT00370 | −7.9 | −85.3 | 1019 | NPACT00439 | −7.6 | −79.9 |
| 987 | NPACT00073 | −7.6 | −85.2 | 1020 | NPACT00264 | −7.8 | −79.7 |
| 988 | NPACT00781 | −7.7 | −85.2 | 1021 | NPACT01276 | −8.1 | −79.7 |
| 989 | NPACT00981 | −7.8 | −84.6 | 1022 | NPACT00785 | −8.2 | −79.5 |
| 990 | NPACT00394 | −7.5 | −84.5 | 1023 | NPACT00296 | −7.7 | −78.8 |
| 991 | NPACT00371 | −8.5 | −84.4 | 1024 | NPACT00009 | −8.2 | −78.7 |
| 992 | NPACT00923 | −7.9 | −84.4 | 1025 | NPACT00035 | −8.4 | −78.5 |
| 993 | NPACT00266 | −8.0 | −84.1 | 1026 | NPACT01277 | −7.5 | −78.4 |
| 994 | NPACT00614 | −7.8 | −84.0 | 1027 | NPACT01263 | −8.2 | −78.3 |
| 995 | NPACT00087 | −8.3 | −83.7 | 1028 | NPACT01511 | −8.0 | −78.2 |
| 996 | NPACT00395 | −8.0 | −83.7 | 1029 | NPACT00724 | −8.2 | −78.1 |
| 997 | NPACT00159 | −8.5 | −83.6 | 1030 | NPACT00786 | −7.9 | −77.8 |
| 998 | NPACT00032 | −8.5 | −83.3 | 1031 | NPACT00356 | −8.4 | −77.6 |
| 999 | NPACT01278 | −8.4 | −83.2 | 1032 | NPACT00031 | −7.9 | −77.5 |
| 1000 | NPACT00952 | −8.3 | −82.9 | 1033 | NPACT01470 | −8.2 | −77.3 |
| 1001 | NPACT01153 | −7.7 | −82.7 | 1034 | NPACT01489 | −8.1 | −77.1 |
| 1002 | NPACT01152 | −7.4 | −82.7 | 1035 | NPACT00265 | −7.6 | −77.1 |
| 1003 | NPACT01002 | −7.7 | −82.7 | 1036 | NPACT00438 | −7.8 | −76.8 |
| 1004 | NPACT00957 | −7.9 | −81.9 | 1037 | NPACT01178 | −8.1 | −76.8 |
| 1005 | NPACT00635 | −8.1 | −81.8 | 1038 | NPACT00267 | −7.4 | −75.3 |
| 1006 | NPACT00959 | −8.1 | −81.6 | 1039 | NPACT00723 | −8.4 | −75.2 |
| 1007 | NPACT00158 | −8.5 | −81.6 | 1040 | NPACT01157 | −9.8 | −74.5 |
| 1008 | NPACT01130 | −8.1 | −81.5 | 1041 | NPACT00722 | −8.0 | −73.7 |
| 1009 | NPACT00956 | −8.3 | −81.3 | 1042 | NPACT00293 | −8.0 | −73.6 |
| 1010 | NPACT00157 | −8.0 | −81.3 | 1043 | NPACT00780 | −7.9 | −73.3 |
| 1011 | NPACT00396 | −7.7 | −81.0 | 1044 | NPACT01177 | −7.4 | −71.3 |
| 1012 | NPACT00368 | −7.9 | −81.0 | 1045 | NPACT01280 | −8.0 | −69.3 |
| 1013 | NPACT00890 | −8.3 | −80.9 | 1046 | NPACT00367 | −9.0 | −63.8 |
| 1014 | NPACT00955 | −8.1 | −80.9 | 1047 | NPACT01275 | −7.8 | −61.3 |
| 1015 | NPACT00295 | −8.0 | −80.7 | 1048 | NPACT01288 | −7.5 | −59.0 |
| 1016 | NPACT00634 | −8.0 | −80.6 | 1049 | NPACT00888 | −6.8 | −54.1 |
| 1017 | NPACT00787 | −7.7 | −80.3 | 1050 | NPACT01285 | −7.2 | −50.3 |
| 1018 | NPACT01252 | −7.6 | −80.2 |  |  |  |  |
| ***Polycyclic Aromatic Natural Products*** | | | | | | | |
| 1051 | NPACT00910 | −9.3 | −63.0 | 1068 | NPACT00989 | −9.5 | −49.8 |
| 1052 | NPACT00214 | −8.0 | −60.1 | 1069 | NPACT00914 | −9.2 | −48.7 |
| 1053 | NPACT00915 | −9.8 | −59.5 | 1070 | NPACT01427 | −10.3 | −48.5 |
| 1054 | NPACT00248 | −8.0 | −59.0 | 1071 | NPACT00255 | −8.1 | −48.2 |
| 1055 | NPACT01208 | −10.5 | −58.3 | 1072 | NPACT00252 | −10.0 | −47.7 |
| 1056 | NPACT00911 | −8.9 | −57.8 | 1073 | NPACT00254 | −9.3 | −46.9 |
| 1057 | NPACT00213 | −8.3 | −57.4 | 1074 | NPACT01376 | −9.8 | −44.6 |
| 1058 | NPACT00916 | −9.0 | −55.9 | 1075 | NPACT00912 | −8.5 | −44.3 |
| 1059 | NPACT00249 | −10.0 | −54.8 | 1076 | NPACT00516 | −8.9 | −44.3 |
| 1060 | NPACT00247 | −8.5 | −54.0 | 1077 | NPACT01377 | −10.7 | −41.8 |
| 1061 | NPACT01536 | −8.3 | −53.4 | 1078 | NPACT00313 | −8.0 | −41.8 |
| 1062 | NPACT01219 | −10.2 | −53.4 | 1079 | NPACT00909 | −9.7 | −41.7 |
| 1063 | NPACT00913 | −8.8 | −52.9 | 1080 | NPACT00251 | −8.7 | −41.3 |
| 1064 | NPACT00253 | −10.6 | −51.5 | 1081 | NPACT00754 | −9.3 | −40.8 |
| 1065 | NPACT00250 | −8.1 | −50.8 | 1082 | NPACT00755 | −8.5 | −40.1 |
| 1066 | NPACT00908 | −8.8 | −50.7 | 1083 | NPACT00246 | −8.9 | −39.6 |
| 1067 | NPACT00907 | −10.8 | −50.3 | 1084 | NPACT01516 | −9.0 | −38.9 |

**Table S1**. *Continued.*

| No. | **Compound** Name/ Code | Docking Score(kcal/mol) | **MM-GBSA//MM** binding energy (kcal/mol) | No. | **Compound** Name/ Code | Docking Score(kcal/mol) | **MM-GBSA//MM** binding energy (kcal/mol) |
| --- | --- | --- | --- | --- | --- | --- | --- |
| 1085 | NPACT01304 | −8.9 | −37.8 | 1099 | NPACT01073 | −8.9 | −31.9 |
| 1086 | NPACT00756 | −8.7 | −37.3 | 1100 | NPACT00906 | −8.3 | −31.7 |
| 1087 | NPACT01421 | −10.2 | −37.0 | 1101 | NPACT00420 | −8.8 | −31.2 |
| 1088 | NPACT00775 | −8.4 | −36.7 | 1102 | NPACT00502 | −8.6 | −31.2 |
| 1089 | NPACT01064 | −8.4 | −34.4 | 1103 | NPACT00211 | −8.3 | −30.4 |
| 1090 | NPACT01187 | −9.0 | −34.4 | 1104 | NPACT01074 | −8.4 | −30.3 |
| 1091 | NPACT01097 | −8.4 | −34.2 | 1105 | NPACT00870 | −7.6 | −30.0 |
| 1092 | NPACT00917 | −9.0 | −33.9 | 1106 | NPACT00175 | −7.4 | −29.6 |
| 1093 | NPACT01063 | −8.5 | −33.7 | 1107 | NPACT00587 | −8.0 | −29.4 |
| 1094 | NPACT01122 | −8.7 | −33.5 | 1108 | NPACT01383 | −8.3 | −28.6 |
| 1095 | NPACT00325 | −8.9 | −33.3 | 1109 | NPACT00551 | −8.5 | −28.5 |
| 1096 | NPACT00231 | −8.5 | −33.0 | 1110 | NPACT00965 | −7.5 | −27.8 |
| 1097 | NPACT01062 | −8.1 | −32.9 | 1111 | NPACT00168 | −6.9 | −24.1 |
| 1098 | NPACT00885 | −8.4 | −32.7 | 1112 | NPACT01234 | −6.5 | −20.2 |
| ***Alkaloids*** | | | | | | | |
| 1113 | NPACT00241 | −11.3 | −88.3 | 1149 | NPACT01161 | −9.4 | −46.6 |
| 1114 | NPACT00488 | −9.8 | −87.5 | 1150 | NPACT00903 | −9.8 | −46.6 |
| 1115 | NPACT00177 | −10.0 | −70.2 | 1151 | NPACT00979 | −10.8 | −46.5 |
| 1116 | NPACT00130 | −9.6 | −68.9 | 1152 | NPACT00741 | −9.6 | −46.5 |
| 1117 | NPACT00131 | −10.1 | −68.5 | 1153 | NPACT01423 | −9.5 | −46.4 |
| 1118 | NPACT01005 | −7.4 | −67.1 | 1154 | NPACT01360 | −9.3 | −46.3 |
| 1119 | NPACT00186 | −10.2 | −65.1 | 1155 | NPACT00980 | −9.9 | −46.3 |
| 1120 | NPACT00109 | −9.9 | −62.8 | 1156 | NPACT01359 | −8.5 | −45.8 |
| 1121 | NPACT01007 | −7.7 | −61.3 | 1157 | NPACT01472 | −8.6 | −45.1 |
| 1122 | NPACT01302 | −9.0 | −60.5 | 1158 | NPACT00347 | −8.9 | −45.1 |
| 1123 | NPACT00523 | −7.6 | −59.9 | 1159 | NPACT01204 | −9.9 | −45.0 |
| 1124 | NPACT00522 | −8.2 | −59.3 | 1160 | NPACT01108 | −9.8 | −44.9 |
| 1125 | NPACT00327 | −8.0 | −58.3 | 1161 | NPACT01483 | −10.0 | −44.7 |
| 1126 | NPACT01565 | −10.0 | −57.5 | 1162 | NPACT01052 | −9.4 | −44.5 |
| 1127 | NPACT01442 | −6.9 | −56.8 | 1163 | NPACT01238 | −7.6 | −44.5 |
| 1128 | NPACT01181 | −9.0 | −56.6 | 1164 | NPACT00794 | −7.6 | −44.5 |
| 1129 | NPACT01357 | −10.4 | −56.5 | 1165 | NPACT01358 | −8.5 | −44.0 |
| 1130 | NPACT00664 | −8.1 | −55.2 | 1166 | NPACT01051 | −9.8 | −43.9 |
| 1131 | NPACT01478 | −9.8 | −54.4 | 1167 | NPACT01044 | −9.3 | −43.7 |
| 1132 | NPACT01163 | −7.7 | −53.8 | 1168 | NPACT01317 | −8.0 | −42.9 |
| 1133 | NPACT01053 | −9.4 | −53.7 | 1169 | NPACT00740 | −9.9 | −42.7 |
| 1134 | NPACT00318 | −9.6 | −53.7 | 1170 | NPACT01055 | −8.3 | −42.6 |
| 1135 | NPACT01228 | −9.6 | −53.6 | 1171 | NPACT00842 | −8.1 | −42.0 |
| 1136 | NPACT01166 | −10.1 | −52.8 | 1172 | NPACT01242 | −9.3 | −41.4 |
| 1137 | NPACT01491 | −8.6 | −52.3 | 1173 | NPACT00919 | −7.3 | −41.1 |
| 1138 | NPACT00346 | −8.7 | −50.7 | 1174 | NPACT00816 | −7.2 | −40.9 |
| 1139 | NPACT01362 | −9.4 | −50.4 | 1175 | NPACT01096 | −5.7 | −40.8 |
| 1140 | NPACT01484 | −9.7 | −50.3 | 1176 | NPACT00935 | −10.7 | −40.6 |
| 1141 | NPACT01006 | −7.6 | −50.0 | 1177 | NPACT01361 | −9.6 | −40.6 |
| 1142 | NPACT00819 | −7.7 | −48.5 | 1178 | NPACT00417 | −10.0 | −40.3 |
| 1143 | NPACT00319 | −9.6 | −48.4 | 1179 | NPACT00389 | −10.1 | −40.1 |
| 1144 | NPACT01229 | −9.1 | −48.1 | 1180 | NPACT01179 | −7.8 | −40.0 |
| 1145 | NPACT01497 | −9.0 | −47.6 | 1181 | NPACT01397 | −9.7 | −39.3 |
| 1146 | NPACT00739 | −9.4 | −47.6 | 1182 | NPACT00760 | −7.2 | −39.2 |
| 1147 | NPACT01401 | −9.7 | −47.5 | 1183 | NPACT00575 | −8.5 | −38.7 |
| 1148 | NPACT00972 | −9.7 | −46.8 | 1184 | NPACT00962 | −9.3 | −38.2 |

**Table S1**. *Continued.*

| No. | **Compound** Name/ Code | Docking Score(kcal/mol) | **MM-GBSA//MM** binding energy (kcal/mol) | No. | **Compound** Name/ Code | Docking Score(kcal/mol) | | **MM-GBSA//MM** binding energy (kcal/mol) |
| --- | --- | --- | --- | --- | --- | --- | --- | --- |
| 1185 | NPACT01059 | −7.5 | −37.6 | 1201 | NPACT00643 | −7.2 | −33.1 | |
| 1186 | NPACT00683 | −8.8 | −37.1 | 1202 | NPACT00733 | −8.0 | −33.0 | |
| 1187 | NPACT01428 | −8.0 | −37.0 | 1203 | NPACT00867 | −7.9 | −32.1 | |
| 1188 | NPACT01230 | −9.5 | −36.8 | 1204 | NPACT00732 | −8.6 | −31.6 | |
| 1189 | NPACT00666 | −8.2 | −36.2 | 1205 | NPACT01437 | −7.8 | −31.1 | |
| 1190 | NPACT01110 | −8.8 | −36.2 | 1206 | NPACT01144 | −7.5 | −31.0 | |
| 1191 | NPACT01251 | −9.5 | −35.9 | 1207 | NPACT00753 | −8.8 | −30.6 | |
| 1192 | NPACT01035 | −9.8 | −35.5 | 1208 | NPACT01165 | −7.5 | −30.4 | |
| 1193 | NPACT00430 | −6.4 | −35.5 | 1209 | NPACT01461 | −8.3 | −30.0 | |
| 1194 | NPACT01482 | −8.4 | −35.3 | 1210 | NPACT00445 | −8.3 | −29.4 | |
| 1195 | NPACT01438 | −7.8 | −34.9 | 1211 | NPACT01160 | −8.3 | −28.6 | |
| 1196 | NPACT00806 | −8.7 | −34.8 | 1212 | NPACT00640 | −7.2 | −28.4 | |
| 1197 | NPACT01424 | −7.8 | −34.3 | 1213 | NPACT01164 | −7.8 | −27.9 | |
| 1198 | NPACT01109 | −8.6 | −33.7 | 1214 | NPACT00641 | −7.5 | −22.8 | |
| 1199 | NPACT01426 | −8.2 | −33.5 | 1215 | NPACT00642 | −7.4 | −21.8 | |
| 1200 | NPACT01195 | −9.6 | −33.4 | 1216 | NPACT00644 | −7.5 | −21.2 | |
| ***Lignans*** | | | | | | | | |
| 1217 | NPACT00501 | −11.1 | −87.9 | 1251 | NPACT01500 | −8.1 | −44.6 | |
| 1218 | NPACT00845 | −9.1 | −74.9 | 1252 | NPACT00776 | −8.4 | −44.3 | |
| 1219 | NPACT01250 | −9.0 | −74.4 | 1253 | NPACT00709 | −9.0 | −44.2 | |
| 1220 | NPACT00274 | −9.5 | −68.0 | 1254 | NPACT00803 | −8.0 | −44.1 | |
| 1221 | NPACT01027 | −7.7 | −58.5 | 1255 | NPACT00221 | −9.4 | −43.6 | |
| 1222 | NPACT00272 | −10.5 | −57.6 | 1256 | NPACT00970 | −7.9 | −43.4 | |
| 1223 | NPACT00220 | −8.8 | −55.1 | 1257 | NPACT00518 | −7.9 | −43.3 | |
| 1224 | NPACT00520 | −8.6 | −53.3 | 1258 | NPACT01191 | −7.3 | −42.6 | |
| 1225 | NPACT00224 | −7.8 | −53.2 | 1259 | NPACT01258 | −7.7 | −42.1 | |
| 1226 | NPACT00555 | −8.2 | −52.5 | 1260 | NPACT01167 | −8.1 | −42.1 | |
| 1227 | NPACT00736 | −8.8 | −51.4 | 1261 | NPACT01143 | −8.4 | −42.0 | |
| 1228 | NPACT01036 | −8.6 | −51.3 | 1262 | NPACT00684 | −7.5 | −41.9 | |
| 1229 | NPACT00005 | −7.3 | −49.7 | 1263 | NPACT01226 | −9.2 | −41.8 | |
| 1230 | NPACT00550 | −7.2 | −49.6 | 1264 | NPACT00735 | −8.8 | −41.6 | |
| 1231 | NPACT00742 | −9.2 | −48.9 | 1265 | NPACT00821 | −9.0 | −41.5 | |
| 1232 | NPACT00771 | −8.1 | −48.8 | 1266 | NPACT00328 | −8.2 | −41.4 | |
| 1233 | NPACT00802 | −8.1 | −48.7 | 1267 | NPACT00759 | −7.9 | −41.3 | |
| 1234 | NPACT00538 | −8.7 | −48.3 | 1268 | NPACT00855 | −8.7 | −41.0 | |
| 1235 | NPACT00475 | −8.4 | −48.2 | 1269 | NPACT00218 | −8.6 | −40.7 | |
| 1236 | NPACT00156 | −8.4 | −48.0 | 1270 | NPACT00995 | −8.6 | −40.6 | |
| 1237 | NPACT00864 | −8.4 | −47.9 | 1271 | NPACT00675 | −7.6 | −40.5 | |
| 1238 | NPACT00190 | −8.8 | −47.7 | 1272 | NPACT01045 | −8.5 | −40.5 | |
| 1239 | NPACT00590 | −8.2 | −47.6 | 1272 | NPACT00706 | −8.0 | −39.9 | |
| 1240 | NPACT00820 | −7.7 | −47.5 | 1273 | NPACT01454 | −7.8 | −39.8 | |
| 1241 | NPACT00873 | −8.9 | −46.3 | 1274 | NPACT00563 | −8.5 | −39.5 | |
| 1242 | NPACT00358 | −8.9 | −46.0 | 1275 | NPACT01463 | −8.0 | −39.4 | |
| 1243 | NPACT00057 | −7.9 | −45.9 | 1276 | NPACT00154 | −8.6 | −39.4 | |
| 1244 | NPACT00658 | −7.4 | −45.8 | 1277 | NPACT00495 | −8.1 | −39.4 | |
| 1245 | NPACT00872 | −8.3 | −45.3 | 1278 | NPACT01417 | −8.2 | −39.3 | |
| 1246 | NPACT00273 | −8.4 | −45.2 | 1279 | NPACT00822 | −8.1 | −39.2 | |
| 1247 | NPACT00556 | −7.6 | −45.2 | 1280 | NPACT01050 | −7.7 | −39.1 | |
| 1248 | NPACT00945 | −9.0 | −45.0 | 1281 | NPACT00734 | −9.1 | −38.9 | |
| 1249 | NPACT01282 | −8.8 | −44.8 | 1282 | NPACT00818 | −7.9 | −37.8 | |
| 1250 | NPACT01342 | −9.4 | −44.6 | 1283 | NPACT00512 | −8.5 | −37.7 | |

**Table S1**. *Continued.*

| No. | **Compound** Name/ Code | Docking Score(kcal/mol) | **MM-GBSA//MM** binding energy (kcal/mol) | No. | **Compound** Name/ Code | Docking Score(kcal/mol) | **MM-GBSA//MM** binding energy (kcal/mol) |  |
| --- | --- | --- | --- | --- | --- | --- | --- | --- |
| 1284 | NPACT00589 | −9.1 | −37.6 | 1294 | NPACT01341 | −9.5 | −34.3 |  |
| 1285 | NPACT01498 | −8.2 | −37.6 | 1295 | NPACT01223 | −9.2 | −33.1 |  |
| 1286 | NPACT00854 | −8.8 | −37.3 | 1296 | NPACT01571 | −8.1 | −32.5 |  |
| 1287 | NPACT01081 | −7.2 | −37.0 | 1297 | NPACT00665 | −8.3 | −31.8 |  |
| 1288 | NPACT00770 | −7.6 | −37.0 | 1298 | NPACT01316 | −8.0 | −31.2 |  |
| 1289 | NPACT00167 | −8.3 | −36.8 | 1299 | NPACT00580 | −6.6 | −29.4 |  |
| 1290 | NPACT00290 | −8.9 | −36.5 | 1300 | NPACT00366 | −6.2 | −28.5 |  |
| 1291 | NPACT01410 | −9.8 | −35.6 | 1301 | NPACT01416 | −8.1 | −27.6 |  |
| 1292 | NPACT00155 | −8.2 | −35.5 | 1302 | NPACT01264 | −6.2 | −27.3 |  |
| 1293 | NPACT00008 | −8.0 | −35.0 | 1303 | NPACT01420 | −8.1 | −26.9 |  |
| ***Aliphatic Natural Products*** | | | | | | | | |
| 1304 | NPACT01545 | −10.8 | −132.2 | 1314 | NPACT00024 | −6.1 | −46.4 |  |
| 1305 | NPACT01533 | −8.5 | −102.5 | 1315 | NPACT00574 | −7.1 | −46.4 |  |
| 1306 | NPACT00228 | −7.8 | −56.4 | 1316 | NPACT00486 | −7.0 | −44.4 |  |
| 1307 | NPACT00227 | −7.5 | −53.5 | 1317 | NPACT00513 | −6.5 | −44.2 |  |
| 1308 | NPACT00226 | −7.2 | −52.8 | 1318 | NPACT00023 | −5.9 | −43.0 |  |
| 1309 | NPACT00773 | −6.6 | −48.9 | 1319 | NPACT00573 | −6.9 | −42.2 |  |
| 1310 | NPACT01510 | −7.3 | −48.6 | 1320 | NPACT00022 | −6.0 | −41.0 |  |
| 1311 | NPACT00485 | −6.8 | −48.3 | 1321 | NPACT00840 | −6.3 | −39.2 |  |
| 1312 | NPACT00493 | −6.4 | −48.3 | 1322 | NPACT00060 | −4.8 | −23.2 |  |
| 1313 | NPACT00492 | −6.7 | −47.1 | 1323 | NPACT01319 | −4.0 | −17.9 |  |
| ***Amino acids and Peptides*** | | | | | | | | |
| 1324 | NPACT01028 | −9.0 | −44.2 | 1326 | NPACT00932 | −4.7 | −23.6 |  |
| 1325 | NPACT00920 | −7.8 | −39.6 |  |  |  |  |  |
| ***Benzofuranoids*** | | | | | | | | |
| 1327 | NPACT00674 | −10.6 | −63.7 | 1336 | NPACT01273 | −9.2 | −53.2 |  |
| 1328 | NPACT00472 | −9.9 | −61.7 | 1337 | NPACT01268 | −8.7 | −49.5 |  |
| 1329 | NPACT00944 | −8.0 | −61.2 | 1338 | NPACT01271 | −10.3 | −49.2 |  |
| 1330 | NPACT00876 | −8.1 | −57.0 | 1339 | NPACT01039 | −8.3 | −47.1 |  |
| 1331 | NPACT00583 | −10.0 | −56.4 | 1340 | NPACT01270 | −9.1 | −46.7 |  |
| 1332 | NPACT00711 | −8.6 | −55.7 | 1341 | NPACT00921 | −8.2 | −43.0 |  |
| 1333 | NPACT01274 | −9.5 | −55.7 | 1342 | NPACT00188 | −6.9 | −34.4 |  |
| 1334 | NPACT01269 | −10.1 | −53.9 | 1343 | NPACT00191 | −6.9 | −33.2 |  |
| 1335 | NPACT01272 | −9.8 | −53.6 | 1344 | NPACT00570 | −7.8 | −30.8 |  |
| ***Benzopyranoids*** | | | | | | | | |
| 1345 | NPACT01544 | −8.3 | −60.1 | 1357 | NPACT01012 | −8.3 | −37.7 |  |
| 1346 | NPACT00149 | −10.5 | −55.8 | 1358 | NPACT01162 | −8.6 | −36.9 |  |
| 1347 | NPACT00163 | −10.0 | −55.0 | 1359 | NPACT00491 | −7.7 | −34.0 |  |
| 1348 | NPACT01297 | −10.0 | −52.8 | 1360 | NPACT00838 | −8.1 | −33.4 |  |
| 1349 | NPACT00576 | −10.1 | −50.5 | 1361 | NPACT00526 | −7.6 | −33.2 |  |
| 1350 | NPACT00180 | −8.0 | −50.1 | 1362 | NPACT00521 | −8.7 | −31.1 |  |
| 1351 | NPACT01435 | −8.9 | −46.9 | 1363 | NPACT01101 | −6.9 | −30.9 |  |
| 1352 | NPACT00179 | −8.1 | −44.4 | 1364 | NPACT00941 | −7.3 | −30.6 |  |
| 1353 | NPACT01154 | −9.2 | −43.5 | 1365 | NPACT00178 | −7.4 | −27.2 |  |
| 1354 | NPACT01374 | −9.6 | −43.0 | 1366 | NPACT00678 | −7.9 | −27.0 |  |
| 1355 | NPACT01296 | −9.0 | −41.3 | 1367 | NPACT00192 | −7.0 | −26.9 |  |
| 1356 | NPACT00300 | −8.7 | −41.1 | 1368 | NPACT00882 | −7.2 | −25.8 |  |
| ***Saponin*** | | | | | | | | |
| 1369 | NPACT00144 | −10.0 | −114.3 | 1371 | NPACT01560 | −10.0 | −113.2 |  |
| 1370 | NPACT00079 | −9.5 | −113.3 | 1372 | NPACT00618 | −9.7 | −111.6 |  |

**Table S1**. *Continued.*

| No. | **Compound** Name/ Code | Docking Score(kcal/mol) | **MM-GBSA//MM** binding energy (kcal/mol) | No. | **Compound** Name/ Code | Docking Score(kcal/mol) | **MM-GBSA//MM** binding energy (kcal/mol) |
| --- | --- | --- | --- | --- | --- | --- | --- |
| 1373 | NPACT01548 | −9.9 | −109.3 | 1396 | NPACT00836 | −10.9 | −82.9 |
| 1374 | NPACT01558 | −10.6 | −109.1 | 1397 | NPACT00720 | −6.0 | −81.0 |
| 1375 | NPACT01561 | −10.5 | −107.8 | 1398 | NPACT00875 | −11.1 | −80.1 |
| 1376 | NPACT01564 | −9.6 | −107.5 | 1399 | NPACT00083 | −10.2 | −79.9 |
| 1378 | NPACT00737 | −10.1 | −100.7 | 1400 | NPACT00619 | −10.1 | −79.1 |
| 1379 | NPACT00134 | −11.8 | −99.7 | 1401 | NPACT00082 | −9.4 | −78.9 |
| 1380 | NPACT01562 | −9.8 | −99.6 | 1402 | NPACT00718 | −6.3 | −75.3 |
| 1381 | NPACT01559 | −10.7 | −99.2 | 1403 | NPACT00615 | −9.9 | −74.3 |
| 1382 | NPACT00081 | −10.0 | −98.2 | 1404 | NPACT00654 | −11.7 | −73.5 |
| 1383 | NPACT00835 | −11.4 | −96.8 | 1405 | NPACT00078 | −9.2 | −72.7 |
| 1384 | NPACT00119 | −9.6 | −96.1 | 1406 | NPACT01563 | −9.7 | −71.8 |
| 1385 | NPACT00646 | −9.6 | −94.9 | 1407 | NPACT00120 | −10.5 | −71.0 |
| 1386 | NPACT00657 | −9.8 | −93.5 | 1408 | NPACT01547 | −9.3 | −70.2 |
| 1387 | NPACT00653 | −10.9 | −92.1 | 1409 | NPACT00656 | −11.3 | −69.5 |
| 1388 | NPACT00616 | −10.4 | −88.8 | 1410 | NPACT00080 | −9.8 | −68.8 |
| 1389 | NPACT01232 | −10.2 | −87.7 | 1411 | NPACT00620 | −9.5 | −67.7 |
| 1390 | NPACT00145 | −9.9 | −87.1 | 1412 | NPACT00142 | −9.4 | −67.7 |
| 1391 | NPACT01550 | −9.8 | −85.4 | 1413 | NPACT01546 | −10.4 | −66.1 |
| 1392 | NPACT00655 | −10.1 | −85.3 | 1414 | NPACT00322 | −9.8 | −60.5 |
| 1393 | NPACT01556 | −9.2 | −85.0 | 1415 | NPACT01192 | −10.1 | −44.6 |
| 1394 | NPACT00143 | −9.3 | −84.8 | 1416 | NPACT00719 | −4.1 | −41.3 |
| 1395 | NPACT00645 | −9.3 | −82.9 | 1417 | NPACT00717 | −5.4 | −30.3 |
| ***Tannins*** | | | | | | | |
| 1418 | NPACT00968 | −12.0 | −150.3 | 1421 | NPACT01168 | −8.2 | −45.9 |
| 1419 | NPACT00416 | −9.4 | −64.6 | 1422 | NPACT00320 | −7.5 | −36.5 |
| 1420 | NPACT00063 | −8.4 | −51.1 | 1423 | NPACT00567 | −6.1 | −16.8 |
| ***Oxygen Heterocycles*** | | | | | | | |
| 1424 | NPACT00217 | −8.7 | −60.7 | 1430 | NPACT00810 | −10.0 | −35.5 |
| 1425 | NPACT00257 | −7.4 | −58.0 | 1431 | NPACT01399 | −7.7 | −29.3 |
| 1426 | NPACT00215 | −8.5 | −49.6 | 1432 | NPACT00166 | −7.8 | −28.2 |
| 1427 | NPACT00029 | −7.4 | −49.0 | 1433 | NPACT00703 | −7.5 | −26.9 |
| 1428 | NPACT00216 | −8.3 | −45.8 | 1434 | NPACT00101 | −5.3 | −22.4 |
| 1429 | NPACT00509 | −7.8 | −35.5 |  |  |  |  |
| ***Unknown*** | | | | | | | |
| 1435 | NPACT00708 | −9.4 | −80.5 | 1451 | NPACT01574 | −8.3 | −41.7 |
| 1436 | NPACT00677 | −10.0 | −75.0 | 1452 | NPACT01502 | −8.0 | −39.0 |
| 1437 | NPACT01231 | −10.5 | −73.2 | 1453 | NPACT00796 | −7.3 | −38.4 |
| 1438 | NPACT00212 | −9.4 | −71.9 | 1454 | NPACT01543 | −8.1 | −37.8 |
| 1439 | NPACT01572 | −8.7 | −51.7 | 1455 | NPACT00884 | −9.7 | −37.2 |
| 1440 | NPACT01573 | −8.5 | −51.0 | 1456 | NPACT00028 | −7.6 | −37.0 |
| 1441 | NPACT00316 | −8.6 | −50.8 | 1457 | NPACT01499 | −7.5 | −37.0 |
| 1442 | NPACT01003 | −7.8 | −50.6 | 1458 | NPACT00779 | −8.2 | −34.9 |
| 1443 | NPACT01211 | −8.7 | −49.5 | 1459 | NPACT00317 | −7.0 | −34.2 |
| 1444 | NPACT00412 | −9.6 | −46.4 | 1460 | NPACT00474 | −6.3 | −33.5 |
| 1445 | NPACT01262 | −6.5 | −45.2 | 1461 | NPACT01295 | −8.0 | −33.0 |
| 1446 | NPACT01185 | −9.3 | −45.0 | 1462 | NPACT01356 | −8.4 | −32.9 |
| 1447 | NPACT01455 | −7.5 | −43.6 | 1463 | NPACT01404 | −8.0 | −31.1 |
| 1448 | NPACT00883 | −9.8 | −43.4 | 1464 | NPACT00795 | −6.6 | −29.0 |
| 1449 | NPACT01186 | −9.7 | −42.9 | 1465 | NPACT00668 | −6.5 | −21.6 |
| 1450 | NPACT01289 | −8.2 | −42.0 | 1466 | NPACT00174 | −5.2 | −15.7 |

**Table S1**. *Continued.*

| No. | **Compound** Name/ Code | Docking Score(kcal/mol) | **MM-GBSA//MM** binding energy (kcal/mol) | No. | **Compound** Name/ Code | Docking Score(kcal/mol) | **MM-GBSA//MM** binding energy (kcal/mol) |
| --- | --- | --- | --- | --- | --- | --- | --- |
| ***Organic Chemical*** | | | | | | | |
| 1467 | NPACT00277 | −8.3 | −63.4 | 1484 | NPACT00772 | −6.1 | −26.4 |
| 1468 | NPACT00275 | −7.7 | −58.9 | 1485 | NPACT00118 | −4.0 | −26.3 |
| 1469 | NPACT00276 | −7.9 | −58.0 | 1486 | NPACT00894 | −5.8 | −26.2 |
| 1470 | NPACT00030 | −7.2 | −54.8 | 1487 | NPACT00229 | −4.7 | −25.5 |
| 1471 | NPACT01169 | −7.6 | −50.2 | 1488 | NPACT01292 | −5.7 | −24.7 |
| 1472 | NPACT00204 | −7.2 | −47.0 | 1489 | NPACT01476 | −6.1 | −24.6 |
| 1473 | NPACT01568 | −8.1 | −46.3 | 1490 | NPACT00933 | −4.3 | −24.2 |
| 1474 | NPACT00715 | −6.4 | −43.1 | 1491 | NPACT00362 | −5.8 | −23.4 |
| 1475 | NPACT00863 | −10.3 | −42.4 | 1492 | NPACT00059 | −4.8 | −23.0 |
| 1476 | NPACT00725 | −7.8 | −40.0 | 1493 | NPACT01142 | −5.5 | −22.8 |
| 1477 | NPACT00194 | −7.8 | −37.4 | 1494 | NPACT00436 | −5.8 | −21.7 |
| 1478 | NPACT01056 | −7.4 | −32.8 | 1495 | NPACT01189 | −3.9 | −21.6 |
| 1479 | NPACT01099 | −8.1 | −32.6 | 1496 | NPACT00424 | −5.8 | −21.3 |
| 1480 | NPACT00963 | −4.4 | −30.2 | 1497 | NPACT00422 | −6.0 | −21.1 |
| 1481 | NPACT01540 | −6.4 | −28.6 | 1498 | NPACT00744 | −5.1 | −20.4 |
| 1482 | NPACT00361 | −6.1 | −27.8 | 1499 | NPACT01190 | −3.6 | −19.9 |
| 1483 | NPACT01448 | −6.0 | −26.8 |  |  |  |  |
| ***Carbohydrates*** | | | | | | | |
| 1500 | NPACT00418 | −10.2 | −127.9 | 1506 | NPACT00373 | −9.3 | −59.8 |
| 1501 | NPACT01194 | −9.8 | −88.9 | 1507 | NPACT00372 | −9.2 | −58.4 |
| 1502 | NPACT01503 | −9.7 | −76.7 | 1508 | NPACT01530 | −11.5 | −56.7 |
| 1503 | NPACT00757 | −8.9 | −71.2 | 1509 | NPACT01514 | −8.0 | −56.2 |
| 1504 | NPACT00669 | −9.1 | −65.8 | 1510 | NPACT00260 | −8.3 | −49.0 |
| 1505 | NPACT01210 | −9.0 | −72.0 |  |  |  |  |
| ***Polypyrroles*** | | | | | | | |
| 1511 | NPACT00858 | −10.6 | −66.1 | | | | |

^a^Data ranked based on the MM-GBSA//MM binding energy for each category.

**Table S2.** Estimated vina docking scores, MM**-**GBSA//MM binding energies, and MM-GBSA//MD binding energies (in kcal/mol) over 250 ps in implicit-solvent for BWQ and top 307 potent compounds against ABCG2 transporter ^a^.

| No. | **Compound** Name/ Code | Docking Score(kcal/mol) | **MM-GBSA//MM** Binding Energy (kcal/mol) | MM-GBSA//MD Binding Energy (kcal/mol) | No. | **Compound** Name/ Code | Docking Score(kcal/mol) | **MM-GBSA//MM** Binding Energy (kcal/mol) | MM-GBSA//MD Binding Energy (kcal/mol) |
| --- | --- | --- | --- | --- | --- | --- | --- | --- | --- |
|  | BWQ | −10.3 | −60.5 | −50.3 | | | | | |
|  |  | | ***Terpenoids*** | | | | | | |
| 1 | NPACT01014 | −8.1 | −95.5 | −88.3 | 41 | NPACT01459 | −9.3 | −64.8 | −53.8 |
| 2 | NPACT01016 | −8.1 | −91.4 | −88.0 | 42 | NPACT00762 | −9.0 | −68.3 | −53.3 |
| 3 | NPACT00444 | −10.0 | −114.5 | −77.6 | 43 | NPACT00998 | −9.4 | −65.2 | −53.3 |
| 4 | NPACT01557 | −10.0 | −104.0 | −77.5 | 44 | NPACT00136 | −9.2 | −68.3 | −53.2 |
| 5 | NPACT01284 | −10.7 | −73.3 | −76.3 | 45 | NPACT01172 | −10.4 | −61.5 | −53.1 |
| 6 | NPACT01552 | −10.8 | −127.2 | −73.2 | 46 | NPACT00996 | −10.1 | −61.8 | −52.7 |
| 7 | NPACT00992 | −9.3 | −85.5 | −69.3 | 47 | NPACT00116 | −10.5 | −68.4 | −52.6 |
| 8 | NPACT00834 | −10.4 | −79.8 | −68.7 | 48 | NPACT01031 | −8.5 | −61.4 | −52.6 |
| 9 | NPACT00375 | −9.8 | −72.8 | −68.1 | 49 | NPACT00141 | −8.7 | −78.6 | −52.3 |
| 10 | NPACT00470 | −10.5 | −67.6 | −66.5 | 50 | NPACT01123 | −9.2 | −61.3 | −52.1 |
| 11 | NPACT01521 | −9.9 | −72.6 | −66.0 | 51 | NPACT01307 | −9.6 | −62.2 | −51.9 |
| 12 | NPACT00406 | −10.0 | −94.5 | −66.0 | 52 | NPACT01132 | −7.8 | −70.8 | −51.1 |
| 13 | NPACT00374 | −9.8 | −74.1 | −65.5 | 53 | NPACT01082 | −9.9 | −67.9 | −51.0 |
| 14 | NPACT00728 | −8.7 | −70.8 | −64.3 | 54 | NPACT00839 | −8.9 | −62.5 | −50.8 |
| 15 | NPACT01138 | −8.9 | −72.6 | −62.7 | 55 | NPACT00997 | −10.4 | −71.5 | −50.5 |
| 16 | NPACT00833 | −10.0 | −81.1 | −62.4 | 56 | NPACT00457 | −9.1 | −62.6 | −50.4 |
| 17 | NPACT00731 | −8.7 | −70.7 | −62.2 | 57 | NPACT01522 | −8.2 | −61.7 | −50.3 |
| 18 | NPACT01494 | −10.9 | −78.4 | −62.2 | 58 | NPACT01017 | −7.3 | −70.0 | −49.6 |
| 19 | NPACT01046 | −10.5 | −61.7 | −60.9 | 59 | NPACT01173 | −10.7 | −69.4 | −48.8 |
| 20 | NPACT00122 | −10.1 | −70.3 | −60.6 | 60 | NPACT01488 | −8.8 | −62.6 | −48.8 |
| 21 | NPACT00763 | −10.3 | −75.6 | −60.3 | 61 | NPACT00592 | −8.5 | −66.9 | −48.7 |
| 22 | NPACT00115 | −8.4 | −66.5 | −59.8 | 62 | NPACT01034 | −9.9 | −65.1 | −48.6 |
| 23 | NPACT01495 | −9.9 | −83.2 | −59.7 | 63 | NPACT01444 | −7.7 | −65.8 | −48.6 |
| 24 | NPACT00461 | −9.1 | −79.6 | −59.6 | 64 | NPACT00479 | −9.5 | −63.1 | −48.3 |
| 25 | NPACT00458 | −9.4 | −62.5 | −59.6 | 65 | NPACT00135 | −9.3 | −63.2 | −47.2 |
| 26 | NPACT00936 | −9.3 | −88.8 | −59.0 | 66 | NPACT01133 | −8.3 | −60.5 | −47.1 |
| 27 | NPACT00137 | −9.1 | −74.7 | −58.7 | 67 | NPACT01207 | −9.5 | −62.9 | −47.1 |
| 28 | NPACT01137 | −8.1 | −68.0 | −58.4 | 68 | NPACT00498 | −9.1 | −61.5 | −46.6 |
| 29 | NPACT01134 | −8.2 | −76.3 | −58.3 | 69 | NPACT01171 | −11.3 | −68.8 | −45.8 |
| 30 | NPACT00469 | −10.7 | −77.1 | −57.7 | 70 | NPACT01367 | −9.1 | −70.0 | −45.4 |
| 31 | NPACT00128 | −10.4 | −69.6 | −57.4 | 71 | NPACT01203 | −8.1 | −61.1 | −45.1 |
| 32 | NPACT00125 | −9.4 | −73.5 | −56.9 | 72 | NPACT01450 | −10.0 | −65.1 | −44.4 |
| 33 | NPACT01532 | −9.8 | −61.1 | −56.4 | 73 | NPACT00095 | −10.7 | −61.1 | −43.9 |
| 34 | NPACT00931 | −10.0 | −75.7 | −56.4 | 74 | NPACT00140 | −9.2 | −77.4 | −43.5 |
| 35 | NPACT01308 | −9.6 | −73.6 | −56.3 | 75 | NPACT00403 | −8.4 | −64.9 | −41.0 |
| 36 | NPACT01120 | −9.9 | −75.0 | −56.0 | 76 | NPACT00898 | −9.4 | −63.4 | −40.2 |
| 37 | NPACT01083 | −10.7 | −64.6 | −55.8 | 77 | NPACT01449 | −9.7 | −67.9 | −39.9 |
| 38 | NPACT01135 | −8.6 | −69.6 | −55.6 | 78 | NPACT01151 | −9.2 | −64.8 | −37.4 |
| 39 | NPACT00991 | −9.3 | −65.9 | −55.3 | 79 | NPACT00138 | −8.9 | −74.0 | −36.3 |
| 40 | NPACT01139 | −7.9 | −72.8 | −54.9 |  |  |  |  |  |
|  |  | | ***Flavonoids*** | | | | | | |
| 80 | NPACT01457 | −8.8 | −97.6 | −80.6 | 85 | NPACT00343 | −9.9 | −78.4 | −58.5 |
| 81 | NPACT00340 | −9.8 | −75.3 | −67.0 | 86 | NPACT00543 | −10.3 | −62.7 | −58.2 |
| 82 | NPACT00342 | −11.5 | −65.8 | −62.7 | 87 | NPACT01309 | −10.2 | −63.8 | −55.9 |
| 83 | NPACT01312 | −10.4 | −85.2 | −61.9 | 88 | NPACT00339 | −10.0 | −84.9 | −54.9 |
| 84 | NPACT00283 | −11.1 | −66.4 | −61.1 | 89 | NPACT00977 | −9.5 | −73.6 | −54.3 |

**Table S2**. *Continued.*

| No. | **Compound** Name/ Code | | Docking Score(kcal/mol) | | MM-GBSA//MMBinding Energy (kcal/mol) | MM-GBSA//MD Binding Energy (kcal/mol) | No. | **Compound** Name/ Code | Docking Score(kcal/mol) | **MM-GBSA//MM** Binding Energy (kcal/mol) | MM-GBSA//MD Binding Energy (kcal/mol) |
| --- | --- | --- | --- | --- | --- | --- | --- | --- | --- | --- | --- |
| 90 | NPACT00809 | | −9.8 | | −67.5 | −54.0 | 104 | NPACT00680 | −10.1 | −75.0 | −50.2 |
| 91 | NPACT00878 | | −10.1 | | −69.7 | −54.0 | 105 | NPACT00288 | −10.5 | −65.6 | −49.7 |
| 92 | NPACT00381 | | −11.0 | | −70.6 | −53.5 | 106 | NPACT00807 | −9.6 | −72.5 | −48.6 |
| 93 | NPACT00494 | | −10.3 | | −62.7 | −53.3 | 107 | NPACT01553 | −9.4 | −69.4 | −47.0 |
| 94 | NPACT00176 | | −10.4 | | −61.0 | −53.0 | 108 | NPACT00384 | −10.6 | −63.7 | −46.2 |
| 95 | NPACT00545 | | −10.3 | | −71.3 | −52.9 | 109 | NPACT00386 | −10.9 | −65.4 | −44.9 |
| 96 | NPACT00195 | | −11.1 | | −66.4 | −52.6 | 110 | NPACT00542 | −10.4 | −70.7 | −44.5 |
| 97 | NPACT00189 | | −10.9 | | −64.0 | −52.4 | 111 | NPACT00553 | −9.7 | −68.7 | −44.1 |
| 98 | NPACT00540 | | −10.1 | | −65.7 | −51.8 | 112 | NPACT00256 | −11.7 | −68.3 | −43.2 |
| 99 | NPACT00511 | | −9.8 | | −70.2 | −51.5 | 113 | NPACT00918 | −10.2 | −67.6 | −41.6 |
| 100 | NPACT00541 | | −9.8 | | −70.2 | −51.4 | 114 | NPACT00808 | −10.0 | −65.2 | −41.5 |
| 101 | NPACT01315 | | −9.6 | | −60.6 | −50.4 | 115 | NPACT00800 | −9.7 | −64.9 | −39.3 |
| 102 | NPACT00660 | | −9.9 | | −69.7 | −50.3 | 116 | NPACT01318 | −10.7 | −61.4 | −34.0 |
| 103 | NPACT01554 | | −9.5 | | −70.1 | −50.2 |  |  |  |  |  |
|  | |  | | ***Steroids*** | | | | | | | |
| 117 | NPACT00011 | | −9.5 | | −86.7 | −73.4 | 127 | NPACT00865 | −10.4 | −74.3 | −57.8 |
| 118 | NPACT00866 | | −10.8 | | −86.6 | −73.1 | 128 | NPACT00015 | −10.8 | −73.3 | −56.1 |
| 119 | NPACT00622 | | −10.7 | | −75.9 | −67.1 | 129 | NPACT00505 | −10.9 | −78.9 | −54.6 |
| 120 | NPACT00013 | | −10.7 | | −91.0 | −67.1 | 130 | NPACT00536 | −9.4 | −77.5 | −54.5 |
| 121 | NPACT01019 | | −10.0 | | −84.0 | −66.2 | 131 | NPACT00049 | −9.8 | −64.2 | −52.1 |
| 122 | NPACT00504 | | −10.7 | | −93.6 | −66.1 | 132 | NPACT00331 | −9.3 | −63.9 | −51.6 |
| 123 | NPACT00507 | | −10.8 | | −79.1 | −65.2 | 133 | NPACT00124 | −11.6 | −71.4 | −49.4 |
| 124 | NPACT00014 | | −10.3 | | −87.4 | −61.9 | 134 | NPACT00012 | −10.5 | −67.4 | −46.7 |
| 125 | NPACT00016 | | −10.2 | | −75.9 | −60.6 | 135 | NPACT00949 | −10.7 | −62.2 | −43.7 |
| 126 | NPACT00811 | | −9.3 | | −60.7 | −58.6 |  |  |  |  |  |
|  | |  | | ***Simple Aromatic Natural Products*** | | | | | | | |
| 136 | NPACT00638 | | −8.1 | | −70.2 | −62.0 | 140 | NPACT01212 | −9.8 | −60.5 | −48.9 |
| 137 | NPACT00685 | | −9.1 | | −67.5 | −61.1 | 141 | NPACT00593 | −8.6 | −63.6 | −47.8 |
| 138 | NPACT00595 | | −8.9 | | −67.2 | −56.7 | 142 | NPACT00599 | −8.9 | −65.1 | −40.2 |
| 139 | NPACT00940 | | −10.6 | | −62.4 | −54.0 |  |  |  |  |  |
|  | |  | | ***Polyketides*** | | | | | | | |
| 143 | NPACT00295 | | −8.0 | | −80.7 | −83.8 | 162 | NPACT00159 | −8.5 | −83.6 | −72.7 |
| 144 | NPACT00959 | | −8.1 | | −81.6 | −82.5 | 163 | NPACT00369 | −8.3 | −86.3 | −71.8 |
| 145 | NPACT00782 | | −7.9 | | −87.1 | −82.1 | 164 | NPACT00355 | −8.3 | −85.7 | −71.8 |
| 146 | NPACT00724 | | −8.2 | | −78.1 | −81.8 | 165 | NPACT00395 | −8.0 | −83.7 | −71.5 |
| 147 | NPACT00786 | | −7.9 | | −77.8 | −80.8 | 166 | NPACT01263 | −8.2 | −78.3 | −71.5 |
| 148 | NPACT01278 | | −8.4 | | −83.2 | −80.3 | 167 | NPACT00925 | −7.6 | −85.8 | −71.3 |
| 149 | NPACT00926 | | −7.7 | | −87.0 | −78.2 | 168 | NPACT00437 | −7.7 | −86.7 | −70.9 |
| 150 | NPACT00981 | | −7.8 | | −84.6 | −77.5 | 169 | NPACT00266 | −8.0 | −84.1 | −70.9 |
| 151 | NPACT00158 | | −8.5 | | −81.6 | −77.1 | 170 | NPACT00357 | −8.4 | −88.2 | −70.6 |
| 152 | NPACT00889 | | −8.3 | | −96.8 | −75.8 | 171 | NPACT00033 | −8.1 | −86.8 | −69.9 |
| 153 | NPACT00496 | | −8.3 | | −91.6 | −75.8 | 172 | NPACT00265 | −7.6 | −77.1 | −69.6 |
| 154 | NPACT01002 | | −7.7 | | −82.7 | −75.6 | 173 | NPACT01130 | −8.1 | −81.5 | −69.4 |
| 155 | NPACT00788 | | −8.1 | | −88.3 | −75.0 | 174 | NPACT00394 | −7.5 | −84.5 | −69.2 |
| 156 | NPACT00368 | | −7.9 | | −81.0 | −74.8 | 175 | NPACT01153 | −7.7 | −82.7 | −69.0 |
| 157 | NPACT00356 | | −8.4 | | −77.6 | −74.1 | 176 | NPACT01178 | −8.1 | −76.8 | −68.9 |
| 158 | NPACT00371 | | −8.5 | | −84.4 | −73.4 | 177 | NPACT00009 | −8.2 | −78.7 | −68.8 |
| 159 | NPACT01277 | | −7.5 | | −78.4 | −73.2 | 178 | NPACT00296 | −7.7 | −78.8 | −68.4 |
| 160 | NPACT01506 | | −8.3 | | −90.3 | −73.0 | 179 | NPACT00073 | −7.6 | −85.2 | −68.4 |
| 161 | NPACT00031 | | −7.9 | | −77.5 | −72.8 | 180 | NPACT00294 | −8.1 | −86.8 | −68.3 |

**Table S2**. *Continued.*

| No. | **Compound** Name/ Code | Docking Score(kcal/mol) | MM-GBSA//MMBinding Energy (kcal/mol) | MM-GBSA//MD Binding Energy (kcal/mol) | No. | **Compound** Name/ Code | Docking Score(kcal/mol) | **MM-GBSA//MM** Binding Energy (kcal/mol) | MM-GBSA//MD Binding Energy (kcal/mol) |
| --- | --- | --- | --- | --- | --- | --- | --- | --- | --- |
| 181 | NPACT00293 | −8.0 | −73.6 | −68.3 | 205 | NPACT00624 | −8.1 | −90.6 | −64.4 |
| 182 | NPACT00952 | −8.3 | −82.9 | −68.2 | 206 | NPACT00292 | −8.0 | −92.4 | −64.3 |
| 183 | NPACT00957 | −7.9 | −81.9 | −68.1 | 207 | NPACT00157 | −8.0 | −81.3 | −64.2 |
| 184 | NPACT00625 | −8.3 | −89.6 | −67.9 | 208 | NPACT00614 | −7.8 | −84.0 | −63.9 |
| 185 | NPACT00264 | −7.8 | −79.7 | −67.7 | 209 | NPACT00438 | −7.8 | −76.8 | −63.1 |
| 186 | NPACT01157 | −9.8 | −74.5 | −67.6 | 210 | NPACT01276 | −8.1 | −79.7 | −62.8 |
| 187 | NPACT00634 | −8.0 | −80.6 | −67.5 | 211 | NPACT00956 | −8.3 | −81.3 | −62.7 |
| 188 | NPACT00635 | −8.1 | −81.8 | −67.4 | 212 | NPACT01470 | −8.2 | −77.3 | −62.5 |
| 189 | NPACT00439 | −7.6 | −79.9 | −67.4 | 213 | NPACT00787 | −7.7 | −80.3 | −62.2 |
| 190 | NPACT00780 | −7.9 | −73.3 | −67.1 | 214 | NPACT00890 | −8.3 | −80.9 | −61.6 |
| 191 | NPACT00010 | −7.7 | −89.4 | −67.1 | 215 | NPACT00087 | −8.3 | −83.7 | −61.5 |
| 192 | NPACT00032 | −8.5 | −83.3 | −67.1 | 216 | NPACT00297 | −7.5 | −89.7 | −60.7 |
| 193 | NPACT00396 | −7.7 | −81.0 | −66.9 | 217 | NPACT00370 | −7.9 | −85.3 | −60.7 |
| 194 | NPACT00672 | −8.3 | −88.2 | −66.4 | 218 | NPACT01489 | −8.1 | −77.1 | −58.7 |
| 195 | NPACT00263 | −8.2 | −86.5 | −66.0 | 219 | NPACT00267 | −7.4 | −75.3 | −58.3 |
| 196 | NPACT00955 | −8.1 | −80.9 | −65.6 | 220 | NPACT00923 | −7.9 | −84.4 | −58.2 |
| 197 | NPACT00721 | −8.4 | −88.5 | −65.6 | 221 | NPACT01177 | −7.4 | −71.3 | −57.8 |
| 198 | NPACT01511 | −8.0 | −78.2 | −65.5 | 222 | NPACT00781 | −7.7 | −85.2 | −57.3 |
| 199 | NPACT00960 | −8.3 | −89.9 | −65.3 | 223 | NPACT01279 | −8.0 | −87.8 | −56.2 |
| 200 | NPACT01152 | −7.4 | −82.7 | −65.0 | 224 | NPACT01275 | −7.8 | −61.3 | −55.3 |
| 201 | NPACT00262 | −8.3 | −96.8 | −65.0 | 225 | NPACT00722 | −8.0 | −73.7 | −53.9 |
| 202 | NPACT01280 | −8.0 | −69.3 | −64.7 | 226 | NPACT01252 | −7.6 | −80.2 | −52.2 |
| 203 | NPACT00035 | −8.4 | −78.5 | −64.7 | 227 | NPACT00723 | −8.4 | −75.2 | −49.2 |
| 204 | NPACT00785 | −8.2 | −79.5 | −64.6 | 228 | NPACT00367 | −9.0 | −63.8 | −48.0 |
| ***Polycyclic Aromatic Natural Products*** | | | | | | | | | |
| 229 | NPACT00910 | −9.3 | −63.0 | −37.4 |  |  |  |  |  |
| ***Alkaloids*** | | | | | | | | | |
| 230 | NPACT00241 | −11.3 | −88.3 | −70.5 | 235 | NPACT00186 | −10.2 | −65.1 | −50.5 |
| 231 | NPACT00488 | −9.8 | −87.5 | −63.2 | 236 | NPACT00130 | −9.6 | −68.9 | −45.8 |
| 232 | NPACT00177 | −10.0 | −70.2 | −56.9 | 237 | NPACT01007 | −7.7 | −61.3 | −45.0 |
| 233 | NPACT00131 | −10.1 | −68.5 | −56.1 | 238 | NPACT01005 | −7.4 | −67.1 | −35.2 |
| 234 | NPACT00109 | −9.9 | −62.8 | −54.9 |  |  |  |  |  |
| ***Lignans*** | | | | | | | | | |
| 239 | NPACT00501 | −11.1 | −87.9 | −62.8 | 241 | NPACT00845 | −9.1 | −74.9 | −57.5 |
| 240 | NPACT01250 | −9.0 | −74.4 | −60.3 | 242 | NPACT00274 | −9.5 | −68.0 | −46.5 |
| ***Aliphatic Natural Products*** | | | | | | | | | |
| 243 | NPACT01545 | −10.8 | −132.2 | −93.3 | 244 | NPACT01533 | −8.5 | −102.5 | −86.3 |
| ***Benzofuranoids*** | | | | | | | | | |
| 245 | NPACT00674 | −10.6 | −63.7 | −58.0 | 247 | NPACT00472 | −9.9 | −61.7 | −50.8 |
| 246 | NPACT00944 | −8.0 | −61.2 | −51.9 |  |  |  |  |  |
| ***Saponin*** | | | | | | | | | |
| 248 | NPACT01561 | −10.5 | −107.8 | −93.2 | 256 | NPACT00653 | −10.9 | −92.1 | −71.8 |
| 249 | NPACT01558 | −10.6 | −109.1 | −87.1 | 257 | NPACT00737 | −10.1 | −100.7 | −69.9 |
| 250 | NPACT01562 | −9.8 | −99.6 | −82.7 | 258 | NPACT00657 | −9.8 | −93.5 | −69.2 |
| 251 | NPACT00144 | −10.0 | −114.3 | −79.5 | 259 | NPACT00619 | −10.1 | −79.1 | −68.7 |
| 252 | NPACT01560 | −10.0 | −113.2 | −76.3 | 260 | NPACT00836 | −10.9 | −82.9 | −66.9 |
| 253 | NPACT00079 | −9.5 | −113.3 | −76.2 | 261 | NPACT00145 | −9.9 | −87.1 | −66.7 |
| 254 | NPACT00875 | −11.1 | −80.1 | −75.9 | 262 | NPACT01559 | −10.7 | −99.2 | −66.6 |
| 255 | NPACT01550 | −9.8 | −85.4 | −74.1 | 263 | NPACT01563 | −9.7 | −71.8 | −66.6 |

**Table S2**. *Continued.*

| No. | **Compound** Name/ Code | Docking Score(kcal/mol) | MM-GBSA//MMbinding energy (kcal/mol) | MM-GBSA//MD Binding Energy (kcal/mol) | No. | **Compound** Name/ Code | Docking Score(kcal/mol) | **MM-GBSA//MM** binding energy (kcal/mol) | MM-GBSA//MD Binding Energy (kcal/mol) |
| --- | --- | --- | --- | --- | --- | --- | --- | --- | --- |
| 264 | NPACT00835 | −11.4 | −96.8 | −66.5 | 278 | NPACT00081 | −10.0 | −98.2 | −61.7 |
| 265 | NPACT01548 | −9.9 | −109.3 | −66.4 | 279 | NPACT00718 | −6.3 | −75.3 | −61.0 |
| 266 | NPACT00134 | −11.8 | −99.7 | −66.4 | 280 | NPACT00082 | −9.4 | −78.9 | −60.8 |
| 267 | NPACT00616 | −10.4 | −88.8 | −66.3 | 282 | NPACT00120 | −10.5 | −71.0 | −60.4 |
| 268 | NPACT00655 | −10.1 | −85.3 | −65.7 | 283 | NPACT00646 | −9.6 | −94.9 | −60.0 |
| 269 | NPACT00119 | −9.6 | −96.1 | −65.7 | 284 | NPACT00083 | −10.2 | −79.9 | −56.3 |
| 270 | NPACT00143 | −9.3 | −84.8 | −65.7 | 285 | NPACT00656 | −11.3 | −69.5 | −56.1 |
| 271 | NPACT01546 | −10.4 | −66.1 | −64.9 | 286 | NPACT01556 | −9.2 | −85.0 | −54.9 |
| 272 | NPACT00620 | −9.5 | −67.7 | −64.3 | 287 | NPACT00645 | −9.3 | −82.9 | −54.4 |
| 273 | NPACT01564 | −9.6 | −107.5 | −64.1 | 288 | NPACT00654 | −11.7 | −73.5 | −54.1 |
| 274 | NPACT01232 | −10.2 | −87.7 | −63.4 | 289 | NPACT00142 | −9.4 | −67.7 | −54.0 |
| 275 | NPACT00618 | −9.7 | −111.6 | −62.9 | 290 | NPACT00080 | −9.8 | −68.8 | −53.3 |
| 276 | NPACT00615 | −9.9 | −74.3 | −62.6 | 291 | NPACT00078 | −9.2 | −72.6 | −52.4 |
| 277 | NPACT01547 | −9.3 | −70.2 | −62.3 | 292 | NPACT00720 | −6.0 | −81.0 | −36.5 |
| ***Tannins*** | | | | | | | | | |
| 293 | NPACT00968 | −12.0 | −150.3 | −133.4 | 294 | NPACT00416 | −9.4 | −64.6 | −60.3 |
| ***Oxygen Heterocycles*** | | | | | | | | | |
| 295 | NPACT00217 | −8.7 | −60.7 | −48.8 |  |  |  |  |  |
| ***Unknown*** | | | | | | | | | |
| 296 | NPACT01231 | −10.5 | −73.2 | −60.6 | 298 | NPACT00708 | −9.4 | −80.5 | −52.2 |
| 297 | NPACT00212 | −9.4 | −71.9 | −54.9 | 299 | NPACT00677 | −10.0 | −75.0 | −49.6 |
| ***Organic Chemical*** | | | | | | | | | |
| 300 | NPACT00277 | −8.3 | −63.4 | −45.6 | | | | | |
| ***Carbohydrates*** | | | | | | | | | |
| 301 | NPACT00418 | −10.2 | −127.9 | −96.3 | 304 | NPACT00757 | −8.9 | −71.2 | −43.4 |
| 302 | NPACT01194 | −9.8 | −88.9 | −59.7 | 305 | NPACT00669 | −9.1 | −65.8 | −42.6 |
| 303 | NPACT01503 | −9.7 | −76.7 | −45.3 | 306 | NPACT01210 | −9.0 | −72.0 | −42.1 |
| ***Polypyrroles*** | | | | | | | | | |
| 307 | NPACT00858 | −10.6 | −66.1 | −50.8 | | | | | |

^a^Data ranked based on the MM-GBSA//MD binding energy for each category.

**Table S3.** Predicted vina docking scores, MM-GBSA//MM and MM-GBSA//MD binding energies (in kcal/mol) over 250 ps and 1,000 ps in implicit-solvent for BWQ and the top 238 potent compounds towards ABCG2 transporter ^a^.

| No. | **Compound** Name/ Code | **Docking Score**  **(kcal/mol)** | **MM-GBSA//MM**  **Binding Energy (kcal/mol)** | MM-GBSA//MD Binding Energy (kcal/mol) | |
| --- | --- | --- | --- | --- | --- |
|  |  |  |  | 250 ps | 1,000 ps |
|  | BWQ | −10.3 | −60.5 | −50.3 | −49.0 |
| ***Terpenoids*** | | | | | |
| 1 | NPACT01016 | −8.1 | −91.4 | −88.0 | −87.8 |
| 2 | NPACT01014 | −8.1 | −95.5 | −88.3 | −87.5 |
| 3 | NPACT01557 | −10.0 | −104.0 | −77.5 | −79.7 |
| 4 | NPACT01284 | −10.7 | −73.3 | −76.3 | −78.2 |
| 5 | NPACT01552 | −10.8 | −127.2 | −73.2 | −75.0 |
| 6 | NPACT01521 | −9.9 | −72.6 | −66.0 | −73.7 |
| 7 | NPACT00444 | −10.0 | −114.5 | −77.6 | −72.9 |
| 8 | NPACT00992 | −9.3 | −85.5 | −69.3 | −69.4 |
| 9 | NPACT00406 | −10.0 | −94.5 | −66.0 | −68.9 |
| 10 | NPACT00728 | −8.7 | −70.8 | −64.3 | −68.4 |
| 11 | NPACT00834 | −10.4 | −79.8 | −68.7 | −67.5 |
| 12 | NPACT00470 | −10.5 | −67.6 | −66.5 | −67.1 |
| 13 | NPACT00375 | −9.8 | −72.8 | −68.1 | −66.8 |
| 14 | NPACT00374 | −9.8 | −74.1 | −65.5 | −66.7 |
| 15 | NPACT00731 | −8.7 | −70.7 | −62.2 | −66.2 |
| 16 | NPACT01495 | −9.9 | −83.2 | −59.7 | −66.0 |
| 17 | NPACT01134 | −8.2 | −76.3 | −58.3 | −65.7 |
| 18 | NPACT00936 | −9.3 | −88.8 | −59.0 | −65.4 |
| 19 | NPACT00833 | −10.0 | −81.1 | −62.4 | −65.4 |
| 20 | NPACT01494 | −10.9 | −78.4 | −62.2 | −64.2 |
| 21 | NPACT00137 | −9.1 | −74.7 | −58.7 | −64.0 |
| 22 | NPACT00125 | −9.4 | −73.5 | −56.9 | −63.6 |
| 23 | NPACT00458 | −9.4 | −62.5 | −59.6 | −63.3 |
| 24 | NPACT01138 | −8.9 | −72.6 | −62.7 | −63.2 |
| 25 | NPACT01083 | −10.7 | −64.6 | −55.8 | −63.0 |
| 26 | NPACT00122 | −10.1 | −70.3 | −60.6 | −62.1 |
| 27 | NPACT00116 | −10.5 | −68.4 | −52.6 | −60.9 |
| 28 | NPACT01459 | −9.3 | −64.8 | −53.8 | −60.6 |
| 29 | NPACT01135 | −8.6 | −69.6 | −55.6 | −60.5 |
| 30 | NPACT00469 | −10.7 | −77.1 | −57.7 | −60.5 |
| 31 | NPACT00128 | −10.4 | −69.6 | −57.4 | −60.1 |
| 32 | NPACT01139 | −7.9 | −72.8 | −54.9 | −59.0 |
| 33 | NPACT00931 | −10.0 | −75.7 | −56.4 | −58.8 |
| 34 | NPACT00115 | −8.4 | −66.5 | −59.8 | −58.5 |
| 35 | NPACT01046 | −10.5 | −61.7 | −60.9 | −58.5 |
| 36 | NPACT01308 | −9.6 | −73.6 | −56.3 | −58.2 |
| 37 | NPACT01137 | −8.1 | −68.0 | −58.4 | −57.2 |
| 38 | NPACT00763 | −10.3 | −75.6 | −60.3 | −57.2 |
| 39 | NPACT00998 | −9.4 | −65.2 | −53.3 | −57.2 |
| 40 | NPACT01532 | −9.8 | −61.1 | −56.4 | −57.1 |
| 41 | NPACT01031 | −8.5 | −61.4 | −52.6 | −56.6 |
| 42 | NPACT00762 | −9.0 | −68.3 | −53.3 | −56.2 |
| 43 | NPACT01082 | −9.9 | −67.9 | −51.0 | −56.1 |
| 44 | NPACT00461 | −9.1 | −79.6 | −59.6 | −55.5 |
| 45 | NPACT00991 | −9.3 | −65.9 | −55.3 | −55.0 |
| 46 | NPACT00996 | −10.1 | −61.8 | −52.7 | −54.8 |

**Table S3**. *Continued.*

| No. | Compound Name/ Code | **Docking Score**  **(kcal/mol)** | **MM-GBSA//MM**  **Binding Bnergy (kcal/mol)** | MM-GBSA//MD Binding Energy (kcal/mol) | |
| --- | --- | --- | --- | --- | --- |
|  |  |  |  | 250 ps | 1,000 ps |
| 47 | NPACT01120 | −9.9 | −75.0 | −56.0 | −54.6 |
| 48 | NPACT01172 | −10.4 | −61.5 | −53.1 | −53.6 |
| 49 | NPACT00997 | −10.4 | −71.5 | −50.5 | −53.3 |
| 50 | NPACT01123 | −9.2 | −61.3 | −52.1 | −53.0 |
| 51 | NPACT00141 | −8.7 | −78.6 | −52.3 | −52.5 |
| 52 | NPACT01307 | −9.6 | −62.2 | −51.9 | −51.9 |
| 53 | NPACT00136 | −9.2 | −68.3 | −53.2 | −51.5 |
| 54 | NPACT01132 | −7.8 | −70.9 | −51.1 | −51.4 |
| 55 | NPACT00457 | −9.1 | −62.6 | −50.4 | −50.8 |
| 56 | NPACT00839 | −8.9 | −62.6 | −50.8 | −50.1 |
| ***Flavonoids*** | | | | | |
| 57 | NPACT01457 | −8.8 | −97.6 | −80.6 | −91.2 |
| 58 | NPACT00283 | −11.1 | −66.4 | −61.1 | −66.8 |
| 59 | NPACT00342 | −11.5 | −65.8 | −62.7 | −65.3 |
| 60 | NPACT00340 | −9.8 | −75.3 | −67.0 | −64.4 |
| 61 | NPACT00343 | −9.9 | −78.4 | −58.5 | −64.1 |
| 62 | NPACT00339 | −10.0 | −84.9 | −54.9 | −62.9 |
| 63 | NPACT00543 | −10.3 | −62.7 | −58.2 | −61.8 |
| 64 | NPACT00809 | −9.8 | −67.5 | −54.0 | −59.9 |
| 65 | NPACT00494 | −10.3 | −62.7 | −53.3 | −58.0 |
| 66 | NPACT00189 | −10.9 | −64.0 | −52.4 | −57.7 |
| 67 | NPACT00540 | −10.1 | −65.7 | −51.8 | −56.8 |
| 68 | NPACT01309 | −10.2 | −63.8 | −55.9 | −56.6 |
| 69 | NPACT00511 | −9.8 | −70.2 | −51.5 | −56.2 |
| 70 | NPACT00545 | −10.3 | −71.3 | −52.9 | −56.0 |
| 71 | NPACT00195 | −11.1 | −66.4 | −52.6 | −55.2 |
| 72 | NPACT00878 | −10.1 | −69.7 | −54.0 | −54.6 |
| 73 | NPACT01312 | −10.4 | −85.2 | −61.9 | −53.7 |
| 74 | NPACT00176 | −10.4 | −61.0 | −53.0 | −52.4 |
| 75 | NPACT00381 | −11.0 | −70.6 | −53.5 | −52.1 |
| 76 | NPACT00977 | −9.5 | −73.6 | −54.3 | −47.9 |
| ***Steroids*** | | | | | |
| 77 | NPACT00866 | −10.8 | −86.6 | −73.1 | −78.7 |
| 78 | NPACT00011 | −9.5 | −86.7 | −73.4 | −74.9 |
| 79 | NPACT00013 | −10.7 | −91.0 | −67.1 | −71.9 |
| 80 | NPACT00622 | −10.7 | −75.9 | −67.1 | −67.8 |
| 81 | NPACT00015 | −10.8 | −73.3 | −56.1 | −66.6 |
| 82 | NPACT00504 | −10.7 | −93.6 | −66.1 | −65.8 |
| 83 | NPACT00014 | −10.3 | −87.4 | −61.9 | −64.1 |
| 84 | NPACT01019 | −10.0 | −84.0 | −66.2 | −63.7 |
| 85 | NPACT00507 | −10.8 | −79.1 | −65.2 | −60.5 |
| 86 | NPACT00865 | −10.4 | −74.3 | −57.8 | −59.4 |
| 87 | NPACT00016 | −10.2 | −75.9 | −60.6 | −57.2 |
| 88 | NPACT00811 | −9.3 | −60.7 | −58.6 | −56.6 |
| ***Simple Aromatic Natural Products*** | | | | | |
| 89 | NPACT00685 | −9.1 | −67.5 | −61.1 | −66.6 |
| 90 | NPACT00638 | −8.1 | −70.2 | −62.0 | −60.1 |
| 91 | NPACT00595 | −8.9 | −67.2 | −56.7 | −58.1 |
| 92 | NPACT00940 | −10.6 | −62.4 | −54.0 | −57.0 |

**Table S3**. *Continued.*

| No. | Compound Name/ Code | **Docking Score**  **(kcal/mol)** | **MM-GBSA//MM**  **Binding Bnergy (kcal/mol)** | MM-GBSA//MD Binding Energy (kcal/mol) | |
| --- | --- | --- | --- | --- | --- |
|  |  |  |  | 250 ps | 1,000 ps |
| ***Polyketides*** | | | | | |
| 93 | NPACT00724 | −8.2 | −78.1 | −81.8 | −91.1 |
| 94 | NPACT00295 | −8.0 | −80.7 | −83.8 | −90.0 |
| 95 | NPACT00981 | −7.8 | −84.6 | −77.5 | −89.1 |
| 96 | NPACT00959 | −8.1 | −81.6 | −82.5 | −85.5 |
| 97 | NPACT00786 | −7.9 | −77.8 | −80.8 | −84.8 |
| 98 | NPACT00782 | −7.9 | −87.1 | −82.1 | −84.5 |
| 99 | NPACT00889 | −8.3 | −96.8 | −75.8 | −83.8 |
| 100 | NPACT01278 | −8.4 | −83.2 | −80.3 | −83.5 |
| 101 | NPACT00158 | −8.5 | −81.6 | −77.1 | −82.2 |
| 102 | NPACT00031 | −7.9 | −77.5 | −72.8 | −81.4 |
| 103 | NPACT00925 | −7.6 | −85.8 | −71.3 | −81.2 |
| 104 | NPACT00496 | −8.3 | −91.6 | −75.8 | −79.7 |
| 105 | NPACT00033 | −8.1 | −86.8 | −69.9 | −79.5 |
| 106 | NPACT00294 | −8.1 | −86.8 | −68.3 | −79.4 |
| 107 | NPACT00371 | −8.5 | −84.4 | −73.4 | −79.0 |
| 108 | NPACT00356 | −8.4 | −77.6 | −74.1 | −78.4 |
| 109 | NPACT00159 | −8.5 | −83.6 | −72.7 | −78.3 |
| 110 | NPACT00357 | −8.4 | −88.2 | −70.6 | −77.5 |
| 111 | NPACT00625 | −8.3 | −89.6 | −67.9 | −76.4 |
| 112 | NPACT01277 | −7.5 | −78.4 | −73.2 | −76.3 |
| 113 | NPACT00788 | −8.1 | −88.3 | −75.0 | −75.5 |
| 114 | NPACT00395 | −8.0 | −83.7 | −71.5 | −75.1 |
| 115 | NPACT00009 | −8.2 | −78.7 | −68.8 | −74.4 |
| 116 | NPACT01002 | −7.7 | −82.7 | −75.6 | −73.8 |
| 117 | NPACT01153 | −7.7 | −82.7 | −69.0 | −73.6 |
| 118 | NPACT01511 | −8.0 | −78.2 | −65.5 | −73.6 |
| 119 | NPACT00955 | −8.1 | −80.9 | −65.6 | −73.5 |
| 120 | NPACT00296 | −7.7 | −78.8 | −68.4 | −73.5 |
| 121 | NPACT01130 | −8.1 | −81.5 | −69.4 | −73.0 |
| 122 | NPACT00368 | −7.9 | −81.0 | −74.8 | −72.7 |
| 123 | NPACT00952 | −8.3 | −82.9 | −68.2 | −72.6 |
| 124 | NPACT01157 | −9.8 | −74.5 | −67.6 | −72.3 |
| 125 | NPACT00926 | −7.7 | −87.0 | −78.2 | −72.2 |
| 126 | NPACT00265 | −7.6 | −77.1 | −69.6 | −72.1 |
| 127 | NPACT00780 | −7.9 | −73.3 | −67.1 | −72.1 |
| 128 | NPACT00394 | −7.5 | −84.5 | −69.2 | −71.4 |
| 129 | NPACT00266 | −8.0 | −84.1 | −70.9 | −71.4 |
| 130 | NPACT01178 | −8.1 | −76.8 | −68.9 | −71.2 |
| 131 | NPACT00396 | −7.7 | −81.0 | −66.9 | −71.1 |
| 132 | NPACT00293 | −8.0 | −73.6 | −68.3 | −71.0 |
| 133 | NPACT00292 | −8.0 | −92.4 | −64.3 | −70.9 |
| 134 | NPACT00010 | −7.7 | −89.4 | −67.1 | −70.6 |
| 135 | NPACT00437 | −7.7 | −86.7 | −70.9 | −70.6 |
| 136 | NPACT01506 | −8.3 | −90.3 | −73.0 | −70.5 |
| 137 | NPACT00957 | −7.9 | −81.9 | −68.1 | −70.5 |
| 138 | NPACT00369 | −8.3 | −86.3 | −71.8 | −70.5 |
| 139 | NPACT00672 | −8.3 | −88.2 | −66.4 | −70.5 |
| 140 | NPACT01489 | −8.1 | −77.1 | −58.7 | −69.9 |
| 141 | NPACT00035 | −8.4 | −78.5 | −64.7 | −69.4 |

**Table S3**. *Continued.*

| No. | Compound Name/ Code | **Docking Score**  **(kcal/mol)** | **MM-GBSA//MM**  **binding energy (kcal/mol)** | MM-GBSA//MD Binding Energy (kcal/mol) | |
| --- | --- | --- | --- | --- | --- |
|  |  |  |  | 250 ps | 1,000 ps |
| 142 | NPACT00787 | −7.7 | −80.3 | −62.2 | −69.3 |
| 143 | NPACT01152 | −7.4 | −82.7 | −65.0 | −69.1 |
| 144 | NPACT00634 | −8.0 | −80.6 | −67.5 | −68.6 |
| 145 | NPACT00264 | −7.8 | −79.7 | −67.7 | −68.3 |
| 146 | NPACT00635 | −8.1 | −81.8 | −67.4 | −67.7 |
| 147 | NPACT00439 | −7.6 | −79.9 | −67.4 | −67.4 |
| 148 | NPACT00370 | −7.9 | −85.3 | −60.7 | −66.8 |
| 149 | NPACT01470 | −8.2 | −77.3 | −62.5 | −66.8 |
| 150 | NPACT01276 | −8.1 | −79.7 | −62.8 | −66.8 |
| 151 | NPACT01263 | −8.2 | −78.3 | −71.5 | −66.7 |
| 152 | NPACT00721 | −8.4 | −88.5 | −65.6 | −66.4 |
| 153 | NPACT00624 | −8.1 | −90.6 | −64.4 | −65.9 |
| 154 | NPACT00087 | −8.3 | −83.7 | −61.5 | −65.6 |
| 155 | NPACT00960 | −8.3 | −89.9 | −65.3 | −65.4 |
| 156 | NPACT00262 | −8.3 | −96.8 | −65.0 | −65.1 |
| 157 | NPACT00157 | −8.0 | −81.3 | −64.2 | −65.1 |
| 158 | NPACT01280 | −8.0 | −69.3 | −64.7 | −64.8 |
| 159 | NPACT00438 | −7.8 | −76.8 | −63.1 | −64.3 |
| 160 | NPACT00032 | −8.5 | −83.3 | −67.1 | −64.2 |
| 161 | NPACT00614 | −7.8 | −84.0 | −63.9 | −63.8 |
| 162 | NPACT00956 | −8.3 | −81.3 | −62.7 | −63.3 |
| 163 | NPACT00890 | −8.3 | −80.9 | −61.6 | −63.0 |
| 164 | NPACT00785 | −8.2 | −79.5 | −64.6 | −62.5 |
| 165 | NPACT00781 | −7.7 | −85.2 | −57.3 | −61.8 |
| 166 | NPACT00923 | −7.9 | −84.4 | −58.2 | −61.5 |
| 167 | NPACT01177 | −7.4 | −71.3 | −57.8 | −61.5 |
| 168 | NPACT00263 | −8.2 | −86.5 | −66.0 | −61.3 |
| 169 | NPACT00297 | −7.5 | −89.7 | −60.7 | −60.8 |
| 170 | NPACT01275 | −7.8 | −61.3 | −55.3 | −59.7 |
| 171 | NPACT00267 | −7.4 | −75.3 | −58.3 | −59.2 |
| 172 | NPACT01252 | −7.6 | −80.2 | −52.2 | −58.6 |
| 173 | NPACT00722 | −8.0 | −73.7 | −53.9 | −56.0 |
| 174 | NPACT01279 | −8.0 | −87.8 | −56.2 | −52.7 |
| ***Alkaloids*** | | | | | |
| 175 | NPACT00488 | −9.8 | 87.5 | −63.2 | −73.8 |
| 176 | NPACT00241 | −11.3 | 88.3 | −70.5 | −66.3 |
| 177 | NPACT00186 | −10.2 | 65.1 | −50.5 | −56.9 |
| 178 | NPACT00177 | −10.0 | 70.2 | −56.9 | −56.7 |
| 179 | NPACT00131 | −10.1 | 68.5 | −56.1 | −55.1 |
| 180 | NPACT00109 | −9.9 | 62.8 | −54.9 | −49.2 |
| ***Saponin*** | | | | | |
| 181 | NPACT01558 | −10.6 | −109.1 | −87.1 | −89.5 |
| 182 | NPACT01561 | −10.5 | −107.8 | −93.2 | −87.8 |
| 183 | NPACT01562 | −9.8 | −99.6 | −82.7 | −85.0 |
| 184 | NPACT00144 | −10.0 | −114.3 | −79.5 | −82.1 |
| 185 | NPACT01560 | −10.0 | −113.2 | −76.3 | −81.0 |
| 186 | NPACT01550 | −9.8 | −85.4 | −74.1 | −79.2 |
| 187 | NPACT01564 | −9.6 | −107.5 | −64.1 | −79.0 |
| 188 | NPACT00875 | −11.1 | −80.1 | −75.9 | −77.1 |
| 189 | NPACT01563 | −9.7 | −71.8 | −66.6 | −76.4 |
| 190 | NPACT01559 | −10.7 | −99.2 | −66.6 | −76.0 |

**Table S3**. *Continued.*

| No. | Compound Name/ Code | **Docking Score**  **(kcal/mol)** | **MM-GBSA//MM**  **binding energy (kcal/mol)** | MM-GBSA//MD Binding Energy (kcal/mol) | |
| --- | --- | --- | --- | --- | --- |
|  |  |  |  | 250 ps | 1,000 ps |
| 191 | NPACT00079 | −9.5 | −113.3 | −76.2 | −75.3 |
| 192 | NPACT00653 | −10.9 | −92.1 | −71.8 | −74.5 |
| 193 | NPACT00657 | −9.8 | −93.5 | −69.2 | −73.6 |
| 194 | NPACT00616 | −10.4 | −88.8 | −66.3 | −70.4 |
| 195 | NPACT00646 | −9.6 | −94.9 | −60.0 | −70.4 |
| 196 | NPACT00143 | −9.3 | −84.8 | −65.7 | −70.2 |
| 197 | NPACT00835 | −11.4 | −96.8 | −66.5 | −69.8 |
| 198 | NPACT01548 | −9.9 | −109.3 | −66.4 | −69.7 |
| 199 | NPACT00619 | −10.1 | −79.1 | −68.7 | −69.6 |
| 200 | NPACT00145 | −9.9 | −87.1 | −66.7 | −69.1 |
| 201 | NPACT00082 | −9.4 | −78.9 | −60.8 | −68.7 |
| 202 | NPACT00620 | −9.5 | −67.7 | −64.3 | −68.6 |
| 203 | NPACT00836 | −10.9 | −82.9 | −66.9 | −66.3 |
| 204 | NPACT00737 | −10.1 | −100.7 | −69.9 | −65.9 |
| 205 | NPACT00081 | −10.0 | −98.2 | −61.7 | −65.8 |
| 206 | NPACT01546 | −10.4 | −66.1 | −64.9 | −65.5 |
| 207 | NPACT00615 | −9.9 | −74.3 | −62.6 | −65.1 |
| 208 | NPACT00618 | −9.7 | −111.6 | −62.9 | −65.0 |
| 209 | NPACT00119 | −9.6 | −96.1 | −65.7 | −64.5 |
| 210 | NPACT01232 | −10.2 | −87.7 | −63.4 | −64.2 |
| 211 | NPACT00120 | −10.5 | −71.0 | −60.4 | −64.2 |
| 212 | NPACT00134 | −11.8 | −99.7 | −66.4 | −64.0 |
| 213 | NPACT00080 | −9.8 | −68.8 | −53.3 | −63.5 |
| 214 | NPACT01547 | −9.3 | −70.2 | −62.3 | −63.5 |
| 215 | NPACT00655 | −10.1 | −85.3 | −65.7 | −62.1 |
| 216 | NPACT01556 | −9.2 | −85.0 | −54.9 | −61.6 |
| 217 | NPACT00645 | −9.3 | −82.9 | −54.4 | −60.6 |
| 218 | NPACT00656 | −11.3 | −69.5 | −56.1 | −60.0 |
| 219 | NPACT00083 | −10.2 | −79.9 | −56.3 | −56.8 |
| 220 | NPACT00654 | −11.7 | −73.5 | −54.1 | −56.8 |
| 221 | NPACT00718 | −6.3 | −75.3 | −61.0 | −56.8 |
| 222 | NPACT00078 | −9.2 | −72.6 | −52.4 | −56.0 |
| 223 | NPACT00142 | −9.4 | −67.7 | −54.0 | −55.2 |
| ***Lignans*** | | | | | |
| 224 | NPACT01250 | −9.0 | −74.4 | −60.3 | −62.3 |
| 225 | NPACT00501 | −11.1 | −87.9 | −62.8 | −61.7 |
| 226 | NPACT00845 | −9.1 | −74.9 | −57.5 | −60.8 |
| ***Aliphatic Natural Products*** | | | | | |
| 227 | NPACT01545 | −10.8 | −132.2 | −93.3 | −100.2 |
| 228 | NPACT01533 | −8.5 | −102.5 | −86.3 | −90.0 |
| ***Benzofuranoids*** | | | | | |
| 229 | NPACT00674 | −10.6 | −63.7 | −58.0 | −56.5 |
| 230 | NPACT00944 | −8.0 | −61.2 | −51.9 | −55.8 |
| 231 | NPACT00472 | −9.9 | −61.7 | −50.8 | −49.7 |
| ***Tannins*** | | | | | |
| 232 | NPACT00968 | −12.0 | −150.3 | −133.4 | −126.3 |
| 233 | NPACT00416 | −9.4 | −64.6 | −60.3 | −66.5 |
| ***Unknown*** | | | | | |
| 234 | NPACT01231 | −10.5 | −73.2 | −60.6 | −61.0 |
| 235 | NPACT00708 | −9.4 | −80.5 | −52.2 | −53.8 |

**Table S3**. *Continued.*

| No. | Compound Name/ Code | **Docking Score**  **(kcal/mol)** | **MM-GBSA//MM**  **binding energy (kcal/mol)** | MM-GBSA//MD Binding Energy (kcal/mol) | |
| --- | --- | --- | --- | --- | --- |
|  |  |  |  | 250 ps | 250 ps |
| 236 | NPACT00212 | −9.4 | −71.9 | −54.9 | −53.6 |
| ***Carbohydrates*** | | | | | |
| 237 | NPACT00418 | −10.2 | −127.9 | −96.3 | −95.5 |
| 238 | NPACT01194 | −9.8 | −88.9 | −59.7 | −62.9 |

^a^Data sorted according to MM-GBSA binding energy over 1,000 ps for each category.
